# Microglial CD31 suppresses Aβ clearance and promotes Alzheimer pathology in 5×FAD mice

**DOI:** 10.1038/s41467-026-74037-5

**Published:** 2026-06-05

**Authors:** Qiuzhi Zhou, Fei Sun, Yao Zhang, Xiaojian Cao, Mengzhu Li, Haitao Yu, Tao Jiang, Shihong Li, Weixia Wang, Jiazhao Xie, Ting He, Yanchao Liu, Dan Ke, Xiao-Chuan Wang, Peng Xu, Enjie Liu, Hong Chen, Jian-Zhi Wang

**Affiliations:** 1 Key Laboratory of Education Ministry of China/Hubei Province for Neurological Disorders, Department of Pathophysiology, School of Basic Medicine, Tongji Medical College, Huazhong University of Science and Technology, Wuhan, China; 2 Department of Rehabilitation, Tongji Hospital, Tongji Medical College, Huazhong University of Science and Technology, Wuhan, China; 3 Stem Cell Research Center, Tongji Hospital, Tongji Medical College, Huazhong University of Science and Technology, Wuhan, China; 4 Department of Endocrinology, Key Laboratory of Ministry of Education for Neurological Disorders, Li Yuan Hospital, Tongji Medical College, Huazhong University of Science and Technology, Wuhan, China; 5 Department of Neurosurgery, The Central Hospital of Wuhan, Tongji Medical College, Huazhong University of Science and Technology, Wuhan, China; 6 Department of Neurosurgery, Tongji Hospital, Tongji Medical College, Huazhong University of Science and Technology, Wuhan, China; 7 Center of Clinical Laboratory Medicine, Zhongda Hospital, School of Medicine, Advanced Institute for Life and Health, Southeast University, Nanjing, China; 8 Department of Pathology, The First Affiliated Hospital of Zhengzhou University, Zhengzhou, China; 9 Hubei Key Laboratory of Cognitive and Affective Disorders, Jianghan University, Wuhan, China

**Keywords:** Alzheimer's disease, Alzheimer's disease

## Abstract

Microglia play crucial roles in Alzheimer’s disease (AD), yet the molecular mechanisms are unclear. Here, we show that CD31, a recognized endothelial marker, is predominantly expressed in microglia but not in neurons or astrocytes, and it is significantly elevated in the brains of AD patients and mouse models. Microglia-specific CD31 knockdown in 5xFAD mice substantially attenuated the dysregulated transcription networks, suppressed microglia hyperactivation and the disease-associated microglia (DAM), mitigated Aβ deposition and inflammation, and eventually improved cognitive functions in mice. Mechanistically, CD31 knockdown damaged the simultaneous recruitment of Src homology phosphatase 2 (SHP2) and STAT3, leading to a reduced dephosphorylation and enhanced activation of STAT3, a transcription factor. STAT3 activation increased transcription of membrane metalloendopeptidase (MME) and promoted Aβ clearance. Collectively, this study identifies microglial CD31, by regulating SHP2–STAT3–MME axis, plays a role in AD pathogenesis and targeting CD31 is promising in AD drug development.

## Abstract (web summary)

Microglial CD31 is increased in Alzheimer’s disease and limits amyloid clearance. Reducing CD31 enhances Aβ degradation, alleviates pathology, and improves cognitive function in mouse models.

## Introduction

Alzheimer’s disease (AD) is the most common neurodegenerative disorder. With the population aging, the prevalence of AD is rising rapidly, which significantly impairs the life quality of the elderly and imposes a heavy burden on families and society. Pathologically, AD is characterized by the massive production of extracellular plaques mainly composed of β-amyloid (Aβ) and intracellular neurofibrillary tangles (NFTs) resulting from hyperphosphorylated Tau protein^1^. Microglia, the primary immune cells in the brain, play critical roles in brain physiopathology, including development, synaptic formation, and responses to disease, by altering their morphological and functional states^2–5^. Driven by misfolded proteins and chronic immune challenges, microglial activation is thought to accelerate disease progression^6–8^. Extensive studies highlight the interaction between microglia and amyloid-β (Aβ) or Tau as fundamental to AD pathology. Upon Aβ plaque formation, microglia are recruited to initiate CD33-dependent phagocytosis^9^. Overexpression of *Gas6*—a major ligand for the TAM (Tyro3, Axl, and MerTK) receptors in the central nervous system (CNS)—can activate microglial phagocytosis to clear Aβ in the brains of AD model mice^10,11^. Microglial activation also promotes AD-like Tau spreading, whereas microglial deactivation can mitigate Tau propagation and improve cognitive function^12–14^. Emerging research underscores the importance of microglia-specific proteins, such as TREM2, which mediate these effects by regulating inflammatory responses and enhancing Aβ clearance via phagocytic pathways^15–17^. These findings emphasize the critical role of microglia in AD progression and the potential of targeting microglial pathways to modify disease outcomes.

CD31, a transmembrane glycoprotein encoded by chromosome 17 and a member of the immunoglobulin superfamily of cell adhesion molecules, is primarily reported to be expressed in vascular endothelial cells, platelets, and leukocytes. Aberrant CD31 expression has been linked to inflammation, endotheliomas, atherosclerosis, and hematologic conditions. Additionally, CD31 is abundantly expressed at endothelial cell junctions, where it functions in leukocyte trafficking and maintenance of endothelial integrity^18^.

Notably, accumulating evidence indicates a close association between CD31 and AD onset and progression. Clinical studies have shown that CD31 protein levels are significantly elevated in the plasma and cerebellar tissues of AD patients compared with age-matched controls^19,20^. In parallel, multiple transcriptomic analyses demonstrate that CD31 mRNA expression is markedly increased across several brain regions in AD patients^21,22^. In experimental models, injection of Aβ₁_–_₄₂ into the hippocampal CA1 region of mice results in a significant increase in CD31 expression in the brain^23^, which further supports a link between Aβ-related pathological stimulation and CD31 upregulation. Collectively, these findings suggest that CD31 may play an important role in AD evolution.

Although CD31 has long been regarded as a vascular-associated molecule, recent single-cell transcriptomic studies reveal substantial CD31 expression in microglia within the human brain, with pronounced upregulation in microglia from AD patients^21,22,24^. Consistent with these observations, previous transcriptomic data showed that treatment of human iPSC-derived microglia with oligomeric Aβ led to a significant increase in CD31 transcripts^24^. These results suggest that aberrant CD31 expression in microglia is closely associated with AD-related pathology and raise the possibility that CD31 may exert additional functions in AD pathogenesis. However, it should be noted that these observations are largely derived from large-scale bioinformatic transcriptomic and single-cell analyses; experimental validation of CD31 expression in microglia is still lacking, and its precise functional role and underlying mechanisms in AD pathology remain to be elucidated.

In the present study, we confirmed that CD31 in the brain is dominantly expressed in microglia rather than in neurons or astrocytes under the same conditions. Furthermore, CD31 expression levels were significantly elevated in both AD patients and transgenic mouse models. Microglia-specific knockdown of CD31 in 5×FAD mice significantly improved neuronal plasticity and cognitive functions, accompanied by significantly enhanced Aβ clearance. Mechanistically, CD31 modulates MME expression by recruiting both the transcription factor STAT3 and the protein phosphatase SHP2, thereby regulating activity-dependent phosphorylation of STAT3 and subsequent MME expression.

## Results

### Microglial CD31 is upregulated in AD patients and mouse models, and its microglia-specific knockdown improves cognition in 5×FAD mice

**Fig. 1:**
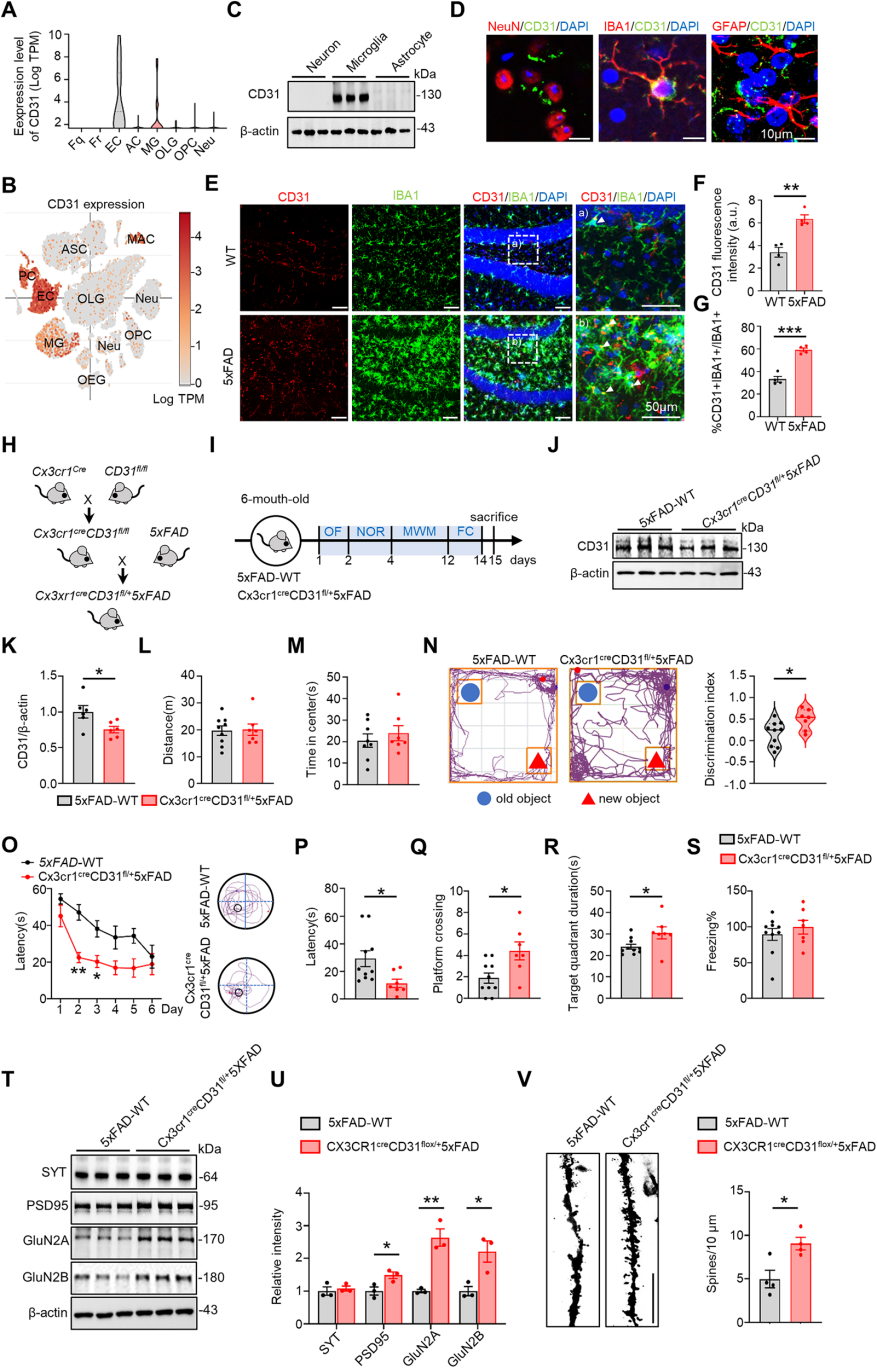
CD31 is highly expressed in microglia, and its knockdown ameliorates cognitive impairment in 5×FAD mice. Public single-cell RNA-sequencing datasets from human (**A**) and mouse brain (**B**) tissues show abundant CD31 expression in endothelial cells and microglia. Western blotting (**C**) and immunofluorescence (**D**) confirmed CD31 expression in microglia, but not in astrocytes or neurons. Representative blots and images from three independent experiments with similar results are shown. Bar = 10 μm. Immunofluorescence staining of the hippocampus (**E**) revealed increased CD31 abundance (**F**) and a higher proportion of CD31-positive microglia (**G**) in 5×FAD mice compared with WT mice. *n* = 4 mice per group, two-sided unpaired Student’s *t* test, **p = 0.0019, ***p = 0.0002 vs. WT. Bar = 50 μm. **H** Schematic of the breeding strategy for *Cx3cr1*^Cre^CD31^fl/+^5×FAD mice. **I** Flowchart depicting the behavioral testing schedule. **J**, **K** Western blotting detected ~25% reduction in hippocampal CD31 levels in *Cx3cr1*^Cre^CD31^fl/+^5×FAD mice. *n* = 6 mice per group, two-sided unpaired Student’s *t* test, *p = 0.0301 vs. 5×FAD-WT. **L**–**S** Behavioral assessments. No effect on locomotion (**L**) or center time (**M**) in OF, two-sided unpaired Student’s *t* test. Increased discrimination index (**N**) in NOR two-sided unpaired Student’s *t* test, *p = 0.0245 vs 5×FAD-WT. Reduced escape latency during training (**O**) in MWM, two-way RM ANOVA with Šídák post hoc test, **p = 0.0013, *p = *0.0339* vs. 5×FAD-WT; improved probe performance with decreased latency (**P**), increased platform crossings (**Q**), and increased target quadrant time (**R**), two-sided unpaired Student’s *t* test, *p = 0.0272 (**P**), *p = 0.0136 (**Q**), *p = 0.0329 (**R**) vs. 5×FAD-WT. No difference in freezing time (**S**) in FC, two-sided unpaired Student’s *t* test. *n* = 7 (*Cx3cr1*^Cre^CD31^fl/+^5×FAD) and 10 (5×FAD-WT) mice per group. **T**, **U** Western blotting shows increased PSD95, GluN2A, and GluN2B levels after CD31 knockdown. *n* = 3 mice per group, two-sided unpaired Student’s *t* test, *p = 0.0370 (PSD95), **p = 0.0038 (GluN2A), and *p = 0.0265 (GluN2B) vs. 5×FAD-WT. **V** Dendritic spine density in hippocampal neurons assessed by Golgi staining. *n* = 4 mice per group, two-sided unpaired Student’s *t* test, *p = 0.0171 vs. 5×FAD-WT. Data were presented as mean ± SEM. Source data are provided as a Source data file.

To characterize the expression pattern of CD31 in the brain, we first analyzed single-cell RNA sequencing datasets from the cerebral cortex of AD patients (http://www.alzdata.org/)^25^ and mice (https://singlecell.broadinstitute.org/)^26^. These analyses revealed high CD31 expression in microglia, in addition to endothelial cells (Fig. 1A, B). To experimentally validate this, we assessed CD31 expression across neural cell types using Western blotting, which detected robust CD31 expression in primary microglia but not in primary astrocytes or neurons (Fig. 1C). Immunofluorescence staining further confirmed co-localization of CD31 with the microglial marker IBA1, with no co-localization with the neuronal marker NeuN or astrocytic marker GFAP (Fig. 1D). Additionally, both CD31 expression levels and the proportion of CD31⁺ microglia within the total microglial population were significantly increased in 5×FAD mice compared to wild-type controls (Fig. 1E–G). Exposure of BV-2 cells (Supplementary Fig. 1A–C) or primary microglia (Supplementary Fig. 1D–F) to Aβ₁₋₄₂ oligomers (5 µM) also induced upregulated CD31 expression. Collectively, these results demonstrate that microglia abundantly express CD31 and that Aβ promotes microglial CD31 upregulation in 5×FAD mice.

To investigate the role of CD31 in microglia, we first generated CD31^fl/fl^ mice carrying floxed CD31 alleles and crossed them with *Cx3cr1*-Cre mice to obtain microglia-specific CD31 knockout mice (*Cx3cr1*^Cre^CD31^fl/fl^). Using this model, we examined the contribution of microglial CD31 to the total CD31 expression in the brain. Western blot analysis showed that deletion of CD31 in microglia led to a approximate 19% reduction in total CD31 protein levels in the hippocampus (Supplementary Fig. 2A, B), indicating that microglia account for roughly one-fifth of the overall CD31 expression in the brain. Additionally, we isolated and cultured primary microglia from *Cx3cr1*^Cre^CD31^fl/+^ mice and confirmed that CD31 protein levels were reduced by approximately 47% compared with controls (Supplementary Fig. 2C, D), further validating that the generation of microglia-specific CD31 knockdown mice was successful.

To explore the role of elevated microglial CD31 in AD progression, we generated a microglia-specific CD31 knockdown model on an AD background (*Cx3cr1*^Cre^CD31^fl/+^5×FAD) by crossing CD31^fl/fl^, *Cx3cr1*-Cre, and 5×FAD mice (Fig. 1H). To investigate the impact of microglia-specific CD31 knockdown on cognitive function in 5×FAD mice, we performed a battery of behavioral tests on 6-month-old 5×FAD-WT and *Cx3cr1*^Cre^CD31^fl/+^5×FAD mice (Fig.1I). Western blot analysis revealed that CD31 expression in the hippocampus was reduced by approximately 25% in *Cx3cr1*^Cre^CD31^fl/+^5×FAD mice compared to 5×FAD-WT controls (Fig. 1J, K), confirming successful downregulation of CD31 in microglia. This reduction was greater than that observed in *Cx3cr1*^Cre^CD31^fl/fl^ mice without the AD background, suggesting that the higher knockdown efficiency may result from an elevated CD31 expression in AD microglia and/or a potential decrease in the overall number of microglia.

The open field (OF) test showed no difference in total distance traveled (Fig. 1L) and the time spent in the center area (Fig. 1M) between *Cx3cr1*^Cre^CD31^fl/+^5×FAD and 5×FAD-WT mice, indicating that microglial CD31 knockdown did not affect general activity or anxiety-like behavior. In the novel object recognition (NOR) test, the discrimination index significantly improved in the *Cx3cr1*^Cre^CD31^fl/+^5×FAD group compared to the 5×FAD-WT group (Fig. 1N), reflecting an improved cognitive function. In the Morris water maze (MWM) test, *Cx3cr1*^Cre^CD31^fl/+^5×FAD mice exhibited decreased latency in locating the hidden platform during the training phase (Fig. 1O), and they demonstrated reduced latency (Fig. 1P), increased platform crossings during the probe trial (Fig. 1Q) and extended time spent in the target quadrant (Fig. 1R), suggesting enhanced spatial learning and memory abilities. The contextual fear conditioning (FC) test showed no difference in freezing time, confirming that microglia-specific CD31 knockdown did not affect fear memory (Fig.1S). Consistent with the behavioral improvements, Western blot analysis revealed a significant increase in postsynaptic proteins, including PSD95, GluN2A, and GluN2B, in *Cx3cr1*^Cre^CD31^fl/+^5×FAD mice compared with 5×FAD-WT controls (Fig. 1T, U). Dendritic spine density was also markedly elevated as assessed by Golgi staining (Fig. 1V).

Collectively, these findings indicate that microglia-specific knockdown of CD31 enhances synaptic integrity and cognitive function in 5×FAD mice.

### Microglial CD31 knockdown alleviates transcriptomic perturbations in 5×FAD mice at the single-nucleus level

To investigate the cellular and molecular alterations induced by microglial CD31 knockdown in 5×FAD mice, we performed single-nucleus RNA sequencing (snRNA-seq) on brain tissue from 6-month-old *Cx3cr1*^Cre^CD31^fl/+^5×FAD mice, 5×FAD-WT mice, and the littermate control mice (3 mice per group, totaling 9 mice). After removing doublets and filtering out low-quality cells, a final dataset of 104,908 high-quality nuclei was retained for downstream analysis. UMAP visualization revealed eight major brain cell populations: excitatory neurons (EX), inhibitory neurons (INH), astrocytes (Astro), oligodendrocytes (Oligo), oligodendrocyte precursor cells (OPC), vascular leptomeningeal cells (VLMC), smooth muscle cells/pericytes (SMC-Peri), and microglia/perivascular macrophages (Micro-PVM) (Supplementary Fig. 3A). Cell-type identities were validated by the expression of canonical marker genes, as shown in the dot plot (Supplementary Fig. 3B).

Quantification of cell type distribution revealed a significant expansion of Micro-PVM populations in 5×FAD-WT mice compared to normal Ctrl mice, whereas this increase was markedly attenuated by microglial-specific CD31 knockdown (Supplementary Fig. 3C, D). This finding is consistent with our earlier observation that CD31 knockdown led to a greater reduction in CD31 levels in the AD context, likely due to an elevated CD31 expression in microglia and a concomitant decrease in microglial abundance. Consistently, IBA1 immunofluorescence staining demonstrated a reduced density of microglia in *Cx3cr1*^Cre^CD31^fl/+^5×FAD mice (Supplementary Fig. 3E), indicating that CD31 knockdown alleviates microglial abnormal expansion observed in AD pathology. To clarify the basis underlying the reduced microglial abundance, we further assessed proliferation and apoptosis markers. While the number of TUNEL-positive microglia was unchanged, Ki67-positive microglia were significantly reduced following CD31 knockdown (Supplementary Fig. 4), indicating a restrained microglial proliferation rather than an increased cell loss.

To explore the transcriptomic impact of CD31 knockdown across major brain cell types, we performed pathway enrichment analysis of differentially expressed genes (DEGs) (Supplementary Data 3) across three comparisons: 5×FAD-WT vs. Ctrl, *Cx3cr1*^Cre^CD31^fl/+^5×FAD vs Ctrl, and *Cx3cr1*^Cre^CD31^fl/+^5×FAD vs 5×FAD-WT. Heatmap visualization revealed that multiple pathways dysregulated in 5×FAD-WT mice were reversed by CD31 knockdown (Supplementary Fig. 3F). Specifically, up-regulated DEGs in 5×FAD-WT mice compared to Ctrl mice were predominantly enriched in pro-inflammatory and microglia-related pathways, such as complement activation, microglia-mediated neuroinflammation, and the NLRP3 inflammasome pathway. Conversely, downregulated DEGs were primarily associated with synaptic signaling, calcium signaling, and long-term potentiation, which are critical for learning and memory. These perturbations were consistent with the hallmark inflammatory response and cognitive deficits observed in the AD brains.

Strikingly, when comparing *Cx3cr1*^Cre^CD31^fl/+^5×FAD to 5×FAD-WT mice, the pattern was reversed: up-regulated DEGs were enriched in synaptic and memory-related pathways, while down-regulated DEGs were mainly associated with inflammation and microglial activation. This transcriptomic reversal suggested that microglial CD31 knockdown effectively mitigated AD-related inflammatory responses and restored pathways critical for cognitive function. Notably, the pathway profile of CD31 knockdown mice showed minimal divergence from Ctrl mice, further supporting the protective and homeostatic effect of microglial CD31 reduction in the AD context.

To further validate these findings, we identified a set of 362 genes in Micro-PVM cells whose expression trends were reversed by CD31 knockdown in 5×FAD mice (Supplementary Fig. 5A and Supplementary Data 3). These genes were originally dysregulated in 5×FAD-WT mice relative to controls and showed opposite expression patterns in *Cx3cr1*^Cre^CD31^fl/+^5×FAD mice. Pseudo-bulk heatmap visualization of these reversed DEGs further demonstrated that many AD-upregulated genes were downregulated, and AD-downregulated genes were upregulated following CD31 knockdown (Supplementary Fig. 5B). These results provide transcript-level evidence that CD31 deletion effectively reverses AD-associated gene expression perturbations in microglia.

These findings together indicate that CD31 knockdown reshapes cell-type-specific transcriptomic profiles and attenuates AD-associated molecular perturbations in 5×FAD mice.

### Microglial-specific CD31 knockdown reprograms microglial states and attenuates pro-inflammatory activation in 5×FAD mice

**Fig. 2:**
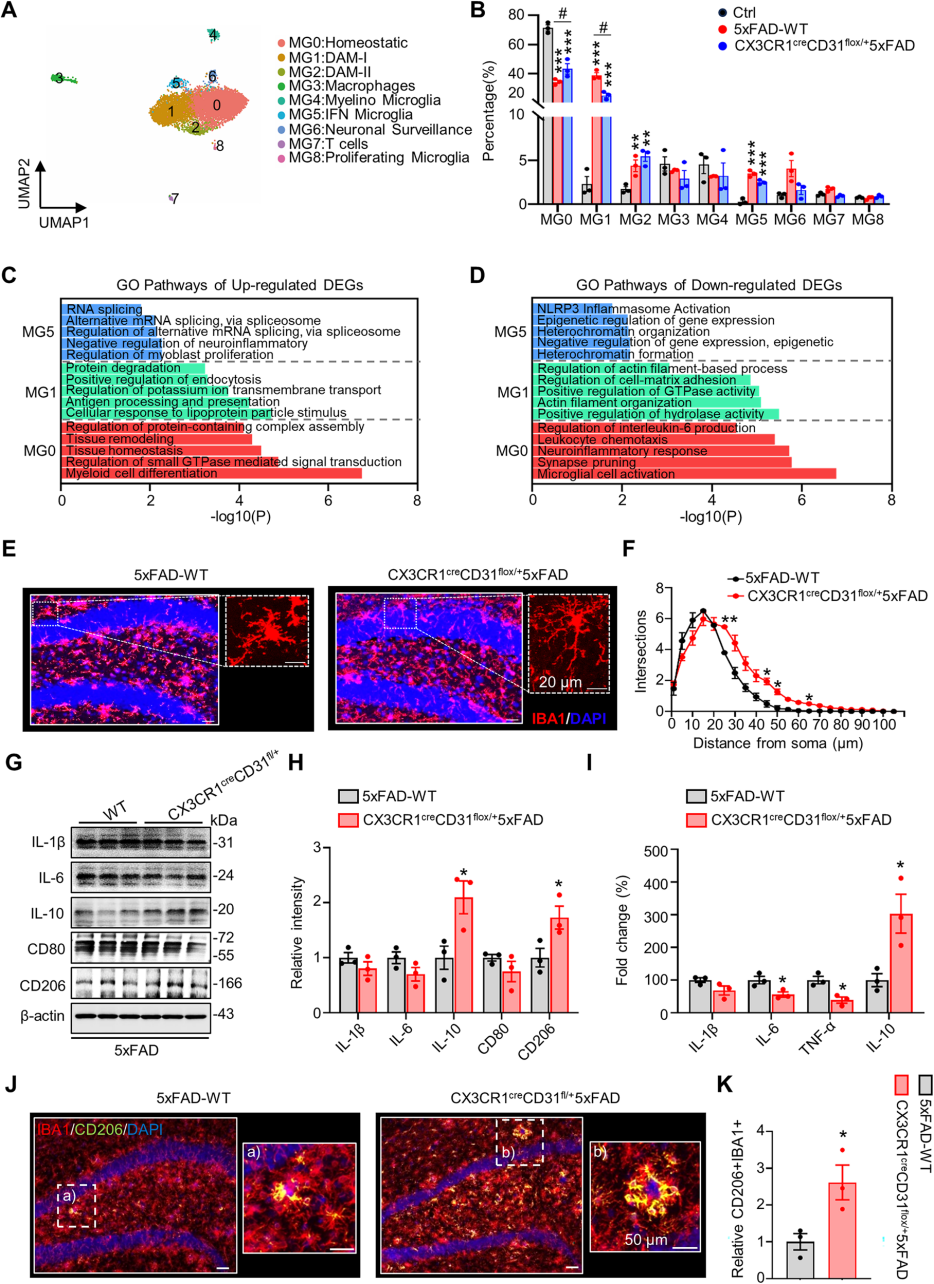
CD31 knockdown alleviates microglial inflammatory responses in 5×FAD mice. **A** UMAP visualization of Micro-PVM nuclei clustered into nine subpopulations based on transcriptional profiling from snRNA-seq data, with annotated functional states. **B** Bar plot showing the proportion of each Micro-PVM subcluster among Ctrl, 5×FAD-WT, and *Cx3cr1*^Cre^CD31^fl/+^5×FAD mice. CD31 knockdown reversed the reduction of MG0 subcluster and the increase of MG1 subcluster observed in the 5×FAD brain. *n* = 3 mice per group, differential abundance analysis (DAA) using the propeller/limma framework with Benjamini–Hochberg FDR correction, #*p* = 0.032 (MG0), #*p* = 0.018 (MG1) vs. 5×FAD-WT, **p < 0.01, ***p < 0.001 vs. Ctrl. (all *p* values FDR-adjusted, exact *p values provided in Supplementary Data 2). GO enrichment analysis of upregulated (**C**) and downregulated (**D**) DEGs in selected microglial subclusters (MG0, MG1, and MG5) comparing *Cx3cr1*^Cre^CD31^fl/+^5×FAD mice with 5×FAD-WT mice, revealing that CD31 knockdown promotes pathways related to protein degradation, tissue repair, and anti-inflammatory responses in microglia. Enrichment significance was assessed using a hypergeometric test without multiple comparison correction and is presented for exploratory purposes. **E**, **F** CD31 knockdown significantly enhanced microglial arborization in the hippocampus measured by immunofluorescence staining and Sholl analysis, *n* = 3 mice per group, two-way RM ANOVA with Šídák post hoc test, **p = 0.0031 (25 μm), *p = 0.0231 (45 μm), *p = 0.0428 (50 μm), and *p = 0.0328 (65 μm) vs. 5×FAD-WT. Bar = 20 μm. CD31 knockdown modulated protein and mRNA levels of the pro- (IL-1β, IL-6, and TNF-α) and anti-inflammatory (IL-10) cytokines, with an increased microglial transformation from pro- to anti-inflammatory (CD206-positive) phenotype in hippocampus measured by Western blotting (**G**, **H**), qPCR (**I**), and immunofluorescence co-staining (**J**, **K**). *n* = 3 mice per group, two-sided unpaired Student’s *t* test, *p = 0.0398 (IL-10 protein), *p = 0.0287 (CD206 protein), *p = 0.0338 (IL-6 mRNA), *p = 0.0148 (TNF-α mRNA), *p = 0.0320 (IL-10 mRNA) and *p = 0.0364 (CD206^+^IBA1^+^/IBA1^+^) vs. 5×FAD-WT. Bar = 50 μm. Data are presented as the mean ± SEM. Source data are provided as a Source data file.

To further characterize the states of microglia, we performed clustering analysis on 7571 Micro-PVM nuclei from the above snRNA-seq dataset and identified nine distinct clusters (Fig.2A). We defined these 9 clusters based on their molecular signatures and functions (Supplementary Fig. 6A, B, and Supplementary Data 4), and annotated homeostatic microglia (MG0) with high expression of well-known homeostatic markers *P2ry12* and *Cx3cr1* (Supplementary Fig. 6A). By further annotating disease-associated microglia (DAM) subtypes based on their shared expression of numerous typical DAM-related genes, we designated MG1 as DAM, and MG2 as metabolic DAM due to its high expression of mitochondrial genes (Supplementary Fig. 6A), suggesting a highly activated state with enhanced energy metabolism. The MG2 population was enriched in repair, phagocytosis, and degradation pathways (Supplementary Fig. 6B), indicating its strong capacity for pathological clearance. The MG3 was identified as CNS-associated macrophages based on their distinctive expression profile of macrophage-associated markers, such as *MRC1*, *CD163*, and *F13A1*^27^. The MG4 was annotated as myelin-associated microglia characterized by high expression of myelin-associated genes (Supplementary Fig. 6A), which were enriched in pathways related to axon ensheathment and glial migration (Supplementary Fig. 6B). The MG5 subcluster was designated as interferon-responsive microglia, characterized by upregulation of IFN-stimulated genes, such as *Trim30a* and *Rnf213* (Supplementary Fig. 6A) which were enriched in KEGG terms including interferon-mediated signaling pathway and activation of innate immune response (Supplementary Fig. 6B). The MG6 was annotated as neuronal surveillance microglia based on its strong enrichment in synapse-related pathways (Supplementary Fig. 6B). The MG7 was annotated as T cells based on the enrichment of T cells associated signaling pathway (Supplementary Fig. 6B). The MG8 was annotated as proliferating microglia based on the strongest enrichment cell cycle and mitosis-related KEGG terms (Supplementary Fig. 6B). Further differential abundance analysis revealed that microglia-specific CD31 knockdown selectively altered the distribution of microglial subclusters in the 5×FAD brain (Fig. 2B). CD31-deficient 5×FAD mice showed a significant increase in the MG0 cluster accompanied by a significant decrease in the MG1 cluster compared with 5×FAD controls, indicating a partial rebalancing of the microglial landscape in the 5×FAD brain.

Based on these abundance changes, we next examined transcriptional alterations within representative microglial subpopulations. Subsequent analyses focused on MG0 and MG1, which showed the most pronounced abundance shifts, and additionally included MG5, a transcriptionally distinct activated microglial population relevant to AD pathology. We therefore performed differential gene expression and GO enrichment analyses within the MG0, MG1, and MG5 microglial subclusters (Fig.2C, D, and Supplementary Data 3). In the MG0 homeostatic microglia subcluster, CD31 knockdown was associated with transcriptional programs indicative of an enhanced homeostatic maintenance and cellular stability, accompanied by reduced expression of genes linked to inflammatory signaling and synapse pruning. These features are consistent with a transcriptional bias toward a more homeostatic microglial profile. In the MG1 (DAM) subcluster, CD31 knockdown was associated with transcriptional programs indicative of an enhanced cellular clearance and degradative functions, accompanied by reduced expression of genes linked to cytoskeletal remodeling and activation-associated processes. These features are consistent with a transcriptional bias toward lower inflammatory activation and enrichment of repair-associated programs within DAM microglia. In the MG5 (IFN) microglial subcluster, CD31 knockdown was associated with transcriptional programs related to altered RNA processing and reduced interferon-driven inflammatory signaling. These features were accompanied by decreased expression of genes linked to inflammasome activation and chromatin-associated regulatory processes, indicating attenuation of IFN-associated inflammatory signatures. These changes are consistent with a transcriptional bias toward reduced inflammatory activation and enrichment of repair-associated programs within IFN-responsive microglia.

Violin plots further illustrated genotype-associated differences in the expression of representative functional genes across microglial subclusters (Supplementary Fig. 6C–E). In the homeostatic MG0 subcluster, the expression of homeostatic markers, such as *Tmem119*, *P2ry12*, and *Cx3cr1* was markedly reduced in 5×FAD-WT mice, but significantly restored upon CD31 deletion, suggesting enhanced maintenance of microglial homeostasis (Supplementary Fig. 6C). In the DAM subclusters MG1, genes related to degradation and tissue repair, such as *Cst7*, *Cd9*, and *Ctsd*, were upregulated following CD31 knockdown, highlighting its role in promoting microglial clearance and reparative functions (Supplementary Fig. 6D). Moreover, in the interferon-responsive MG5 subcluster, pro-inflammatory genes including *Ifit2*, *Oasl2*, *Irf7*, and *Ifi209* were highly expressed in 5×FAD-WT mice, while CD31 knockdown significantly suppressed their expression, indicating attenuation of IFN-driven inflammatory activation (Supplementary Fig. 6E).

Together, these single-nucleus transcriptomic findings indicate that CD31 knockdown is associated with subtype-specific transcriptional biases in microglia, characterized by reduced inflammatory signaling and the enhanced homeostatic, clearance-, and repair-associated programs.

To further validate the anti-inflammatory and homeostatic trends revealed by the single-nucleus transcriptomic analysis, we first examined microglial morphology. In *Cx3cr1*^Cre^CD31^fl/+^5×FAD mice, microglia exhibited fewer proximal processes and greater total process length compared to those in 5×FAD-WT mice (Fig. 2E, F), indicating a more quiescent and less reactive state. These morphological changes were consistent with the transcriptomic findings of enhanced homeostatic features.

We next examined the expression of inflammatory markers in the hippocampus. Compared with 5×FAD-WT mice, hippocampal tissues from CD31-knockdown mice showed significantly increased levels of IL-10 and CD206, accompanied by a decreasing trend in pro-inflammatory markers IL-1β, IL-6, and CD80 (Fig. 2G, H). qPCR analysis further confirmed a significant upregulation of IL-10 and downregulation of IL-6 and TNF-α (Fig. 2I). Immunofluorescence staining revealed a significantly increased number of CD206-positive microglial cells in the hippocampus of *Cx3cr1*^Cre^CD31^fl/+^5×FAD mice (Fig. 2J, K), indicating that CD31 knockdown promotes a shift in microglial phenotype toward an anti-inflammatory and potentially tissue-reparative state.

These findings collectively demonstrate that microglia-specific CD31 knockdown restores homeostatic balance and reprograms DAM states toward anti-inflammatory, reparative, and degradative phenotypes in 5×FAD mice.

### Microglia-specific knockdown of CD31 reduces brain Aβ levels in 5×FAD mice

**Fig. 3:**
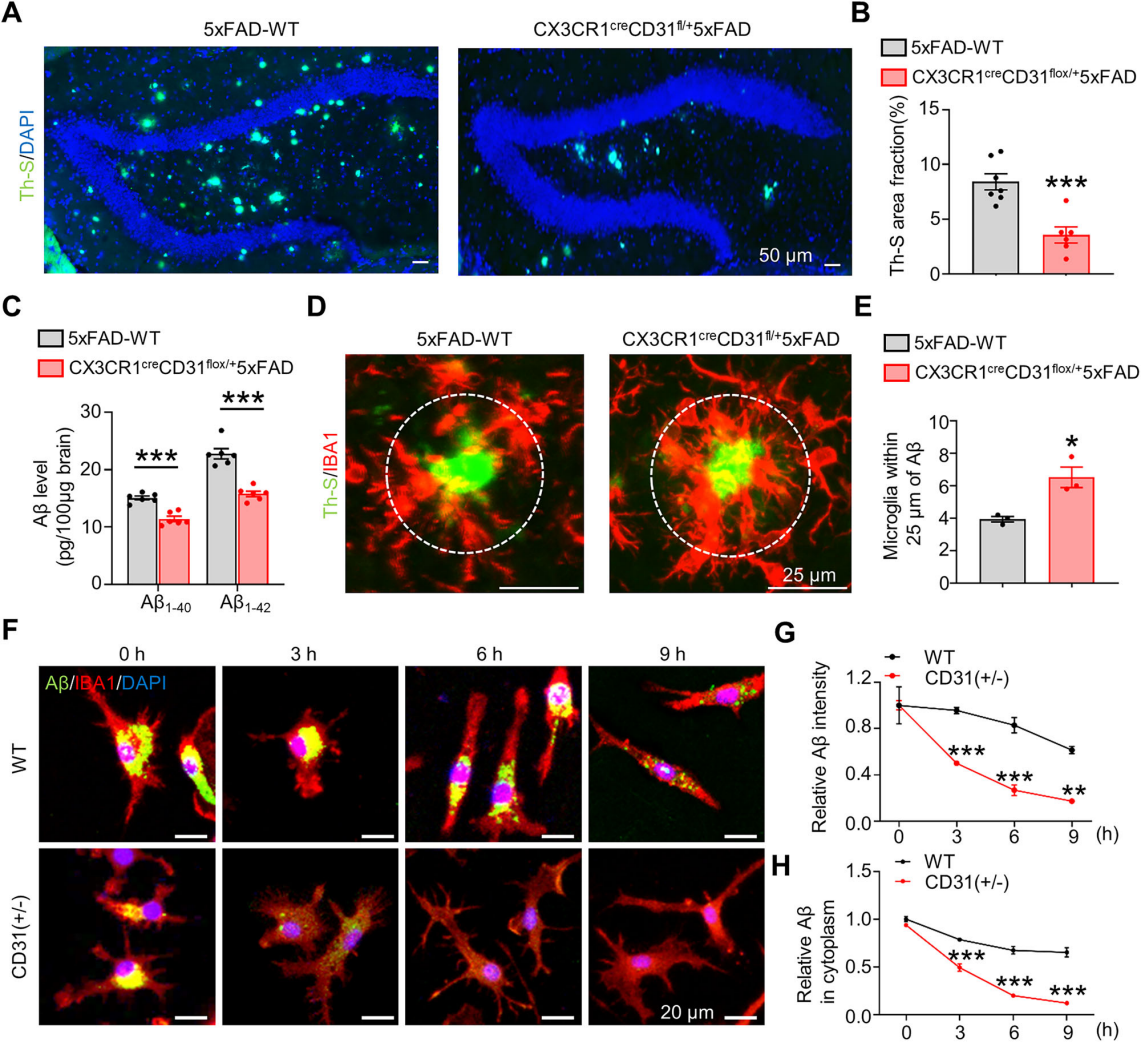
CD31 regulates microglial degradation of Aβ in 5×FAD mice. **A**, **B** CD31 knockdown significantly decreased hippocampal Aβ plaque content measured by thioflavin-S (Th-S) staining. *n* = 6 (*Cx3cr1*^Cre^CD31^fl/+^5×FAD) and 7 (5×FAD-WT) mice, two-sided unpaired Student’s *t* test, ***p = 0.0007 vs. 5×FAD-WT. Bar = 50 μm. **C** CD31 knockdown significantly decreased hippocampal Aβ_1–40_ and Aβ_1–42_ levels. *n* = 6 mice per group, two-sided unpaired Student’s *t* test, ***p < 0.0001 vs. 5×FAD-WT. **D**, **E** CD31 knockdown significantly promoted microglia migration to the Aβ plaques, evidenced by an increased number of IBA1^+^ microglia (red) within 25 μm of Th-S⁺ Aβ plaques (green). *n* = 3 mice per group, 5 plaques were analyzed per mouse, two-sided unpaired Student’s *t* test, *p = 0.0170 vs. 5×FAD-WT. Bar = 25 μm. CD31 knockdown enhanced the clearance of fluorescently labeled intracellular Aβ in cultured primary microglia, as shown by time-dependent reduction in intracellular Aβ levels detected by fluorescence staining (**F**, **G**) and ELISA (**H**). *n* = 3 independent experiments, two-way ANOVA with Šídák post hoc test, ***p = 0.0008 (3 h), ***p < 0.0001 (6 h), and **p = 0.0011 (9 h) vs. WT for (**G**) and ***p < 0.0001 (3, 6, and 9 h) vs. WT for (**H**). Bar = 20 μm. Data were presented as mean ± SEM. Source data are provided as a Source data file.

Microglial functionality is closely related to the clearance of Aβ in the brain. To investigate whether microglia-specific knockdown of CD31 similarly affects Aβ, we first assessed the Aβ plaque burden. A significant reduction in hippocampal Aβ plaque burden with reduced levels of Aβ_1–40_ and Aβ_1–42_ and increased numbers of microglial cells around the plaques was detected in *Cx3cr1*^Cre^CD31^fl/+^5×FAD mice compared to 5×FAD-WT (Fig. 3A–E). These results reveal that microglia-specific knockdown of CD31 not only decreases brain Aβ deposition but also enhances microglial engagement around plaques, indicating improved microglial recruitment and plaque surveillance.

To explore how microglia-specific CD31 knockdown reduces Aβ level in 5×FAD mice, we cultured primary microglia from wild-type and CD31 knockdown mice and treated the cells with fluorophore-labeled Aβ_1–42_ oligomers (5 μM). After a 2-h incubation to allow Aβ uptake, extracellular Aβ was thoroughly removed by PBS washing. The fluorescence intensity measured immediately after washing (defined as time zero) therefore reflects the amount of internalized Aβ and serves as an indicator of microglial phagocytic uptake. Degradation of ingested Aβ was monitored over 9 h. Immunofluorescence at the initial time point showed substantial Aβ presence in both wild-type and CD31 knockdown microglia with no significant differences, indicating that CD31 knockdown did not affect microglial phagocytic capacity (Fig. 3F, G). In contrast, the progressive decline in intracellular fluorescence over time reflects intracellular processing and degradation of the internalized Aβ. Notably, Aβ was almost completely degraded at 9 h in CD31 knockdown microglia, at a markedly faster rate than in wild-type cells (Fig. 3F, G). The significantly enhanced Aβ degradation without change of phagocytosis in CD31 knockdown microglia was also detected by ELISA (Fig. 3H). These findings suggest that microglia CD31 knockdown reduces Aβ level and plaque deposition by increasing Aβ degradation but not Aβ phagocytosis.

### Microglia-specific knockdown of CD31 enhances Aβ clearance through increasing transcription of MME

**Fig. 4:**
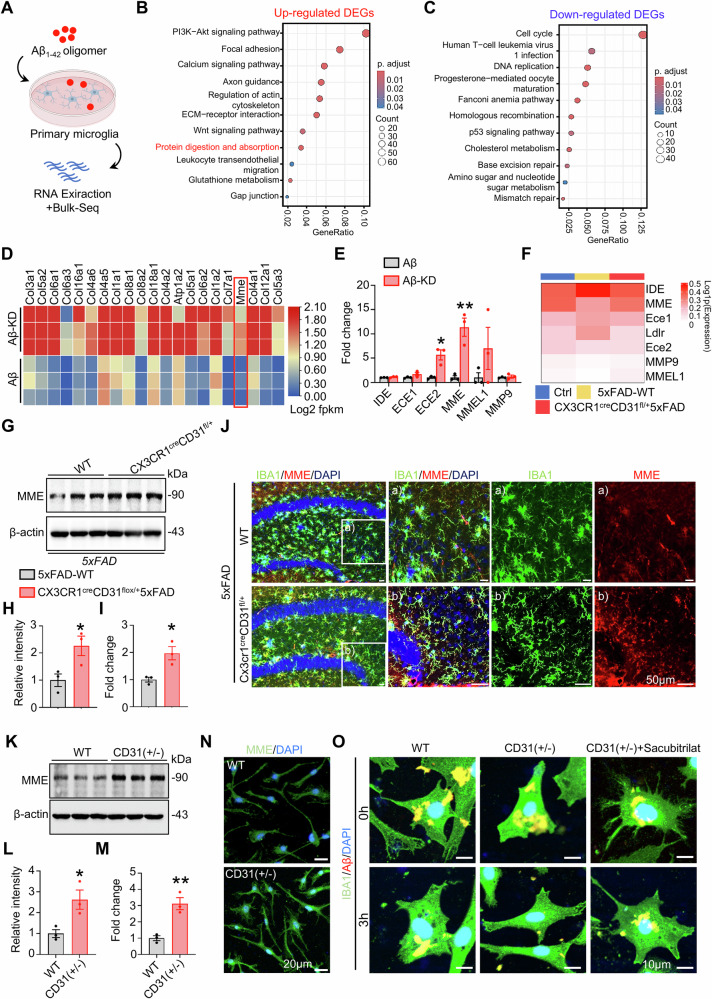
CD31 regulates Aβ clearance by modulating MME transcription. **A** Schematic depecting the experimental design for transcriptomic analysis of primary microglia treated with Aβ_1–42_ oligomers with or without CD31 knockdown. Certain illustrative components are created by Figdraw (ID: ISIWTc707c). **B**, **C** KEGG pathway enrichment analysis of DEGs between CD31-knockdown and wild-type primary microglia. Upregulated genes were enriched in PI3K−Akt signaling, ECM−receptor interaction, Wnt signaling, and protein digestion and absorption (**B**), while downregulated genes were associated with immune/inflammatory pathways, such as DNA replication, cell cycle, and cholesterol metabolism (**C**). KEGG enrichment significance was assessed by the hypergeometric test with Benjamini–Hochberg adjustment for multiple comparisons. **D** Heatmap showing upregulation of protein digestion/absorption pathway genes in CD31-knockdown microglia, including the Aβ-degrading enzyme MME. **E** Transcriptomic analysis of Aβ-treated microglia revealed significant upregulation of MME among Aβ-degrading enzymes in CD31-knockdown cells. *n* = 3 mice per group, two-sided unpaired Student’s *t* test, *p = 0.0114 (ECE2), **p = 0.0058 (MME) vs. WT. **F** Heatmap displaying expression of Aβ-degrading enzymes in microglial subpopulations from Ctrl, 5×FAD-WT, and *Cx3cr1*^Cre^CD31^fl/+^5×FAD mice. CD31 knockdown reverses 5×FAD-induced MME downregulation. CD31 knockdown increased hippocampal MME protein (**G**, **H**, **J**) and mRNA (**I**) levels in 5×FAD mice, as detected by Western blotting, immunofluorescence, and qPCR. *n* = 3 mice per group, two-sided unpaired Student’s *t* test, *p = 0.0421 (**H**) and *p = 0.0204 (**I**) vs. 5×FAD-WT. Bar = 50 μm. CD31 knockdown increased MME protein (**K**, **L**, **N**) and mRNA (**M**) levels in cultured primary microglia. *n* = 3 mice per group, two-sided unpaired Student’s *t* test, *p = 0.0301 (**L**) and **p = 0.0060 (**M**) vs. 5×FAD-WT. Bar = 20 μm. **O** The MME inhibitor Sacubitrilat blocked CD31 knockdown-promoted Aβ degradation in primary microglia. Representative immunofluorescence images from three independent experiments with similar results. Bar = 10 μm. Data were presented as mean ± SEM. Source data are provided as a Source data file.

To investigate the mechanism by which CD31 knockdown enhances Aβ clearance, we performed transcriptomic analysis on primary microglia isolated from wild-type and microglia CD31 knockdown mice after 24-h treatment with oligomeric Aβ (Fig. 4A). Transcriptomic analysis identified 2058 differentially expressed genes, with 1370 DEGs upregulated and 688 DEGs downregulated in CD31-knockdown microglia. KEGG pathway analysis revealed upregulation of genes involved in pathways, such as PI3K−Akt signaling, ECM−receptor interaction, Wnt signaling, and protein digestion and absorption (Fig. 4B), while downregulated genes were enriched in pathways related to DNA replication, cell cycle, and cholesterol metabolism (Fig. 4C). Notably, macrophage metalloelastase (MME) transcription in the protein digestion and degradation pathway was significantly elevated (Fig. 4D and Supplementary Data 3). Since Aβ degradation is primarily mediated by Aβ-degrading enzymes, we hypothesized that CD31 may influence this process by modulating their expression. Further analysis confirmed increased transcription of Aβ-degrading enzymes, including MME and endothelin-converting enzymes 2 (ECE2), with MME showing the most pronounced elevation (Fig. 4E). Consistently, heatmap analysis of microglial subpopulations from Ctrl, 5×FAD-WT, and *Cx3cr1*^Cre^CD31^fl/+^5×FAD mice revealed that CD31 knockdown reversed the 5×FAD-induced downregulation of MME expression (Fig. 4F and Supplementary Data 3). Further studies revealed that both protein and mRNA levels of MME were significantly elevated in both *Cx3cr1*^Cre^CD31^fl/+^5×FAD mice (Fig. 4G–J) and primary microglia with CD31 knockdown (Fig. 4K–N). Moreover, the enhanced Aβ degradation induced by CD31 knockdown was reversed by treatment with the MME inhibitor Sacubitrilat (5 nM) (Fig. 4O), confirming the critical role of MME in this process.

Collectively, both in vitro and in vivo experiments at the transcriptional and translational levels confirmed that increasing CD31 in microglia led to reduced MME expression, whereas knocking down CD31 restored MME levels that resulted in an enhanced microglial Aβ degradation. Furthermore, inhibiting MME activity reversed the enhanced Aβ degradation by CD31 knockdown, which underscored the pivotal role of MME in this process.

### Microglial-specific knockdown of CD31 activates STAT3 to enhance MME transcription and promote Aβ clearance

**Fig. 5:**
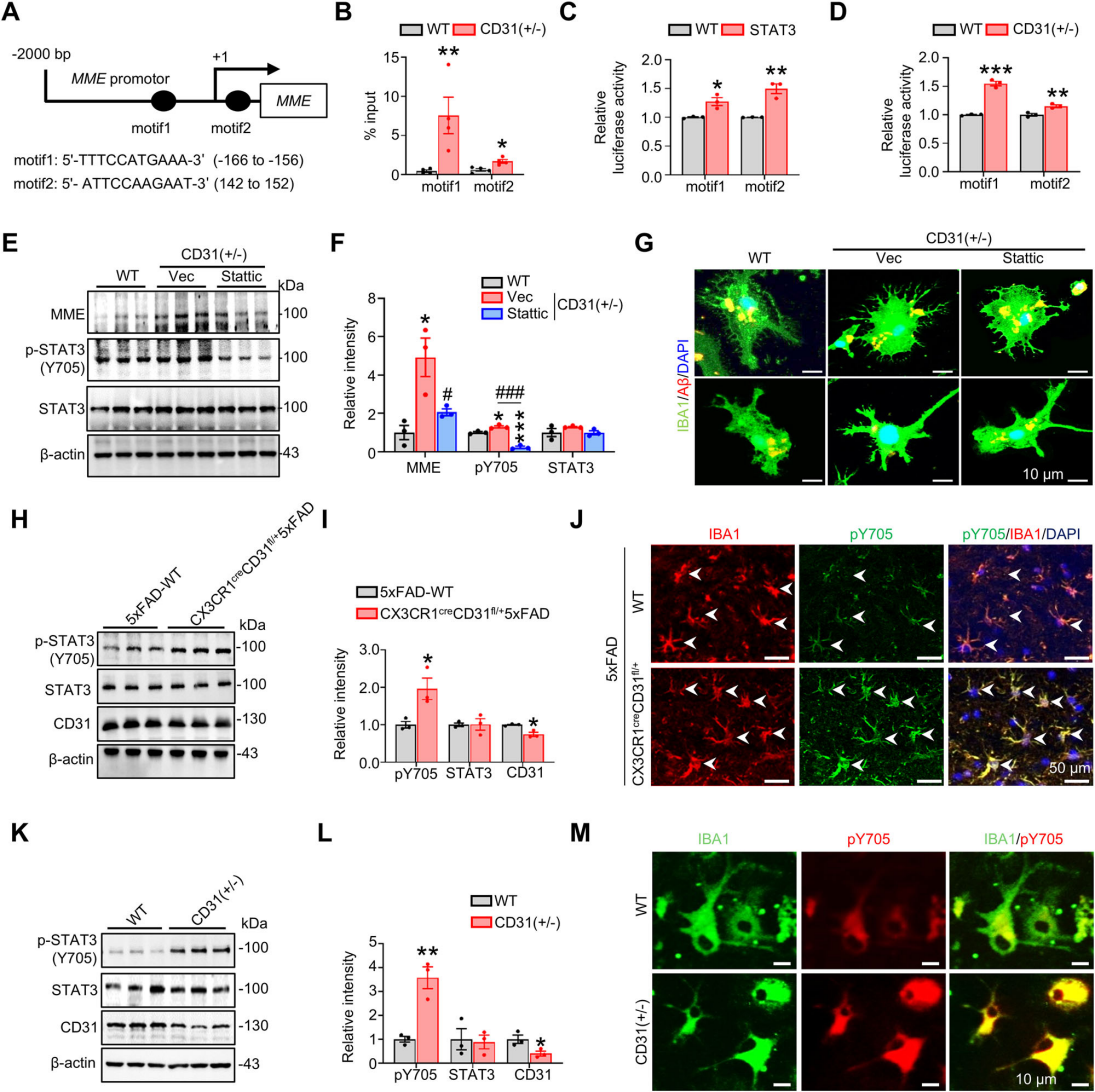
Microglial-specific CD31 knockdown promotes Aβ clearance via activation of STAT3-MME signaling. **A** Predicted STAT3 binding motifs in the MME gene promoter were identified using the JASPAR database. **B** CHIP assay showing enhanced binding of STAT3 to both predicted MME promoter motifs in CD31-knockdown primary microglia vs WT. *n* = 4 independent experiments, two-sided unpaired Student’s *t* test, *p = 0.0131 (motif1), **p = 0.0088 (motif2) vs. WT. **C** Luciferase reporter assay in HEK293 cells: Co-expression of STAT3 plasmids with luciferase reporters containing MME promoter motifs drove STAT3-dependent MME transcriptional activity. *n* = 3 independent experiments, two-sided unpaired Student’s *t* test, *p = 0.0479 (motif1), **p = 0.0012 (motif2) vs. WT. **D** Luciferase reporter assay in primary microglia (wild-type vs. CD31-knockdown) using lentiviruses carrying MME promoter-driven luciferase: CD31 knockdown enhanced MME transcriptional activity. *n* = 3 independent experiments, two-sided unpaired Student’s *t* test, **p = 0. 0095 (motif2), ***p = 0.0002 (motif1) vs. WT. The STAT3 inhibitor Stattic (20 μM, 24 h) abolished CD31 knockdown-induced MME upregulation (**E**, **F**) and enhanced Aβ degradation (**G**) in primary microglia detected by Western blotting (**E**, **F**) and immunofluorescence staining (**G**). *n* = 3 independent experiments, one-way ANOVA with Tukey post hoc test, *p = 0.0107 for WT vs. CD31(±)-Vec (MME), *p = 0.0210 for WT vs CD31(±)-Vec (pY705), ***p < 0.0001 for WT vs. CD31(±) + Sattic (pY705); #*p* = 0.0418 for CD31(±)-Vec vs. CD31(±) + Sattic (MME), ###*p* < 0.0001 for CD31(±)-Vec vs. CD31(±) + Sattic (pY705). Bar = 10 μm. **H**, **I** Microglia-specific CD31 knockdown significantly increased STAT3 phosphorylation (pSTAT3) in the hippocampus of 5×FAD mice detected by Western blotting. *n* = 3 mice per group, two-sided unpaired Student’s *t* test, *p = 0.0105 (CD31) and *p = 0.0331 (pY705) vs. 5×FAD-WT. **J** Representative immunofluorescence images from three mice per group with similar results show increased microglial p-STAT3 after CD31 knockdown. Bar = 50 μm. CD31 knockdown enhance STAT3 phosphorylation in primary microglia detected by Western blotting (**K**, **L**) and immunofluorescence (**M**). *n* = 3 independent experiments, two-sided unpaired Student’s *t* test, *p = 0.0407 (CD31), **p = 0.0055 (pY705) vs. WT. Bar = 10 μm. Data were presented as mean ± SEM. Source data are provided as a Source datafile.

As shown above, microglial CD31 knockdown increased MME transcription, suggesting the involvement of transcription factors influenced by CD31. Single-nucleus transcriptomic analysis revealed that CD31 knockdown altered the JAK-STAT signaling pathway (Supplementary Fig. 3F), suggesting its potential involvement in transcriptional regulation downstream of CD31. Signal transducer and activator of transcription 3 (STAT3), a key transcription factor in AD progression, plays critical roles in synaptic function, microglial activity, and anti-inflammatory responses. Notably, our previous study indicates a reduction in STAT3 phosphorylation, influencing both synaptic damage and cognitive deficits^28^. Utilizing CHIP-Seq data from the hTFtarget database, we identified potential STAT3 binding sites on the MME promoter, indicating that STAT3 regulates MME transcription. We speculated that CD31 might influence MME transcription through STAT3. To clarify this, we analyzed MME gene promoter sequences for potential STAT3 binding DNA sequences via the JASPAR database. Two probable STAT3 binding motifs were identified: 5′-TTTCCATGAAA-3′ (motif1) and 5′-ATTCCAAGAAT-3′ (motif2) (Fig. 5A). ChIP-qPCR experiments on normal and CD31-knocked down microglia showed that STAT3 directly targets these motifs, with CD31 knockdown significantly enhancing this binding (Fig. 5B). Luciferase reporter assays in HEK293 cells and in normal or CD31-knocked down primary cultured microglia demonstrated that overexpressing STAT3 or knocking down CD31 significantly promoted MME transcription (Fig. 5C, D).

To further confirm the necessity of STAT3 activation in the enhanced Aβ degradation resulting from microglial-specific CD31 knockdown, we treated CD31-knocked down microglia with the STAT3 inhibitor Stattic (20 μM). Stattic treatment reversed the increase in MME expression caused by CD31 knockdown, and reduced phosphorylation at Tyr705 of STAT3 in primary cultured microglia (Fig. 5E, F). Immunofluorescent staining data also demonstrated that Stattic treatment inhibited the enhanced Aβ degradation in CD31-knocked-down microglia (Fig. 5G).

To confirm that CD31 knockdown can indeed lead to STAT3 activation, we next examined its phosphorylation status, as phosphorylation at Tyr705 is a well-established marker of STAT3 activation. Firstly, we examined total STAT3 levels and its phosphorylation at Tyr705 in the hippocampus of 5×FAD and *Cx3cr1*^Cre^CD31^fl/+^5×FAD mice. Significantly increased levels of phosphorylated STAT3 were observed in *Cx3cr1*^Cre^CD31^fl/+^5×FAD mice compared to 5×FAD-WT mice (Fig. 5H, I). Elevated phosphorylated STAT3 levels in microglia from *Cx3cr1*^Cre^CD31^fl/+^5×FAD mice were confirmed via immunofluorescent staining, indicating enhanced transcriptional activity of STAT3 due to CD31 knockdown (Fig. 5J). We also assessed total STAT3 and its phosphorylated forms in normal and CD31-knocked down primary cultured microglia. Phosphorylated forms of STAT3 were significantly elevated in CD31-knocked down microglia (Fig. 5K–M).

These data demonstrate that microglial-specific knockdown of CD31 activates STAT3 by increasing its phosphorylation, which in turn promotes MME transcription and ultimately enhances microglial clearance of Aβ.

### Microglial CD31 modulates STAT3 phosphorylation through interaction with STAT3 and SHP2

**Fig. 6:**
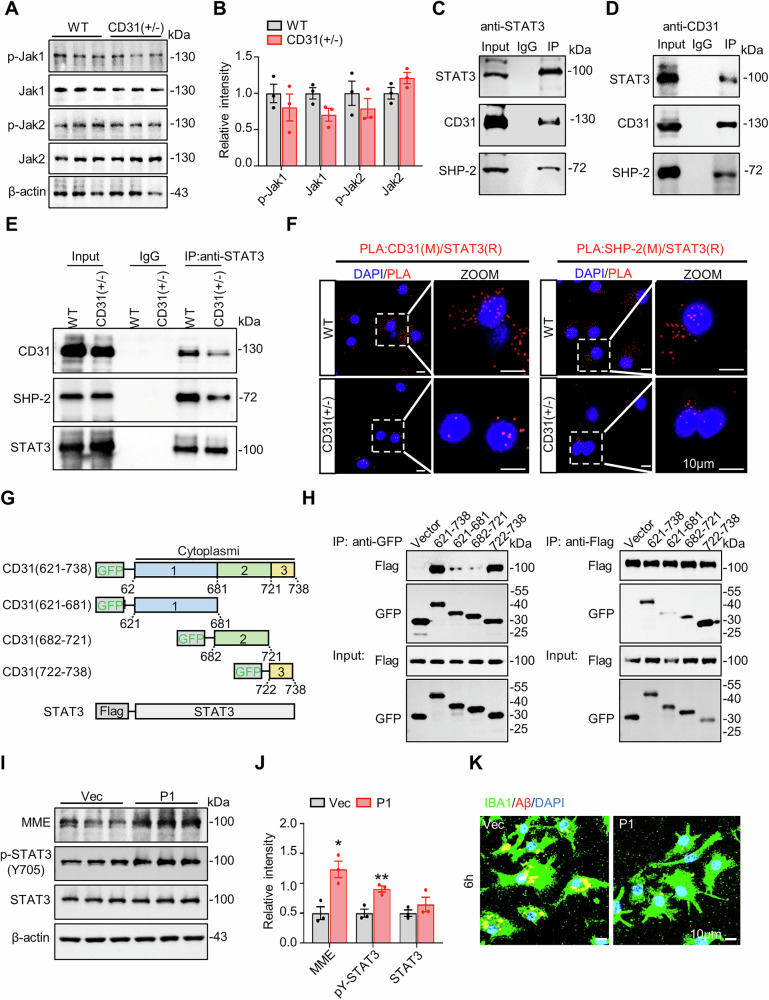
CD31 modulates STAT3 phosphorylation by linking SHP-2 to STAT3. **A**, **B** Microglia-specific CD31 knockdown did not significantly alter JAK expression and phosphorylation levels, as detected by Western blotting. *n* = 3 mice per group, two-sided unpaired Student’s *t* test; *p* = 0.4335 (p-JAK1), *p* = 0.0675 (JAK1), *p* = 0.3895 (p-JAK2), *p* = 0.1267 (JAK2) vs. WT. Co-immunoprecipitation (Co-IP) assays using anti-STAT3 (**C**) or anti-CD31 (**D**) antibodies, followed by Western blotting with anti-CD31, anti-STAT3, and anti-SHP-2, confirmed associations among CD31, STAT3, and SHP-2 in primary microglia. Representative blots from three independent experiments with similar results are shown. **E** Microglial CD31 knockdown reduced the interactions between STAT3 and CD31, as well as STAT3 and SHP-2, as shown by Co-IP and Western blotting. Representative blots from three independent experiments with similar results are shown. **F** Proximity ligation assay (PLA) revealed decreased interactions among STAT3, CD31, and SHP-2 in CD31-knockdown primary microglia. Representative images from three independent experiments with similar results are shown. Bar = 10 μm. **G** Schematic of GFP-tagged plasmids encoding full-length CD31, its intracellular domain and Flag-tagged STAT3 plasmid. **H** All three segments of the CD31 intracellular domain bound STAT3 in HEK293 cells co-transfected with GFP-tagged CD31 intracellular segment plasmids (or GFP empty vector) and Flag-STAT3 (or Flag empty vector), with the 722–738 segment identified as the primary binding site. Co-IP and Western blotting were performed 48 h post-transfection. Representative blots from three independent experiments with similar results are shown. **I**, **J** Treatment of primary cultured microglia with 100 μM peptide P1 for 24 h significantly increased STAT3 phosphorylation and MME expression. *n* = 3 independent experiments, two-sided unpaired Student’s t-test, *p = 0.0135 (MME), **p = 0.0088 (pY-STAT3) vs. Vec. **K** Representative immunofluorescence images from three independent experiments with similar results demonstrated enhanced Aβ degradation by primary microglia after 24 h treatment with P1 (100 μM). Bar = 10 μm. Data were presented as mean ± SEM. Source data are provided as a Source data file.

Given that knocking down CD31 activated STAT3 by increasing p-STAT3(Y705) levels, we asked how did CD31 regulate STAT3 phosphorylation? To answer this question, we first analyzed the levels and phosphorylation status of JAK1 and JAK2, the primary kinases regulating STAT3, in both wild-type and CD31-knockdown primary cultured microglia. No differences in total and phosphorylated levels of JAK1/JAK2 were observed between primary microglia with and without CD31 knockdown (Fig. 6A, B), indicating that CD31 knockdown did not affect JAK expression or activity, nor did it promote STAT3 phosphorylation through JAK. Src homology 2 protein tyrosine phosphatase-2 (SHP2), a non-receptor protein tyrosine phosphatase, has been extensively documented to regulate STAT3 phosphorylation levels. Moreover, CD31 has been reported to form a ternary complex with SHP2 and β-catenin, facilitating the dephosphorylation of β-catenin by SHP2. Based on these studies, we hypothesized that CD31 may promote the dephosphorylation of STAT3 by SHP2 through direct interaction, and that CD31 knockdown could reverse this effect, thereby increasing STAT3 phosphorylation. To test this hypothesis, we investigated the interactions among CD31, STAT3, and SHP2 in primary microglia using co-immunoprecipitation (Co-IP) assays. The results showed these proteins interact irrespective of the antibody used for enrichment (Fig. 6C, D). Moreover, CD31 knockdown substantially diminished the binding between STAT3, CD31, and SHP2 (Fig. 6E). Proximity ligation assays further demonstrated the decreased interactions between STAT3 and CD31/SHP2 in microglia with CD31 knockdown (Fig. 6F).

To further investigate the role of SHP-2 in mediating the increase of STAT3 phosphorylation induced by CD31 knockdown, we first examined the expression and phosphorylation status of SHP-2 in primary microglia. Western blot analysis showed that CD31 knockdown did not alter total SHP-2 protein levels or its active-site phosphorylation (Supplementary Fig. 7A, B), indicating that the enhanced STAT3 phosphorylation was not attributable to changes in SHP-2 abundance or catalytic activation. We next assessed whether pharmacological inhibition of SHP-2 could mimic the effect of CD31 knockdown. Treatment of BV2 cells with the SHP-2 inhibitor SHP099 (0.07 μM, 24 h) significantly increased MME expression and STAT3 phosphorylation levels (Supplementary Fig. 7C, D). Consistently, SHP-2 inhibition enhanced Aβ degradation in BV2 cells, as determined by immunofluorescence analysis (Supplementary Fig. 7E). Collectively, these findings indicate that CD31 knockdown regulates STAT3 phosphorylation in a SHP-2-dependent manner, thereby promoting MME expression and enhancing microglial Aβ degradation.

To investigate the specific binding sites of CD31 with STAT3, we constructed plasmids encoding full-length and various intracellular fragments of CD31 (621–681, 682–721, and 722–738) tagged with GFP, along with plasmids encoding Flag-tagged STAT3 (Fig. 6G). In HEK293 cells, GFP-tagged intracellular CD31 fragments (621–681, 682–721, 722–738) and a Flag-tagged STAT3 were transiently co-transfected. Co-immunoprecipitation results showed binding between STAT3 and all three CD31 segments, with the 722–738 fragment demonstrating the most significant interaction, identifying it as the primary binding site (Fig.6H). The findings suggest that disrupting the interaction between CD31 and STAT3 can enhance STAT3 phosphorylation and thereby promote microglial clearance of Aβ. Based on this concept, a peptide derived from the N-terminal of CD31 segment (722–738) and tagged with a transmembrane sequence termed as P1 (RRRRRRRRDAVESRYSRTEGSLDGT) was synthesized to disrupt CD31-STAT3 binding. Different concentrations of P1 were applied to BV-2 cells, and no significant cytotoxicity was observed (Supplementary Fig. 8A). P1 treatment significantly increased p-STAT3(Y705) expression in a concentration-dependent manner (Supplementary Fig. 8B, C). Co-IP confirmed that treatment with 100 µM P1 for 24 h effectively reduced the binding between CD31 and STAT3 (Supplementary Fig. 8D). Proximity ligation assays further validated the reduced interaction (Supplementary Fig. 8E), and immunofluorescence assays showed that P1 treatment significantly enhanced Aβ degradation in BV-2 cells (Supplementary Fig. 8F). Similar results were obtained when treating primary microglia with 100 µM P1 (Fig. 6I–K).

These data confirm that CD31 interacts with STAT3 and SHP2, and that CD31 knockdown decreases their binding, thereby increasing STAT3 phosphorylation and enhancing microglial Aβ clearance.

### Intracerebroventricular administration of P1 improves cognitive function and reduces Aβ pathology in 5×FAD mice

**Fig. 7:**
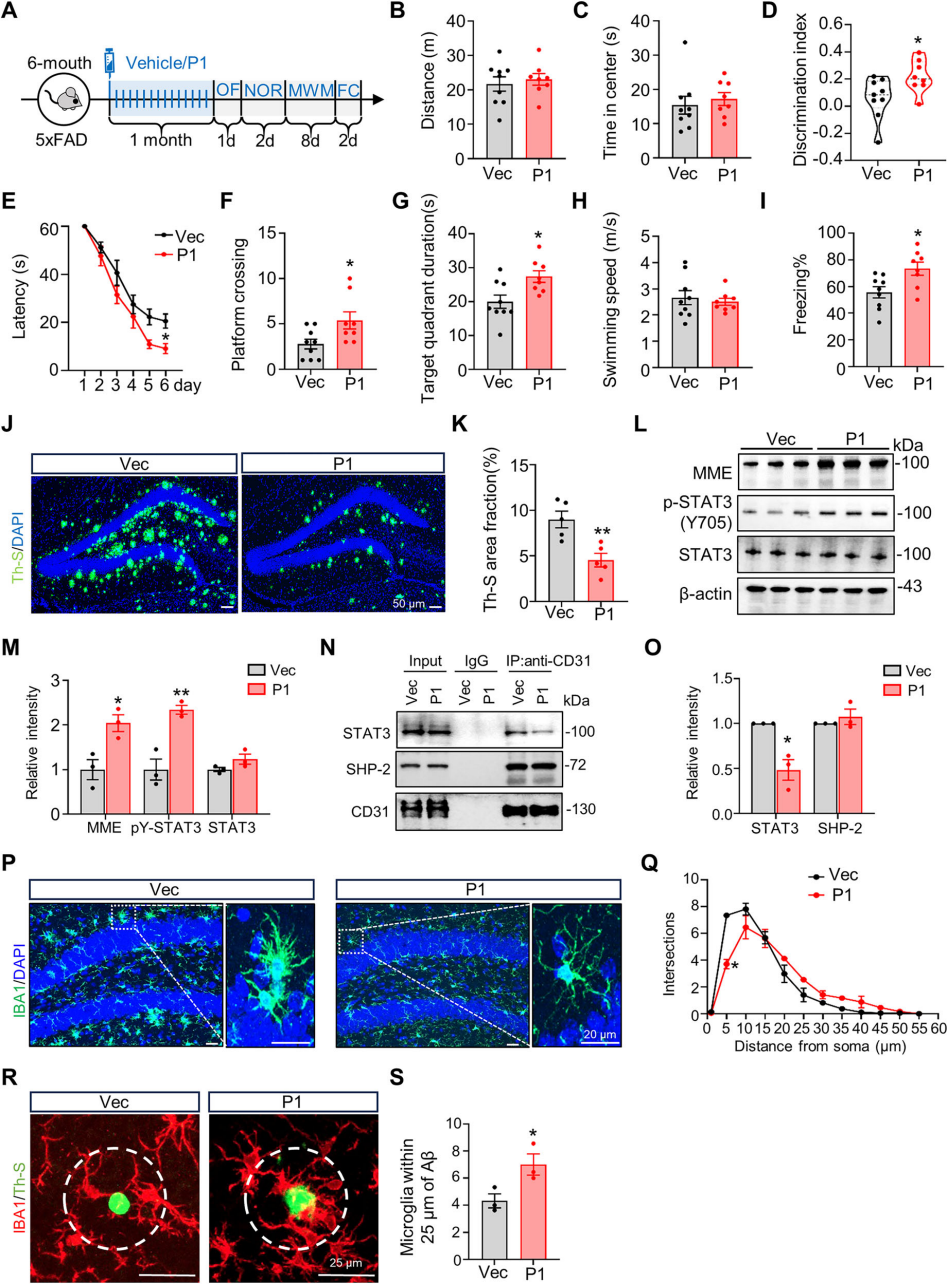
Intracerebroventricular injection of peptide P1 improves cognitive function and reduces Aβ pathology in 5×FAD mice. **A** Experimental scheme for ICV P1 administration in 6-month-old 5×FAD mice and the subsequent analyses. **B**–**I** Behavioral assessments. No effect on total distance (**B**) or center time (**C**) in OF, two-sided unpaired Student’s *t* test. Increased discrimination index **D** in NOR, two-sided unpaired Student’s *t* test, *p = 0.0318 vs. Vec. In MWM, reduced escape latency during training (**E**), two-way RM ANOVA with Šídák post hoc test, *p = 0.0409 (day 6) vs. Vec; improved probe performance with increased platform crossings (**F**) and target quadrant time (**G**), without change in swimming speed (**H**), two-sided unpaired Student’s *t* test, *p = 0.0271 (**F**), *p = 0.0133 (**G**) vs. Vec. Increased freezing time (**I**) in FC, two-sided unpaired Student’s *t* test, *p = 0.0143 vs. Vec. *n* = 9 (Vec) and 8 (P1) mice. **J**, **K** P1 administration significantly decreased hippocampal Aβ plaque content measured by Th-S staining. *n* = 5 mice per group, two-sided unpaired Student’s *t* test, **p = 0.0054 vs. Vec. Bar = 50 μm. **L**, **M** Increased MME expression and STAT3 phosphorylation (Y705) in hippocampal lysates following P1 treatment, measured by Western blotting. *n* = 3 mice per group, two-sided unpaired Student’s *t* test, *p = 0.0234 (MME), **p = 0.0061 (pY-STAT3) vs. Vec. **N**, **O** Co-IP using anti-CD31 from hippocampal lysates showing reduced CD31–STAT3 association after P1 treatment, while CD31–SHP-2 association remained unchanged. *n* = 3 mice per group, two-sided paired Student’s *t* test, *p = 0.0451 vs. Vec. **P**, **Q** P1 treatment significantly enhanced microglial arborization in hippocampus, measured by immunofluorescence staining and Sholl analysis. *n* = 3 mice per group, two-way RM ANOVA with Šídák post hoc test, *p = 0.0305 (5 μm) vs. Vec. Bar = 20 μm. **R**, **S** P1 treatment significantly promoted microglia migration to the Aβ plaques, evidenced by an increased number of IBA1^+^ microglia (red) within 25 μm of Th-S⁺ Aβ plaques (green). *n* = 3 mice per group, two-sided unpaired Student’s *t* test, *p = 0.0468 vs Vec. Bar = 25 μm. Data are presented as the mean ± SEM. Source data are provided as a Source data file.

To evaluate the therapeutic potential of P1 on cognitive function and amyloid pathology, 6-month-old 5×FAD mice were administered P1 (1 mM in 5 µL) or vehicle (PBS) via intracerebroventricular injection through a guiding cannula once every two days for one month (Fig. 7A). Throughout the 4-week treatment period, body weight was monitored and showed no significant differences between groups (Supplementary Fig. 9A), indicating that repeated intracerebroventricular administration of P1 did not affect general health status. Behavioral assessments and histological analyses were performed. In the OF test, P1 administration did not significantly alter total distance traveled or the time spent in the center area (Fig. 7B, C), indicating no effect on general locomotor activity or anxiety-like behavior. In contrast, the NOR test showed that P1-treated 5×FAD mice exhibited a significantly higher discrimination index than the vehicle-treated 5×FAD mice, suggesting improved recognition memory (Fig. 7D). The MWM test was used to assess spatial learning and memory. During the training phase, P1-treated mice demonstrated significantly reduced latency to locate the hidden platform compared with the vehicle-treated 5×FAD mice (Fig. 7E), indicating enhanced learning ability. In the probe trial, P1 treatment significantly increased the number of platform crossings and the time spent in the target quadrant, with no significant change in swimming speed (Fig. 7F–H), suggesting improved spatial memory. In the FC test, the percentage of freezing time was significantly increased in P1-treated 5×FAD mice relative to the controls (Fig. 7I), further supporting the enhanced hippocampus-dependent memory performance. After behavioral testing, major organs were collected for histological and weight analyses. Organ weights (heart, liver, spleen, lung, and kidney) were comparable between groups (Supplementary Fig. 9B), and hematoxylin and eosin staining revealed no detectable histopathological abnormalities (Supplementary Fig. 9C), indicating that P1 administration did not induce systemic toxicity. In addition, FITC perfusion analyses showed no significant differences in cerebral vascular density or microvascular leakage between P1- and vehicle-treated mice (Supplementary Fig. 9D–F), indicating that P1 does not measurably disrupt endothelial CD31-mediated vascular integrity under our experimental conditions.

To assess amyloid pathology, hippocampal sections were subjected to Thioflavin-S staining. Quantitative analysis revealed that P1 administration significantly reduced the area of thioflavin-S positive Aβ plaques (Fig. 7J, K), indicating attenuation of fibrillar Aβ deposition following treatment. To further investigate the molecular mechanisms underlying the cognitive and pathological improvements induced by P1, hippocampal tissues were subjected to biochemical and interaction analyses. Western blot analysis demonstrated that P1 treatment increased MME expression and STAT3 phosphorylation (Fig. 7L, M). In contrast, P1 treatment did not significantly alter APP, PS1, or BACE1 expression (Supplementary Fig. 10), excluding reduced Aβ production as the primary mechanism. Co-immunoprecipitation assays further revealed that P1 reduced the interaction between CD31 and STAT3 in vivo, while CD31–SHP-2 association remained unchanged (Fig. 7N, O), supporting the notion that P1 disrupts CD31–STAT3 binding and thereby enhances STAT3 activation. To assess the effects of P1 on microglia, immunofluorescence staining showed that P1 treatment enhanced microglial arborization in the hippocampus (Fig. 7P, Q) and promoted microglial migration toward Aβ plaques (Fig. 7R, S), indicating enhanced microglial engagement with plaques and facilitated Aβ clearance in vivo.

Collectively, these data demonstrate that blocking CD31-STAT3 interaction in vivo by intracerebroventricular administration of P1 can effectively improve multiple domains of AD-like cognitive dysfunctions with a significant reduction of hippocampal Aβ plaque burden. Therefore, P1 treatment can ameliorate AD in both pathology and behavioral levels.

## Discussion

Microglia, the primary immune cells in the central nervous system, constantly monitor and respond to pathological changes within the brain^29^. Studies show that microglia play multiple crucial roles in AD pathogenesis, especially in Aβ clearance^30,31^, Tau protein propagation^12,13,32^, inflammation^33,34^, and synaptic damages^35,36^. Therefore, discovering factors that affect microglial function is essential for effective AD intervention strategies. Single-cell transcriptomics analyses revealed that CD31, a well-known endothelial cell-associated protein, is also highly expressed in microglia^20,25^. However, there has been no prior experimental evidence showing the CD31 expression pattern in CNS neural cells, nor the impact of CD31 on microglial functionality and AD pathology. In the present study, we demonstrated experimentally that CD31 was predominantly expressed in microglia but not in neurons and astrocytes. We also found that CD31 expression was significantly increased in the microglia of AD patients and 5×FAD mice. Microglia-specific knockdown of CD31 mitigated microglia activation with reduced DAM number and markedly restored expression of multiple microglia subtype-specific functional genes, which led to microglial transcriptomes reprogrammed toward a homeostatic state. These transcriptional improvements contributed to the reduced Aβ plaque burden, dampened inflammatory responses, and ultimately improved cognitive performance in AD mice. The underlying mechanisms involve the simultaneous interaction of CD31 with STAT3 and SHP2, which facilitated the dephosphorylation and subsequent inhibition of STAT3 transcriptional activity. Knocking down CD31 restored and enhanced STAT3 phosphorylation, activated transcription of the downstream enzyme MME, and boosted Aβ degradation in microglia. This led to a reduction in Aβ deposition and an improvement in cognitive outcomes (Supplementary Fig. 11). The findings reveal the significant role of the CD31/STAT3/MME pathway in modulating microglial-mediated AD pathology.

In the present study, we validated microglial expression of CD31 through multiple experimental approaches. We verified dominant CD31 expression in primary microglia but not in astrocytes or neurons by Western blotting. By immunofluorescent staining, we showed colocalization CD31 with IBA1, confirming microglia-specific expression in the brain. It should be noted that CD31 is a canonical endothelial junctional protein, and endothelial cells therefore account for the majority of total CD31 protein in the brain. Consistent with this, microglia-specific deletion of CD31 resulted in only a partial (~20–25%) reduction in bulk hippocampal CD31 levels (Supplementary Fig. 2), indicating that microglia are not the dominant quantitative source of CD31. In this study, we employed a microglia-specific heterozygous CD31 deletion model. Although a homozygous knockout was not included, our data demonstrate that partial reduction of CD31 is sufficient to produce robust functional improvements. Future studies will further assess whether more complete CD31 ablation confers additional effects. This observation reflects the relative contribution of microglia to overall CD31 abundance rather than contradicting the cell-type specificity of CD31 expression revealed by our cellular and transcriptomic analyses. We also observed that Aβ enhanced CD31 expression in BV-2 cells and primary microglia. Previous transcriptomic data showed that treatment of human iPSC-derived microglia with oligomeric Aβ resulted in a significant increase of CD31 transcripts^24^. Our findings, align with these observations, suggest a direct link between Aβ pathology and CD31 expression. Notably, increased CD31 expression was also detected in microglia within the brains of 5×FAD mice. These in vitro and in vivo experiments indicate that microglia richly express CD31, and high level of microglial CD31 expression is involved in the progression of AD. Notably, bulk abundance does not necessarily predict disease relevance: among non-endothelial neural cells, CD31 is selectively enriched in microglia and is markedly upregulated in AD, placing microglial CD31 within a dynamic, pathology-responsive pool. This framework aligns with recent human microglia profiling studies that highlight disease-associated microglial state remodeling in AD and support the concept that changes in a specific microglial gene module can exert disproportionate functional effects on microglial activation programs, Aβ clearance, and behavioral outcomes^24,37,38^.

Microglia undergo profound and dynamic state transitions during AD, giving rise to transcriptionally and functionally distinct subpopulations that exert context-dependent effects on amyloid pathology, neuroinflammation, and synaptic integrity. Early single-cell studies identified disease-associated microglia (DAM) as a core plaque-responsive state enriched for genes involved in lipid metabolism and phagocytosis^16^, while subsequent work has expanded this framework by demonstrating that microglial activation in AD is neither linear nor uniform but instead composed of multiple, partially overlapping programs that vary across disease stage, anatomical location, and pathological context^39,40^. More recent studies have further highlighted interferon-responsive microglia (IRM) as a distinct transcriptional state induced by amyloid and tau pathology, with direct implications for synaptic dysfunction and cognitive decline^14,41,42^. Although key upstream drivers of microglial state transitions—most notably the TREM2–APOE axis—have been shown to promote the shift from homeostatic to neurodegeneration-associated programs^43,44^, mechanistically, TREM2 signaling is initiated through its association with the adaptor protein TYROBP (DAP12), leading to ITAM phosphorylation and recruitment of Syk kinase, which transduces intracellular activation signals^3,17^. However, substantially less is understood about how these disease-linked microglial states are mechanistically coupled to the transcriptional regulation of proteolytic Aβ clearance, as opposed to phagocytosis alone. In this context, our findings identify CD31 as a microglia-enriched, pathology-responsive molecule that links disease-associated signaling to STAT3-dependent transcriptional control of the Aβ-degrading enzyme MME. These results place CD31 within the contemporary framework of microglial state remodeling and provide a mechanistic connection between microglial transcriptional programs and enzymatic Aβ clearance in AD.

In light of this gap, our single-nucleus transcriptomic analyses provide a state-level framework for understanding how modulation of a disease-responsive microglial molecule reshapes microglial programs in the 5×FAD mouse brain, thereby setting the stage for subsequent mechanistic analyses. Microglial activation during AD is increasingly recognized as a dynamic and heterogeneous process, giving rise to multiple transcriptionally and functionally distinct states rather than a single linear trajectory^38,40,43^. Within this established framework, our data indicate that microglia-specific CD31 knockdown induces a coordinated, program-level remodeling of microglial states rather than selectively targeting a single disease-associated cluster. This remodeling is supported by an increased representation of microglia expressing canonical homeostatic markers, together with a concomitant reduction in both disease-associated microglia (DAM) and interferon-responsive transcriptional programs in the AD brain. Such global redistribution of transcriptional programs is consistent with the emerging view that neurodegenerative disease reshapes the balance of microglial states in a context-dependent manner, as supported by large-scale human single-cell/nucleus datasets^24,45^. In addition to changes in microglial state composition, CD31 knockdown was associated with marked transcriptional remodeling within the DAM program itself. In parallel, CD31 knockdown downregulated actin filament-based processes, cell–matrix adhesion, and GTPase activation pathways, indicative of reduced cytoskeletal remodeling and microglial activation. Moreover, expression of key DAM-associated genes involved in proteolysis and repair, such as *Cst7*, *Cd9*, and *Ctsd*, was significantly increased, further supporting the shift toward a clearance-competent, tissue-supportive phenotype. Together, these molecular changes imply that CD31 knockdown alleviates pathological features of DAM by promoting degradative and reparative programs while dampening pro-inflammatory activation. Importantly, restoration of homeostatic microglial programs has been functionally linked to improved disease outcomes. TMEM119 has been widely used as a homeostatic microglial marker in mouse and human studies^46,47^. Notably, recent work further suggests a functional role of TMEM119 in amyloid-related phenotypes: TMEM119 deficiency aggravated disease progression, whereas microglial TMEM119 overexpression enhanced Aβ handling and alleviated cognitive deficits in 5×FAD mice^48^. In line with these reports, we observed that CD31 knockdown significantly upregulated *Tmem119*, suggesting an increased abundance of homeostatic microglial phenotypes that may contribute to attenuation of AD-related pathology. Notably, the reduction of interferon-responsive microglial programs following CD31 modulation is highly consistent with prior studies demonstrating that interferon-driven microglial activation exacerbates synaptic dysfunction and amyloid-associated pathology in both mouse models and human AD^14,41,42^. Collectively, these findings position CD31 as a regulator of microglial state balance, integrating disease-associated signaling with transcriptional programs that shape microglial responses during AD. In addition to transcriptional state remodeling, we also observed a reduction in overall microglial density in *Cx3cr1*^Cre^CD31^fl/+^5×FAD mice. Given that Aβ pathology is known to drive microglial proliferative expansion in 5×FAD models, the reduced microglial abundance likely reflects attenuated proliferation secondary to decreased amyloid burden rather than increased microglial loss. Although CD31 knockdown is genetically restricted to microglia, the observed restoration of synaptic protein expression and dendritic spine density suggests that microglial reprogramming exerts beneficial non–cell-autonomous effects on neuronal integrity. Given the well-established role of microglia in regulating synaptic remodeling and circuit stability, these findings support a model in which microglial CD31 deficiency improves the neuronal microenvironment, thereby contributing to enhanced synaptic plasticity and cognitive performance in 5×FAD mice. Further studies will be required to dissect the precise intercellular signaling mechanisms underlying these broader network-level effects.

While these transcriptomic analyses establish that CD31 modulates microglial state programs and downstream neuronal integrity at a global level, they do not by themselves explain how such state remodeling translates into altered amyloid handling. Given that microglial states differ not only in inflammatory tone but also in their capacity for Aβ clearance, we next sought to determine whether CD31 knockdown engages specific molecular pathways that directly regulate proteolytic Aβ degradation. Consistent with the observed state-level remodeling, targeted CD31 knockdown was accompanied by a marked attenuation of Aβ pathology in 5×FAD mice. Microglia can clear Aβ through various Aβ-degrading enzymes, such as IDE, MME, and MMP^49–51^. Among these, MME was identified as the primary downstream effector of CD31 knockdown. Mechanistically, CD31 reduction increased STAT3 phosphorylation at Tyr705, enhanced its binding to the MME promoter, and promoted MME transcription. Pharmacological inhibition of STAT3 abolished both MME upregulation and the enhanced Aβ degradation, supporting a STAT3-dependent mechanism.

STAT3 phosphorylation is regulated by kinases and phosphatases, primarily JAK and SHP2. As CD31 knockdown did not affect JAK signaling, our data support a model in which CD31 facilitates SHP2-mediated dephosphorylation of STAT3 through direct interaction. Disruption of CD31 reduces this interaction, thereby enhancing STAT3 phosphorylation. TREM2/TYROBP/Syk signaling activates downstream intracellular pathways associated with microglial activation and survival, and can interface with STAT3-associated transcriptional programs^52^. In line with this, our findings provide a potential mechanistic explanation linking CD31 to TREM2-driven signaling. In our study, we demonstrate that microglial CD31 directly interacts with STAT3 and SHP2, facilitating SHP2-mediated dephosphorylation of STAT3 and consequently suppressing STAT3-dependent transcriptional activity. We therefore propose that, during AD progression, pathological upregulation of CD31 in microglia may impose an inhibitory constraint on STAT3 signaling. Such CD31-mediated dephosphorylation of STAT3 could partially counterbalance STAT3 activation downstream of the TREM2/TYROBP/Syk pathway, thereby attenuating the transcriptional output of TREM2 signaling despite its activated state. In this model, CD31 and TREM2 converge on STAT3 as a shared downstream node but exert opposing regulatory effects—TREM2 promotes STAT3 activation, whereas CD31 restrains STAT3 activity through SHP2-dependent dephosphorylation. This antagonistic regulation offers a coherent framework to reconcile the coexistence of TREM2 pathway activation and impaired microglial protective capacity in AD, positioning CD31 as a negative checkpoint within disease-associated microglial signaling networks.

More interestingly, using a peptide (P1) based on the CD31 (722–738) sequence to disrupt the CD31–STAT3 interaction in BV-2 cells significantly enhanced the degradation of Aβ by these cells. Consistently, P1 treatment in 5×FAD mice improved cognitive performance across multiple behavioral tests and reduced hippocampal Aβ plaque burden, supporting the in vivo relevance of the CD31-STAT3 axis in modulating AD pathology. Targeting the CD31–STAT3 interaction, therefore, represents a promising therapeutic strategy for mitigating neuroinflammation and Aβ pathology in AD.

Although the present study delineates downstream mechanisms by which CD31 regulates microglial state programs and amyloid clearance, the upstream signals driving CD31 upregulation during AD progression remain to be fully defined. Our data, together with prior transcriptomic studies, indicate that Aβ itself is sufficient to induce CD31 expression in microglia. Such regulation may arise from Aβ activation of innate immune and scavenger receptor pathways in microglia^34^, thereby engaging downstream inflammatory signaling cascades and activating a set of transcription factors that may potentially regulate CD31 expression. Aβ exposure also elicits oxidative stress and reactive oxygen species in microglia^53,54^, further amplifying these transcriptional responses. In addition, amyloid-associated interferon signaling^41,42^, metabolic reprogramming toward glycolysis^55^, and microenvironmental changes linked to neuronal injury and neurovascular dysfunction^56,57^ may collectively contribute to sustained CD31 induction during disease progression.

From a translational perspective, although intracerebroventricular administration was employed here to establish proof of concept, ongoing advances in peptide and protein delivery to the central nervous system offer feasible paths toward clinical translation. Chemical modification strategies and nanocarrier-based delivery systems have shown promise in improving blood–brain barrier penetration and brain bioavailability of peptide-based therapeutics^58,59^. Future optimization of the P1 peptide using such approaches may enable peripheral administration while preserving effective brain targeting, thereby supporting further preclinical development without altering the mechanistic framework defined in this study.

In summary, this study identifies CD31 as a microglia-enriched regulator that links disease-associated microglial state remodeling to impaired amyloid clearance in AD. By coupling microglial activation programs to STAT3-dependent transcriptional control of proteolytic pathways, CD31 provides a molecular bridge between microglial state dynamics and Aβ homeostasis. These findings expand current understanding of how microglial signaling networks shape AD progression and highlight microglial CD31 as a potential therapeutic target for restoring homeostatic microglial functions in neurodegenerative disease.

## Methods

### Ethics

All animal experiments were approved by the Animal Care and Use Committee of Huazhong University of Science and Technology and were conducted in accordance with relevant ethical regulations ([2022] IACUC 2751).

### Materials and reagents

Sacubitrilat (HY-17620) and Stattic (HY-13818) were purchased from MedChemExpress (Shanghai, China). SHP099 (T3564) was purchased from TargetMol (Shanghai, China). Fluorescein5(6)-isothiocyanate (catalog no. 13081045311) was purchased from Klamar-Reagent (Shanghai, China). Plasmids pEGFP-C1-CD31(621-738), pEGFP-C1-CD31(621–681), pEGFP-C1-CD31(682–721), pEGFP-C1-CD31(722–738), p3×Flag-STAT3 were generated by Tsingke (Wuhan, China). FITC-labeled human Aβ_1–42_ and peptide 1 were generated by QyaoBIO (Shanghai, China). Newly generated plasmids and peptides are available from the corresponding author upon request. The antibodies used in this study are listed in Supplementary Data 1.

### Cell culture and treatments

HEK293 and BV-2 cells were maintained as continuous lines in our laboratory and cultured in DMEM supplemented with 10% fetal bovine serum (FBS) in a humidified atmosphere with 5% CO_2_ at 37 °C, and passaged regularly at approximately 90% confluency. HEK293 cells were authenticated by STR profiling (20 STR loci plus Amelogenin), showing a 98.31% match to the HEK293 reference profile in the Cellosaurus database; BV-2 cells were not authenticated. All cell lines were routinely tested for mycoplasma contamination and confirmed to be negative. HEK293 is listed on the ICLAC Register of Misidentified Cell Lines and was used solely as a heterologous expression system for transient transfection-based assays, where conclusions do not depend on its tissue origin.

Primary astrocytes and microglia were isolated from neonatal mouse pups using a mixed glial culture protocol followed by mild trypsinization-based separation. Briefly, pups were anesthetized by hypothermia, and brain tissues were dissected in ice-cold DMEM/F-12 (1:1) medium. After careful removal of the meninges, the cortical tissues were cut into small pieces (approximately 1 mm³) and digested in 0.125% Trypsin-EDTA at 37 °C for 20 min. Digestion was terminated by adding complete culture medium (DMEM/F-12 supplemented with 10% FBS, 1% sodium pyruvate, and 1% penicillin-streptomycin). The tissue was triturated into a single-cell suspension using a P1000 pipette tip and filtered through a 45 µm cell strainer. Cells were plated onto culture dishes pre-coated with 5 µg/mL poly-D-lysine and cultured at 37 °C with 5% CO_2_. The medium was refreshed every 3 days until the mixed glial cultures reached 100% confluency (approximately 10–12 days in vitro).

To separate the two cell populations, the confluent mixed glial cultures were subjected to mild trypsinization using a concentration of 0.0625% Trypsin-EDTA at 37 °C. This process was monitored until the complete detachment of the upper intact cell layer (astrocytes) occurred, while the bottom layer (microglia) remained attached. For microglia, the cells remaining adherent to the culture plate were identified as microglia. The trypsin solution was removed, and fresh complete medium was added for maintenance or downstream experiments. For astrocytes, the detached upper layer containing the astrocytes was collected from the supernatant. The suspension was centrifuged, and the cell pellet was resuspended in complete medium and replated into new culture flasks for further cultivation.

Primary neurons were isolated from E17 mouse embryos following the dissociation protocol described above, but maintained in serum-free Neurobasal medium supplemented with 2% B27 and 1% GlutaMAX™. Cells were seeded onto poly-D-lysine-coated plates, with half-medium changes performed every 3 days.

To induce Aβ-related cellular responses, primary microglia and BV-2 cells were incubated with pre-formed oligomeric Aβ_1__–__42_ at a final concentration of 5 μM for 24 h at 37 °C. To inhibit MME activity, cells were treated with the MME inhibitor sacubitrilat (5 nM) for 24 h at 37 °C. To inhibit STAT3 activity, cells were treated with the STAT3 inhibitor Stattic (20 μM) for 24 h at 37 °C. For P1 treatment, BV-2 cells were exposed to P1 at a dose gradient of 0, 6.25, 12.5, 25, 50, and 100 μM for 24 h at 37 °C. Primary microglia were treated with P1 at a concentration of 100 μM for 24 h at 37 °C.

### Animals and drug administration

Wild-type C57BL/6J mice (RRID: IMSR_JAX:000664) were purchased from Beijing Vital River Laboratory Animal Technology Co., Ltd. 5×FAD transgenic mice [B6.Cg-Tg(APPSwFlLon,PSEN1*M146L*L286V)6799Vas/Mmjax; RRID: MMRRC_034848-JAX] were obtained from Shulaibao (Wuhan) Biotechnology Co., Ltd. and maintained on a C57BL/6J background. Cx3cr1-Cre mice [B6J.B6N(Cg)-Cx3cr1tm1.1(cre)Jung/J; RRID: IMSR_JAX:025524], a constitutive Cre line, were obtained from Cyagen Biosciences (project ID C001032). Pecam1 (CD31) conditional knockout mice [C57BL/6N-Pecam1em1Cyagen; serial number CKOCMP-18613-Pecam1; contract number CKOAI191104XW1-B] were custom-generated by Cyagen Biosciences on a C57BL/6N background using CRISPR/Cas9-mediated loxP insertion flanking exons 8 to 11 of the Pecam1 gene. To generate AD model mice with microglia-specific CD31 knockdown, Cx3cr1-Cre;CD31^fl/fl^ mice were crossed with 5×FAD mice to obtain Cx3cr1-Cre;CD31^fl/+^;5×FAD heterozygous offspring, which were used for all subsequent experiments. Adult male mice (6 months old at the time of experiments unless otherwise noted) were used in this study; both male and female mice (2 males and 1 female per group) were used for snRNA-seq. Mice were housed under specific pathogen-free conditions with a 12 h light/dark cycle, ambient temperature of 24 ± 2 °C, relative humidity of 40–70%, and ad libitum access to food and water. Because the floxed Pecam1 allele was generated on a C57BL/6N background while the other strains were maintained on a C57BL/6J background, experimental cohorts were on a mixed C57BL/6J × C57BL/6N background; to control for residual background variation and cage effects, all comparisons were performed using co-housed, age- and sex-matched littermate controls obtained from the same crosses. At the experimental endpoint, mice were deeply anesthetized with 1% pentobarbital sodium and euthanized by transcardial perfusion for histological analyses or by decapitation for biochemical and transcriptomic analyses.

For intracerebroventricular peptide delivery, guiding cannulas (RWD, Shenzhen, China) were stereotaxically implanted into the lateral ventricle of 6-month-old 5×FAD male mice under isoflurane anesthesia. Cannula placement was performed using the following coordinates relative to bregma: posterior 0.22 mm, lateral 1.0 mm, and ventral −2.5 mm from the skull surface. Mice were randomly assigned to two groups: a control group receiving saline (*n* = 9) and a P1-treated group (*n* = 8). P1 was dissolved in saline and administered at a concentration of 1 mM with an injection volume of 5 μL using an automated microinjection system (RWD, Shenzhen, China), once every two days. During peptide administration, mice were gently restrained in a custom-designed holder and remained awake.

### Open field test

The open field test was performed in a white square arena divided into 16 equal regions, with the central area defined as four squares. Mice were habituated to the testing room one day in advance. Each mouse was placed in the center of the arena and allowed to explore freely for 5 min. Locomotor activity was recorded using a video tracking system (Chengdu Taimeng Software Co., Ltd, China).

### Novel object recognition test

Mice were allowed 5 min for free exploration of objects A and B in the test chamber in the first day. In the second day, the mice were allowed 5 min for free exploration of object A and C (replacement of object B). The exploring time of object A and C in the second day was recorded as *TA* and *TC,* respectively. Recognition index (RI) was calculated by equation RI = *TC*/(*TA* + *TC*). Discrimination index (DI) was calculated by equation DI = (*TC *− *TA*)/(*TA* + *TC*).

### Morris water maze test

Mice were allowed to search for the hidden platform in the water maze at the training session. The training session contains 6 days, and each mouse was trained for 3 times every day. After a day of rest, the platform was removed, and the mice were allowed 60 s for free exploration. Swimming path, speed, latency, and time of crossing the targeted platform or the previous platform-located quadrant was recorded by the tracking system (TaiMeng, Chengdu).

### Fear conditioning test

For the fear conditioning test, the mice were placed in the test chamber for 3 min, during which 3 times of 0.9 mA electric shock lasting 3 s was given in the first day. In the second day, the mice were allowed 3 min for free exploration in the test chamber without electric shock and freezing time was recorded.

### Western blotting

Brain tissues or cell cultures were homogenized with RIPA Lysis Buffer (Beyotime, P0013B) and placed on ice for 30 min. After centrifugation at 13,400 × *g*, 4 °C for 30 min, the supernatant was collected and supplemented with SDS-PAGE loading buffer for denature in boiling water for 10 min. The samples were separated by SDS-PAGE gel and then transferred onto nitrocellulose membranes. The membranes were blocked in 5% non-fat milk at room temperature for 1 h and incubated with primary antibodies at 4 °C overnight. After washes with TBST, the membranes were incubated with secondary antibodies at room temperature for 1 h and washed again with TBST, and chemiluminescent signals were captured on an ECL detection system (ClinX ChemiScope 6000).

### Co-immunoprecipitation

Cultured cells were homogenized with cell lysis buffer for Western and IP (Beyotime, P0013) and centrifuged at 4 °C, 12,000 × *g* for 30 min. The supernatant was collected and incubated with primary antibodies at 4 °C overnight, and then incubated with protein A + G magnetic beads (Beyotime, P2108) at 4 °C for 4 h. After washing with PBS, the beads were boiled with 2×SDS-PAGE loading buffer for 10 min and the supernatant was collected for further analysis.

### Immunostaining

Mice were deeply anesthetized with 1% pentobarbital sodium and perfused transcardially with saline, followed by 4% paraformaldehyde. Brains were collected, post-fixed in 4% paraformaldehyde at 4 °C for 24 h, and cryoprotected in 20% and 30% sucrose solutions for 2 days. Coronal sections (40 μm) were prepared using a cryostat microtome (Leica CMG1900). Cultured cells were fixed in 4% paraformaldehyde for 15 min at room temperature and stored in PBS at 4 °C. For immunofluorescence, brain sections or cell slides were blocked and permeabilized in PBS containing 5% BSA and 0.5% Triton X-100 for 1 h at room temperature, then incubated with primary antibodies overnight at 4 °C. After PBS washes, Alexa Fluor-conjugated secondary antibodies were applied at 37 °C for 1 h, followed by DAPI counterstaining for 10 min at room temperature. Finally, sections were mounted with PBS containing 50% glycerol, and images were captured using a two-photon confocal microscope (LSM800, Zeiss) operated with ZEN software (2012, black edition 8.0.0.273). Apoptotic cells were detected using a TUNEL assay kit according to the manufacturer’s instructions (C1086, Beyotime Biotechnology), and TUNEL-positive signals were visualized using fluorescence microscopy.

### Proximity ligation assay

Proximity ligation assay (PLA) was performed using the Duolink® In Situ PLA kit (Sigma-Aldrich, DUO92002, DUO92004) according to the manufacturer’s instructions. Briefly, after primary antibody incubation, PLA probes were applied, followed by ligation and amplification steps. Signals were visualized as distinct fluorescent dots under a confocal microscope.

### Golgi-Cox staining

Brain tissue was immersed in Golgi-Cox staining solution (2.5 g K_2_Cr_2_O_7_, 2.5 g HgCl_2_ and 2 g K_2_CrO_4_ dissolved in 240 mL ddH2O) at room temperature in the dark for 3 weeks, changed into protective solution (150 g sucrose, 5 g PVP-40000 and 150 mL ethanol dissolved in 200 mL PB) at room temperature in the dark for 2 days, and sliced into 100 μm sections. After washed in ddH_2_O, the section was sequentially incubated in 50% ethanol for 5 min, 15% ammonia solution for 8 min, 5% Na_2_S_2_O_3_ solution for 10 min, and transferred onto 2% gelatin coated slides. After dehydrated through graded ethanol series, cleared in xylene, and mounted with neutral balsam, images were taken by Pannoramic SCANⅡ (3DHISTECH).

### Thioflavin S staining

0.1% thioflavin S was dissolved in 50% ethanol and filtered through 0.22 μm strainer to acquire the thioflavin S staining solution. Free-floating brain sections or fixed cell cultures were permeabilized in PBS containing 0.5% Triton X-100 at room temperature for 30 min and incubated in Thioflavin S staining solution in the dark at room temperature for 5 min. After washes with 50% ethanol and PBS, the samples were co-stained with DAPI and mounted with PBS containing 50% glycerol.

For the quantification of microglia surrounding amyloid plaques, imaging parameters were optimized to clearly visualize microglial cell bodies along with their processes. Microglia were co-stained with DAPI, and only IBA1-positive cells with an associated DAPI-positive nucleus were counted as individual microglia. This approach allowed reliable identification and counting of microglial cell bodies in plaque-adjacent regions despite the dense microglial network.

### FITC perfusion for assessment of cerebrovascular leakage

Mice were anesthetized and subjected to transcardial perfusion. Blood was first cleared using phosphate-buffered saline supplemented with heparin (5 U/ml) and glucose (10 mM), followed by perfusion with fluorescein isothiocyanate (FITC; 0.1 mg/ml) dissolved in glucose-containing PBS to label the cerebral vasculature. Animals were subsequently perfused with paraformaldehyde in phosphate buffer for fixation. Brains were collected, post-fixed, cryoprotected, and sectioned. FITC fluorescence was visualized using fluorescence or confocal microscopy. Vascular structures were identified by intravascular fluorescence, and leakage was assessed based on the presence of FITC signal in extravascular regions.

### Quantitative real-time PCR

Total RNA was isolated by using the Trizol kit (15596026, Invitrogen), and HiScript III RT SuperMix kit (R323-01, Vazyme) was used to obtain cDNA. RT-qPCR was performed with ChamQ Universal SYBR qPCR Master Mix (Q711-02, Vazyme) using a StepOnePlus Real-Time PCR Detection System (272001262, AB Applied Biosystems) with StepOne Software (v2.3). The primer's information is as follows:

MME-F: GACTCTGGCTTCGTCCCC

MME-R: CCCATCACCTAAAATCAGTGGGA

TNF-α-F: GTGACAAGCCTGTAGCCCAC

TNF-α-R: GCAGCCTTGTCCCTTGAAGA

IL-6-F: ACCGCTATGAAGTTCCTCTC

IL-6-R CTCTGTGAAGTCTCCTCTCC

IL-1β-F: TGCCACCTTTTGACAGTGATG

IL-1β-R: AAGGTCCACGGGAAAGACAC

IL-10-F: CCAAGGTGTCTACAAGGCCA

IL-10-R: GCTCTGTCTAGGTCCTGGAGT

β-actin-F: ATGCCCTGAGGCTCTTTTCC

β-actin-R: CAGCTCAGTAACAGTCCGCC

### Chromatin immunoprecipitation assay

Chromatin immunoprecipitation assay was conducted with a commercially available kit, and all the procedures followed the instruction of EZ-ChIP™ (17-371, Merck). Anti-STAT3 (ab68153, Abcam) was used for protein concentration. The primers for RT-qPCR detection were as follows:

MME (motif-1)-F: ACATTGAAATCCTGGGGCAG

MME (motif-1)-R: CCCCAGTCTATGTGTGTCTCT

MME (motif-2)-F: GGAAAACGGAGAGAGCAAGAG

MME (motif-2)-R: GCTTCGCCAGACATTCTTGG

### Dual luciferase reporter gene assay

Luciferase reporter plasmid pGL-3 expressing MME motif 1 or 2 and pGMLR-TK as constructed by Tsingke (Wuhan). Coordinate luciferase reporter lentivirus was packaged by Obiosh (Shanghai). All the procedures followed the instruction of the Dual Luciferase Reporter Gene Assay Kit (11402ES60, Yeasen).

### Detection of Aβ

Hippocampus or cortex tissue and cultured cells were homogenized in PBS supplemented with protease inhibitors cocktail and centrifuged at 4 °C, 2200 × *g* for 30 min. The supernatant was collected for detection. Aβ_1–40_ and Aβ_1–42_ were measured by a commercial ELISA kit (Elabscience, E-EL-H0542, E-EL-H0543); all procedures followed the manufacturer’s instructions, and absorbance was read on a BioTek Synergy H1 microplate reader using Gen5 software (v2.09).

### Preparation of oligomeric Aβ and the Aβ phagocytosis assay

Synthetic Aβ_1–42_ peptide was dissolved in 50% DMSO to generate a 2.5 mM stock solution, which was aliquoted and stored at −80 °C. To prepare oligomeric Aβ, the stock solution was diluted in high-glucose DMEM to a final concentration of 50 μM and incubated at 4 °C for 24 h. Primary microglia isolated from wild-type and microglia-specific CD31 knockdown mice were cultured as described above and treated with fluorophore-labeled oligomeric Aβ_1__–__42_ at a final concentration of 5 μM. After incubation for 2 h at 37 °C to allow Aβ uptake, extracellular Aβ was completely removed by extensive washing with PBS. Fluorescence intensity measured immediately after washing (defined as time 0 h) was used to indicate the amount of internalized Aβ. Intracellular degradation of ingested Aβ was subsequently assessed by monitoring fluorescence intensity over a 9 h period.

### Bulk transcriptomics

Total RNA was extracted from primary microglial cells treated with Aβ oligomers for 24 h, including both control and CD31 knockdown groups. mRNA enrichment was carried out using magnetic beads with Oligo(dT) to selectively isolate polyA-tailed mRNA. The enriched mRNA was then fragmented using a fragmentation buffer, followed by reverse transcription with random N6 primers. The second cDNA strand was synthesized, resulting in double-stranded DNA. The double-stranded DNA was end-repaired, 5′ phosphorylated, and an “A” was added to the 3′ end. The DNA was then ligated to a bubble-like adapter with a protruding “T” at the 3′ end. PCR amplification was performed with specific primers, and the PCR products were denatured into single strands. These strands were circularized using a bridging primer to form a single-stranded circular DNA library. The quality of the constructed library was checked, and sequencing was performed after passing quality control. For bioinformatic analysis, raw reads were quality-trimmed with fastp (v0.20.0), aligned to the mm10-2020-A mouse reference genome, and gene-level read counts were quantified using an annotation-based counting approach. Differential expression analysis between CD31 knockdown and WT microglia (Aβ-treated) was performed with DESeq2 (v1.44.0) using a negative binomial model; genes with a Benjamini–Hochberg-adjusted *P* value (FDR) < 0.05 were considered differentially expressed. Gene Ontology biological-process and KEGG pathway enrichment analyses were performed with clusterProfiler (v4.12.6), using Benjamini–Hochberg correction for multiple testing, on upregulated and downregulated gene sets to identify biological processes and signaling pathways associated with CD31 knockdown. Heatmaps and enrichment plots were generated based on normalized expression values and/or enrichment statistics.

### Nuclei isolation

Littermate wild-type mice were used as controls. Nuclei were isolated from whole-brain tissue, excluding the cerebellum. Each genotype group included three mice, consisting of two males and one female. Single-nucleus isolation was performed from frozen brain tissue. Tissue was homogenized on ice using a glass Dounce homogenizer in freshly prepared Lysis Buffer composed of 10 mM Tris-HCl (pH 7.4), 10 mM NaCl, 3 mM MgCl₂, 0.1% pre-diluted Lysis Reagent (IGEPAL CO-630 or NP-40 substitute), and nuclease-free water to the final volume. The homogenate was incubated on ice for 10 min with gentle swirling and intermittently supplemented with Hibernate E/B27/GlutaMAX medium to maintain nuclear integrity. Tissue was triturated 5–7 times using a fire-polished Pasteur pipette to release intact nuclei. Nuclei were pelleted by centrifugation at 500 × *g* for 10 minutes at 4 °C, and the supernatant was removed carefully without disturbing the pellet. Pellets were resuspended in Nuclei Wash and Resuspension Buffer (1% BSA, 0.2 U/µL RNase inhibitor in 1× PBS), and pipetted gently 10 times to achieve a homogeneous suspension. Large clumps or debris were removed using a 40 µm Flowmi Tip Strainer, followed by a second centrifugation under the same conditions. After washing, nuclei were resuspended to a target concentration of 700–1200 nuclei/µL and quantified using a Countess II FL Automated Cell Counter or hemocytometer. For embryonic brain tissue, myelin debris was removed using Myelin Removal Beads II and LS Columns, following the manufacturer’s instructions. Wide-bore tips were used throughout to minimize mechanical shearing, and all buffers were kept cold. The time between nuclei preparation and loading into the chromium controller was minimized to maintain viability and RNA integrity.

### Library construction and sequencing

Single-nucleus RNA-seq libraries were generated using the Chromium Single Cell 3′ Reagent Kit v3 (10x Genomics, PN-1000075). Each reagent was thawed on ice and mixed gently. Nuclei suspensions were loaded at 2.5–15 µL per channel, corresponding to approximately 700–1200 nuclei/µL, along with the RT Master Mix and barcoded gel beads into Chromium Chip B. GEMs were generated in the Chromium Controller, partitioning individual nuclei with gel beads in nanoliter droplets. Reverse transcription was performed inside the GEMs to attach unique cell barcodes and UMIs to polyadenylated RNA, with incubation at 53–55 °C for 45–60 min, followed by 85 °C for 5 min to inactivate the enzymes.

After GEM generation, the emulsions were broken using the recovery agent supplied by the kit, and the aqueous cDNA was purified using Dynabeads MyOne Silane beads to remove unused primers, enzymes, and oil. The cDNA was amplified by PCR for 12–16 cycles, depending on nuclei input, and purified using double-sided SPRIselect bead cleanup to enrich fragments between ~300–700 bp. Approximately 10 µL of amplified cDNA was then used for library preparation. The cDNA was enzymatically fragmented, end-repaired, and A-tailed, followed by ligation of Illumina-compatible adapters. Indexed PCR was performed to incorporate unique i7 indices for multiplexing, followed by a final SPRIselect cleanup to produce the finished library.

Libraries were quantified using the Qubit dsDNA HS assay and assessed for fragment size distribution on an Agilent Bioanalyzer High Sensitivity chip. Libraries were pooled equimolarly and sequenced on an Illumina NovaSeq 6000 platform, using 28 cycles for Read 1 (cell barcode and UMI), 8 cycles for i7 index, and 90–100 cycles for Read 2 (cDNA insert). Raw sequencing reads were quality trimmed using fastp (v0.20.0) and aligned and quantified with Cell Ranger (v7.1.0) against the mm10-2020-A mouse reference genome to generate gene-barcode count matrices.

### snRNA-seq data analysis

Downstream analyses were performed in R (v4.4.0) using Seurat (v5.3.0). Nuclei with fewer than 10 detected genes or genes expressed in fewer than 500 nuclei were removed, and nuclei with more than 5% mitochondrial transcripts were filtered out. Potential doublets were identified and excluded using DoubletFinder (v2.0.3). Gene expression matrices were normalized with the LogNormalize method (scale factor = 10,000), and 2000 highly variable genes were selected using the vst method. The scaled data were subjected to PCA for dimensionality reduction. Cross-sample integration was initially performed using canonical correlation analysis (CCA) and further corrected for batch effects using Harmony (v1.2.3). Shared nearest-neighbor graphs were built with FindNeighbors using the integrated CCA reduction (integrated.cca), and clusters were identified with FindClusters at a resolution of 0.5. Clustering results were visualized with UMAP.

Cell-type annotation was performed using canonical marker genes and reference-guided label transfer from the Allen Brain Atlas. Transfer anchors were identified using FindTransferAnchors, and predicted labels were assigned using TransferData and incorporated into Seurat metadata. Differentially expressed genes (DEGs) were identified using FindAllMarkers and FindMarkers. For selected comparisons, aggregation-based pseudobulk profiles were generated using AggregateExpression and tested with DESeq2 (v1.44.0). Micro-PVM subclusters (MG0, MG1, and MG5) were analyzed separately, and “reversed genes” were defined as genes showing significant differential expression in opposite directions between conditions. DEGs were defined as *P* < 0.05 and |log2(fold change)| > 0.25, with adjusted *P* < 0.05 for FDR-corrected comparisons.

Cell population proportions for each sample in the microglial Seurat object were calculated as the fraction of total cells assigned to each cluster. Sample-level proportions were arcsine-square-root transformed to stabilize variance for compositional data. Differential abundance between groups (F, F_CD, C) was assessed using an arcsine-square-root transformed limma framework adapted from propeller^60^, in which a design matrix was constructed as ~0 + group and pairwise contrasts were tested using moderated linear models (limma::lmFit) followed by empirical Bayes moderation (eBayes). *P* values were adjusted using the Benjamini–Hochberg FDR, and clusters with FDR < 0.05 were considered significant. Group mean percentages were plotted with standard error of the mean (SEM) and individual sample points overlaid.

Pathway enrichment analysis was performed using clusterProfiler (v4.12.6), with all detected genes as background and DEGs as input for Gene Ontology biological process and KEGG pathway analysis. Benjamini–Hochberg correction was applied, and significantly enriched pathways were defined as those with adjusted *P* < 0.05. Data visualization and figure generation were performed using ggplot2 (v4.0.0), ggnewscale (v0.5.2), RColorBrewer (v1.1.3), scCustomize (v3.0.1), ggrepel (v0.9.6), and scales (v1.4.0).

All analyses were performed in R (v4.4.0) using Seurat v5.3.0, limma v3.60.6, dplyr v1.1.4, tidyr v1.3.1, and DESeq2 v1.44.0.

### Statistical analysis

All data are expressed as mean ± SEM. Analyses and graphical representations were carried out using ImageJ (Fiji v1.52 h) and GraphPad Prism (v10.1.2). Comparisons between two groups utilized the two-tailed Student’s *t* test (paired or unpaired as appropriate). One-way ANOVA, two-way ANOVA or two-way repeated-measures ANOVA followed by appropriate post-hoc tests were used for multiple comparisons. Normality and variance assumptions were evaluated prior to parametric testing. *P* < 0.05 indicates a statistically significant difference. Detailed statistical results are provided in Supplementary Data 2.

### Reporting summary

Further information on research design is available in the Nature Portfolio Reporting Summary linked to this article.

## Supplementary information

Supplementary Information

Description of Additional Supplementary Files

Supplementary Data 1

Supplementary Data 2

Supplementary Data 3

Supplementary Data 4

Reporting Summary

Transparent Peer Review File

## Source data

Source Data

## Data Availability

Raw sequencing data generated in this study have been deposited in the Gene Expression Omnibus (GEO) under accession codes GSE331294 (snRNA-seq; https://www.ncbi.nlm.nih.gov/geo/query/acc.cgi?acc=GSE331294) and GSE331306 (bulk RNA-seq; https://www.ncbi.nlm.nih.gov/geo/query/acc.cgi?acc=GSE331306), and are publicly available. Publicly available datasets reanalyzed in this study include human AD cortex single-cell RNA-seq data accessed through AlzData (http://www.alzdata.org/), mouse brain single-cell RNA-seq data accessed through the Broad Institute Single Cell Portal (https://singlecell.broadinstitute.org/), and the Allen Brain Atlas mouse brain reference dataset used for cell-type label transfer in snRNA-seq annotation. STAT3 position weight matrices were retrieved from the JASPAR database (https://jaspar.genereg.net/), and STAT3 transcription factor–target gene relationships were retrieved from the hTFtarget database (http://bioinfo.life.hust.edu.cn/hTFtarget/). The mm10-2020-A mouse reference genome was used for sequencing read alignment. Antibody information, single-cell DEG and cluster marker tables, statistical details, and other supporting tabular data are provided in Supplementary Data 1–4. Source data are provided with this paper. Raw data are available on request. Source data are provided with this paper.
