# Supplementary material for: Microglial CD31 suppresses Aβ clearance and promotes Alzheimer pathology in 5×FAD mice
Source: Nat Commun. 2026 Jun 5;17:7217. doi: 10.1038/s41467-026-74037-5 (PMC13396677; doi:10.1038/s41467-026-74037-5)
Supplement: Supplementary file 1 — Supplementary Information [file 41467_2026_74037_MOESM1_ESM.pdf]

Supplementary figures and legends

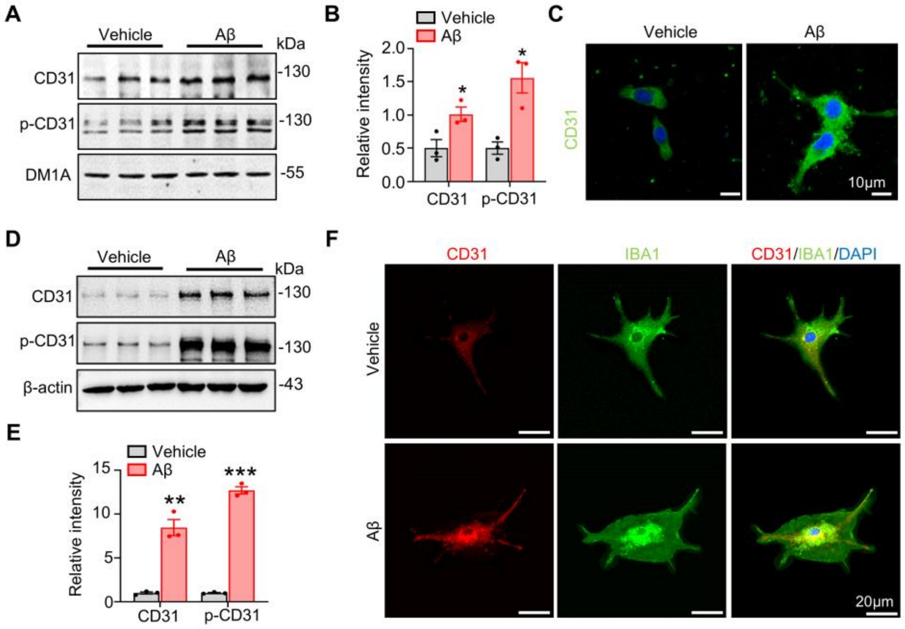

Supplementary Fig.1 Aβ oligomers increase CD31 expression levels in BV-2 and primary microglial cells

(A, B) $A\beta_{1-42}$ oligomers significantly increased CD31 expression and phosphorylation in BV-2 cells after 24 h, as detected by Western blotting. *n* = 3 independent experiments, two-sided unpaired Student's t-test, **p* = 0.039 (CD31), ***p* = 0.0126 (p-CD31) vs vehicle group. (C) Immunofluorescence staining confirmed that $A\beta_{1-42}$ oligomers promoted CD31 expression in BV-2 cells. Representative images from three independent experiments with similar results are shown. Bar = 10 μm. (D, E) $A\beta_{1-42}$ oligomers significantly elevated CD31 expression and phosphorylation levels in primary cultured microglia after 24 h treatment, as detected by Western blotting. n = 3 independent experiments, two-sided unpaired Student's t-test, ***p* = 0.0018, ****p* < 0.0001 vs vehicle group. (F) Immunofluorescence confirmed that $A\beta_{1-42}$ oligomers promoted CD31 expression in primary cultured microglial cells. Representative images from three independent experiments with similar results are shown. Bar = 20 μm. Data were presented as mean ± SEM. Source data are provided as a Source Data file.

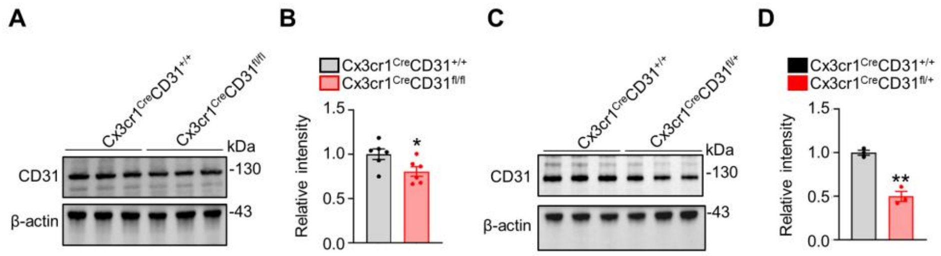

Supplementary Fig.2 CD31 expression in hippocampus of CD31 knockout mice and in primary microglia of heterozygous mice

(A, B) CD31 expression in the hippocampus of microglia-specific CD31 knockout mice (*Cx3cr1*^{Cre}CD31$^{fl/fl}$) and control mice (*Cx3cr1*^{Cre}CD31$^{+/+}$) was measured by Western blotting. Results showed that microglia-specific CD31 knockout reduced hippocampal CD31 expression by approximately 19%. n = 6 mice per group, two-sided unpaired Student's t-test, *p = 0.0396 vs *Cx3cr1*^{Cre}CD31$^{+/+}$. (C, D) CD31 expression in primary cultured microglia derived from microglia-specific CD31 heterozygous knockdown mice (*Cx3cr1*^{Cre}CD31$^{fl/+}$) and control mice (*Cx3cr1*^{Cre}CD31$^{+/+}$) was measured by Western blotting. Results revealed a significant ~47% reduction in CD31 expression in primary cultured microglia from Cx3cr1^{Cre}CD31$^{fl/+}$ mice, confirming successful CD31 knockdown in microglia. n = 3 independent experiments, two-sided unpaired Student's t-test, **p = 0.0014 vs Cx3cr1^{Cre}CD31$^{+/+}$. Data are presented as mean ± SEM. Source data are provided as a Source Data file.

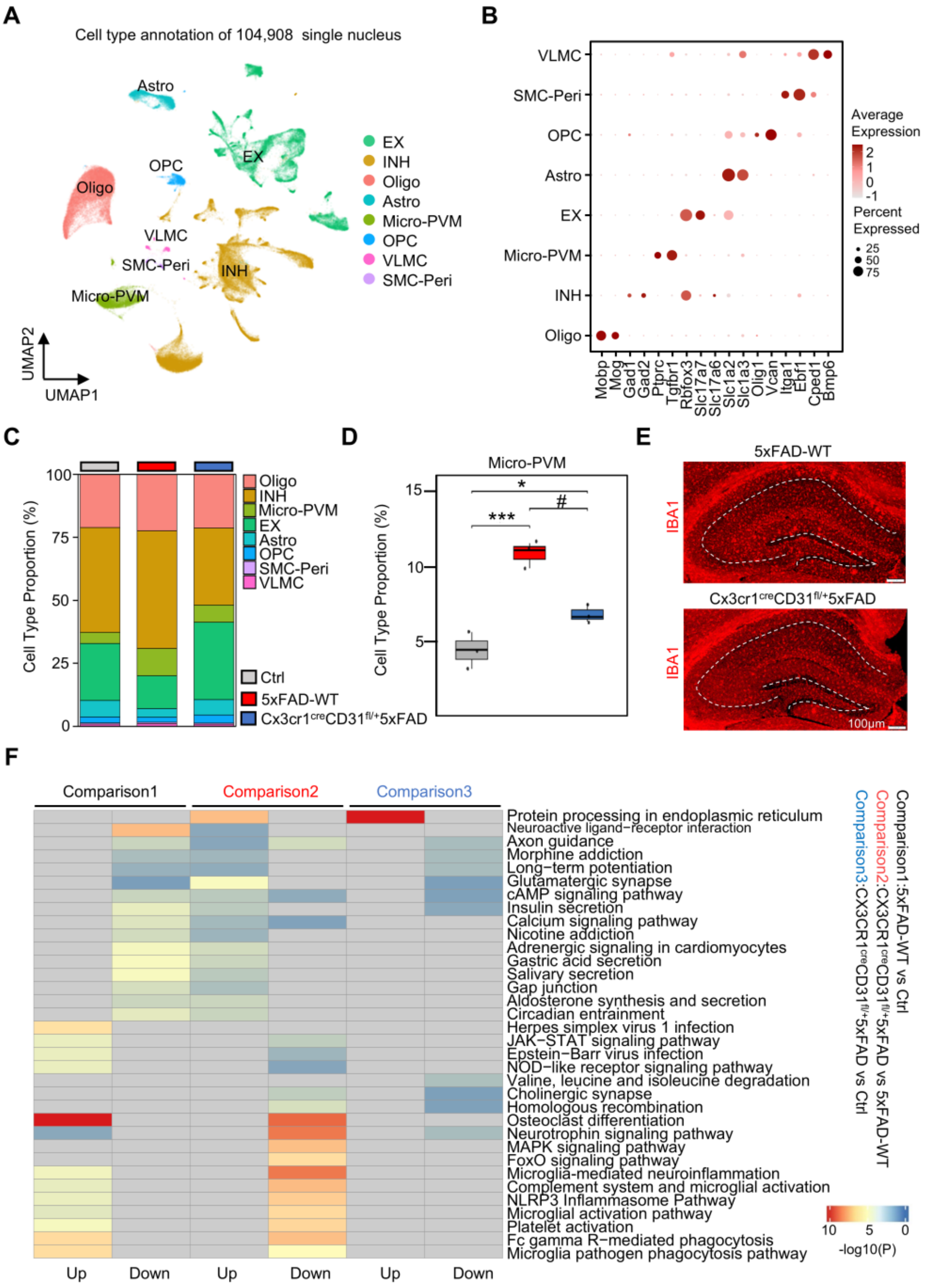

Supplementary Fig.3 Single-nucleus RNA sequencing reveals an alleviated cellular transcriptomic perturbation by CD31 knockdown in 5xFAD mice

(A) UMAP visualization of 104,908 nuclei annotated into major cell types, including excitatory neurons (EX), inhibitory neurons (INH), astrocytes (Astro), oligodendrocytes (Oligo), oligodendrocyte precursor cells (OPC), vascular leptomeningeal cells (VLMC), smooth muscle cells/pericytes (SMC-Peri), and microglia/perivascular macrophages (Micro-PVM). (B) Dot plot showing the expression levels (color intensity) and the proportion of cells expressing representative

marker genes (dot size) across cell types. (C, D) Bar plot showing the distribution of major cell types in Ctrl, 5xFAD-WT, and Cx3cr1^{Cre}CD31$^{fl/+}$5xFAD groups (C), and box plot quantifying Micro-PVM proportions (D). Micro-PVM cells were significantly increased in 5xFAD-WT mice and markedly reduced in Cx3cr1^{Cre}CD31$^{fl/+}$5xFAD mice. n = 3 mice per group, differential abundance analysis (propeller/limma with arcsine–square-root variance stabilization) with Benjamini–Hochberg FDR correction, $*p$ = 0.0430 (Ctrl vs Cx3cr1CreCD31fl/+5xFAD), ***p = 5.04×10^{-4} (Ctrl vs 5xFAD-WT), #p = 0.0138 (5xFAD-WT vs Cx3cr1CreCD31fl/+5xFAD). (E) Representative IBA1 immunofluorescence images showing reduced microglial density in the CD31-deficient group. n = 3 mice per group, Bar = 100 μm. (F) Heatmap of enriched pathways based on DEGs from all cell types across three comparisons: Comparison 1 (5xFAD-WT vs Ctrl), Comparison 2 (*Cx3cr1*^{Cre}CD31$^{fl/+}$5xFAD vs Ctrl), and Comparison 3 (*Cx3cr1*^{Cre}CD31$^{fl/+}$5xFAD vs 5xFAD-WT). The analysis indicates that many pathway alterations induced by 5xFAD were reversed by CD31 knockdown. Enrichment significance was assessed using a hypergeometric test with Benjamini–Hochberg correction for multiple comparisons. Data are presented as the mean ± SEM. Source data are provided as a Source Data file.

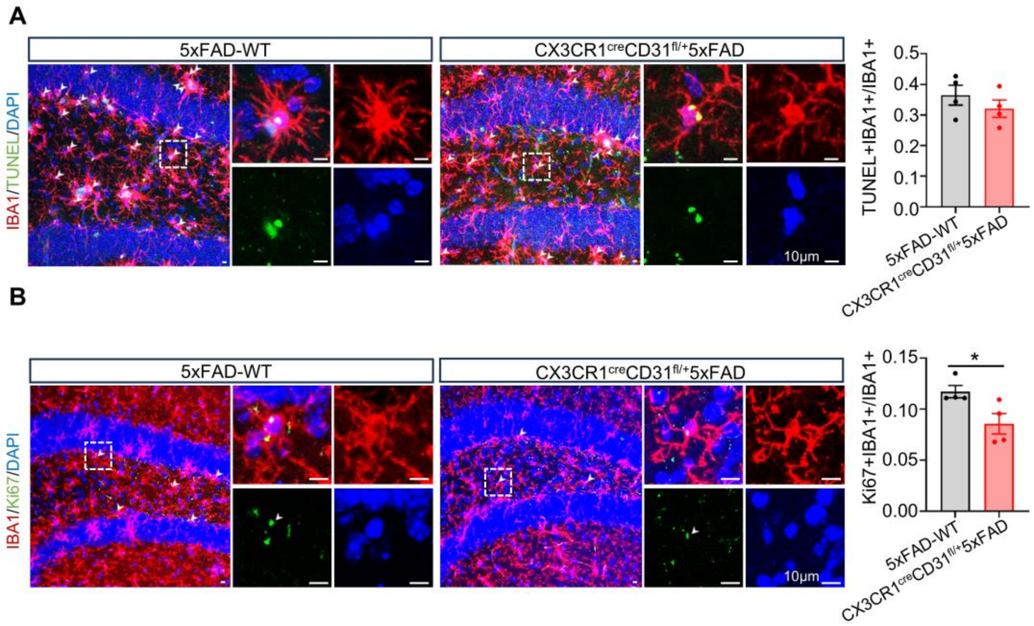

Supplementary Fig.4 CD31 knockdown reduces microglial proliferation without

affecting apoptosis in 5xFAD mice

(A) Immunofluorescence staining indicates no significant change in TUNEL+IBA1+ microglia following CD31 knockdown. n = 4 mice per group, two-sided unpaired Student's t-test, p = 0.3483 vs 5xFAD-WT. (B) Immunofluorescence staining shows a reduced proportion of Ki67+IBA1+ microglia in *Cx3cr1*^{Cre}CD31$^{fl/+}$5xFAD mice compared with 5xFAD-WT controls. two-sided unpaired Student's t-test, *n* = 4 mice per group， **p* = 0.0368 vs 5xFAD-WT, Bar = 10 μm. Data are presented as mean ± SEM. Source data are provided as a Source Data file.

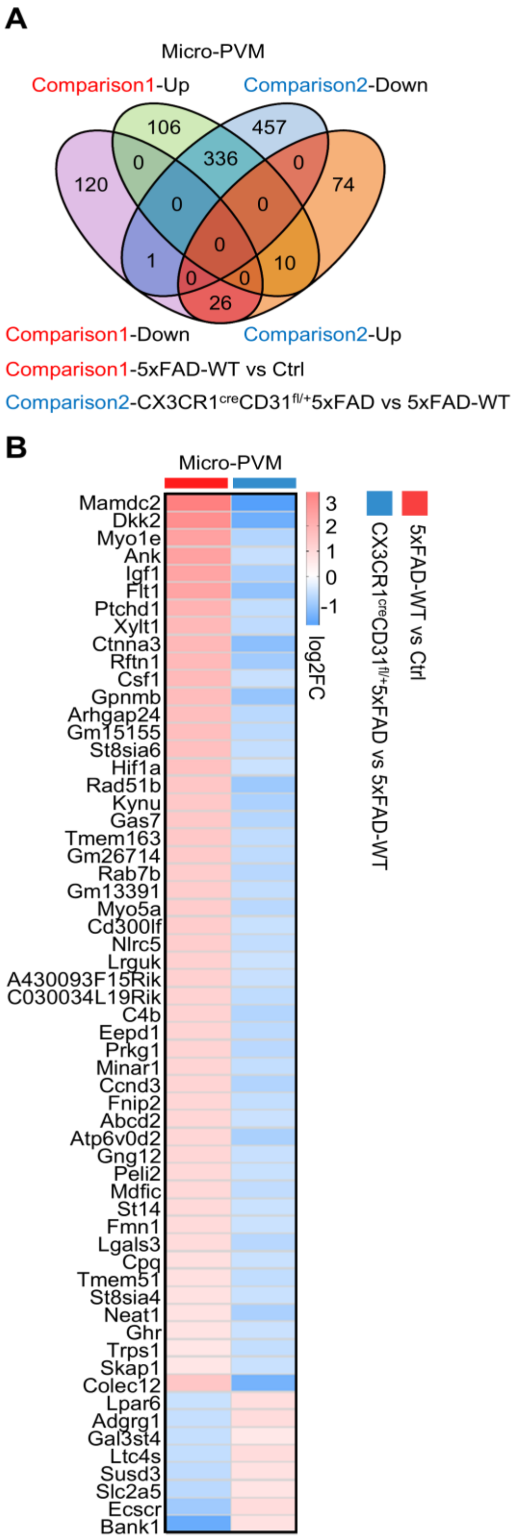

Supplementary Fig.5 CD31 knockdown reverses microglia transcriptional dysregulation in 5xFAD mice

(A) Venn diagrams show DEGs in Micro-PVM cells across two comparison groups (Comparison1: 5xFAD-WT vs Ctrl; Comparison2: *Cx3cr1*^{Cre}CD31$^{fl/+}$5xFAD vs

5xFAD-WT). A total of 362 genes exhibited reversed expression trends following CD31 knockdown in 5xFAD mice. (B) Heatmap showing log_2 fold changes derived from pseudo-bulk differential expression analysis for reversed genes that were significantly changed ($p < 0.05$, pseudo-bulk) in Micro-PVM cells. CD31 knockdown reverses transcriptional alterations observed in 5xFAD mice, suggesting its role in restoring microglial homeostasis. Source data are provided as a Source Data file.

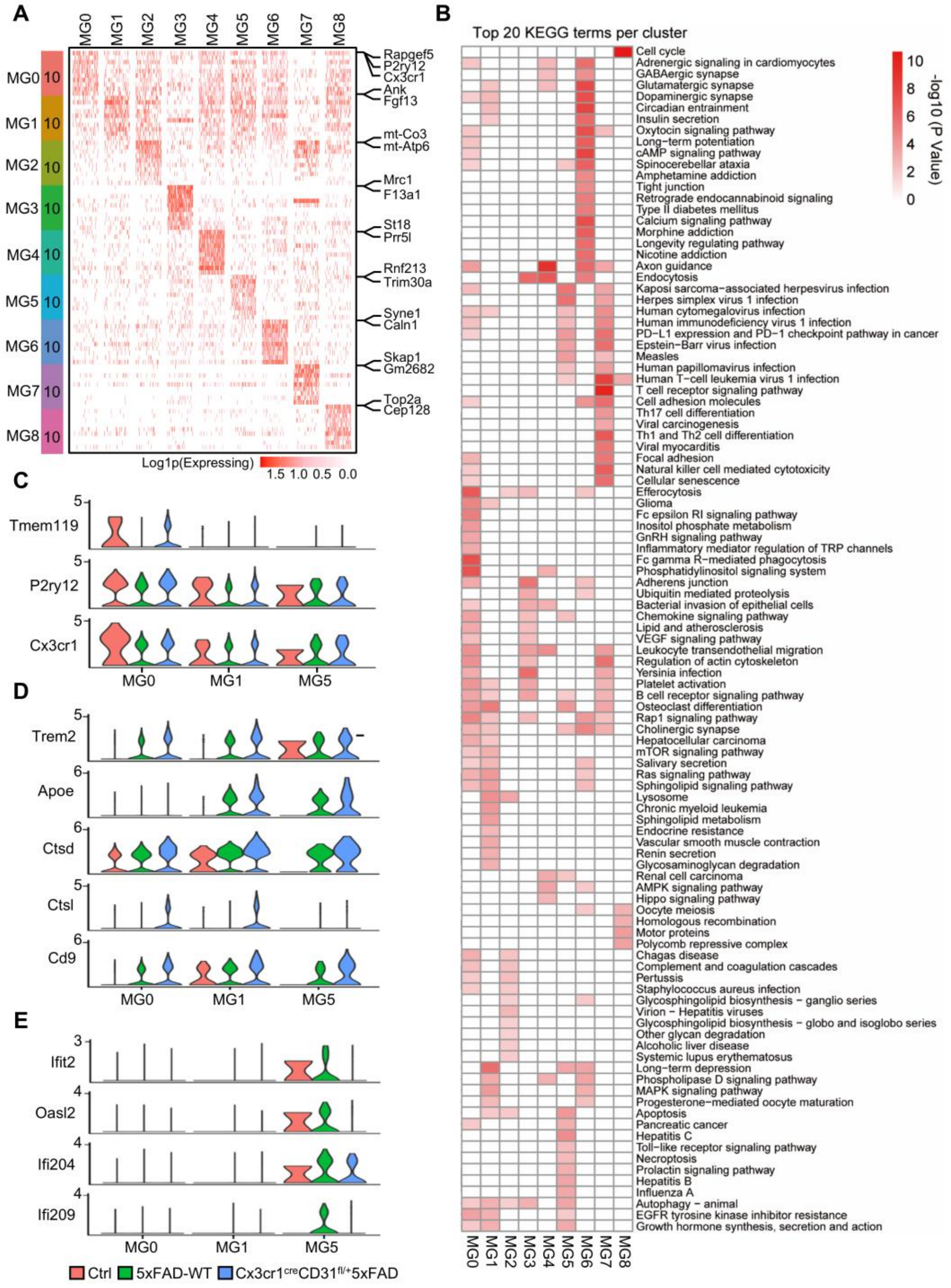

Supplementary Fig.6 Pathway enrichment and differential gene expression across microglial states

(A) Heatmap showing expression of representative marker genes across Micro-PVM subclusters. (B) Representative top 20 enriched KEGG pathways in each Micro-PVM transcriptional state. Enrichment significance was assessed using a hypergeometric test with Benjamini–Hochberg correction for multiple comparisons. (C) Expression of genes associated with homeostatic Micro-PVM identity among microglia subtypes. (D) Expression of genes involved in pro-inflammatory interferon signaling pathways among microglia subtypes. (E) Expression of genes related to phagocytic degradation and tissue repair processes among microglia subtypes. Source data are provided as a Source Data file.

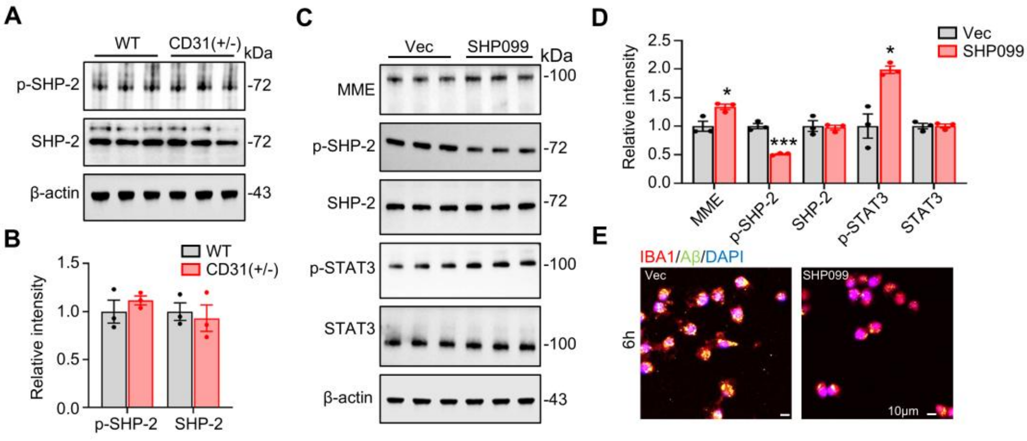

Supplementary Fig.7 SHP-2 inhibition enhances STAT3 phosphorylation and promotes Aβ degradation

(A, B) Microglia-specific CD31 knockdown did not alter SHP-2 expression or active-site phosphorylation levels, as detected by Western blotting. *n* = 3 independent experiments, two-sided unpaired Student's t-test, p = 0.7029 (SHP-2), p = 0.4135 (p-SHP-2) vs Cx3cr1CreCD31+/+. (C, D) Pharmacological inhibition of SHP-2 with SHP099 (0.07 μM, 24 h) significantly increased MME expression and STAT3 phosphorylation levels in BV2 cells measured by Western blotting. *n* = 3 independent experiments, two-sided unpaired Student's t-test, **p* = 0.0276 (MME), **p* = 0.0109 (p-STAT3), ****p* = 0.0005 (p-SHP-2) vs Vec. (E) SHP-2 inhibition with SHP099 (0.07

μM, 24 h) enhanced Aβ degradation in BV2 cells compared with vehicle group detected by immunofluorescence. *n* = 3 independent experiments, Bar = 10 μm. Data are presented as mean ± SEM. Source data are provided as a Source Data file.

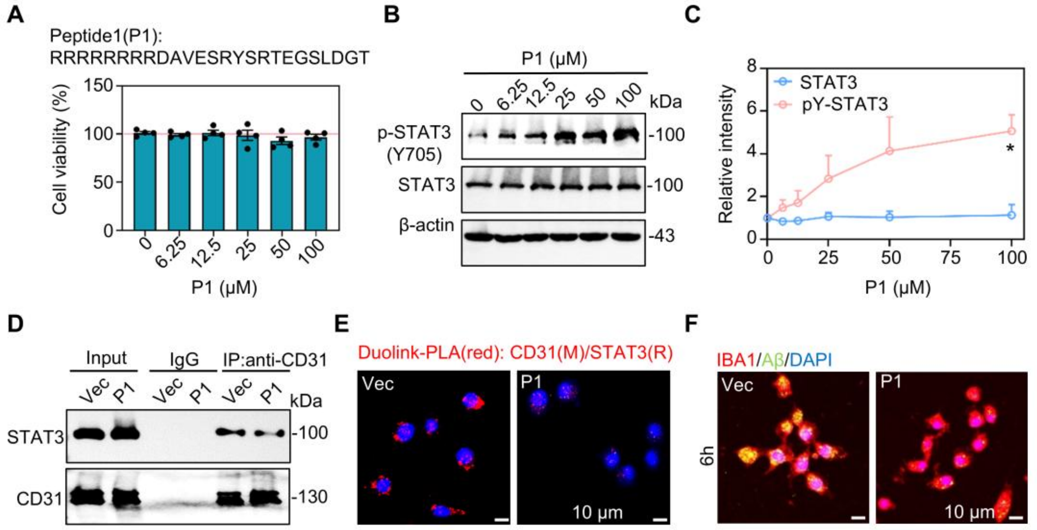

Supplementary Fig.8 A newly designed peptide (P1) reduces CD31-STAT3 binding and enhances Aβ degradation in BV-2 cells

(A) CCK8 assays performed after treating BV-2 cells for 24 h with various concentrations of peptide P1 showed no significant cytotoxicity. *n* = 4 independent experiments, one-way ANOVA with Tukey's post hoc test, $F(5,18) = 0.8999$, $p = 0.5023$. (B, C) After 24 h of treating BV-2 cells with various concentrations of P1, Western blotting showed a concentration-dependent increase in STAT3 phosphorylation. *n* = 3 independent experiments, one-way ANOVA with Tukey's post hoc test, $F(5,12) = 3.533$, overall $p = 0.0340$; 100 μM vs 0 μM adjusted $^{*}p = 0.0491$. (D) A reduction in the binding between STAT3 and CD31 was observed following treatment with P1 (100 μM) for 24 h in BV-2 cells, as demonstrated by Western blotting after immunoprecipitation with STAT3 antibody. *n* = 3 independent experiments. (E) Proximity ligation assay (PLA) showed reduced binding between STAT3 and CD31 after treating BV-2 cells with P1 (100 μM) for 24 h. *n* = 3 independent experiments. Bar = 10 μm. (F) Enhanced degradation of Aβ by BV-2 cells after treatment with P1 (100 μM) for 24 h was demonstrated by immunofluorescence. *n* = 3 independent experiments. Bar = 10 μm.

Data are presented as mean ± SEM. Source data are provided as a Source Data file.

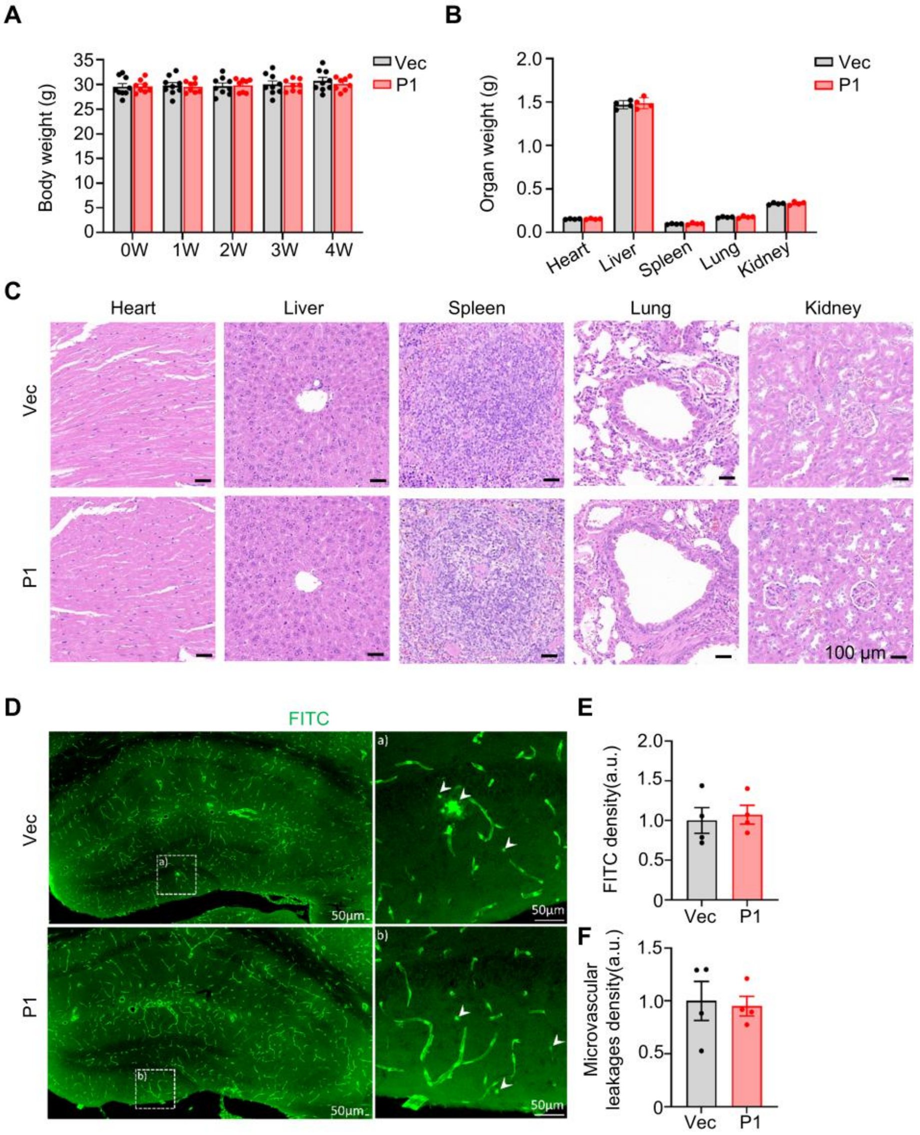

Supplementary Fig.9 Intracerebroventricular administration of P1 does not induce systemic toxicity

(A) Body weight of mice monitored over 4 weeks following intracerebroventricular administration of P1 or vehicle control (Vec). No significant differences in body weight were observed between groups, n = 8 (P1) and 9 (Vec) mice per group, two-way repeated-measures ANOVA with Geisser–Greenhouse correction and Šídák's multiple comparisons test p = 0.5057 (interaction), p = 0.0104 (time), p = 0.8488 (treatment). (B) Organ weights (heart, liver, spleen, lung, and kidney) collected at the endpoint. No significant differences were detected between Vec and P1-treated mice, n = 4 mice per

group, two-sided unpaired Student's t-test, p = 0.8960 (heart), p = 0.6745 (liver), p = 0.5882 (spleen), p = 0.7121 (lung), p = 0.7630 (kidney) vs Vec. (C) Representative H&E staining of major organs (heart, liver, spleen, lung, and kidney) from Vec and P1-treated mice. No obvious histopathological abnormalities were observed following P1 administration. *n* = 4 mice per group. Bar=100 μm. (D-F) Cerebral vascular density and microvascular permeability were assessed by FITC perfusion in 5xFAD mice treated with Vec or P1. P1 treatment did not affect vascular structure or microvascular leakage. White arrows indicate FITC leakage from microvessels into the brain parenchyma. *n* = 4 mice per group, two-sided unpaired Student's t-test, p = 0.7304 (FITC density), p = 0.8176 (microvascular leakage density) vs Vec. Bar = 50 μm. Data are presented as mean ± SEM. Source data are provided as a Source Data file.

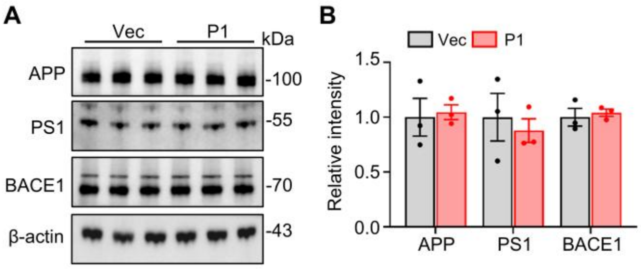

Supplementary Fig.10 P1 treatment does not affect amyloid production.

(A, B) P1 treatment did not significantly alter the expression levels of APP or the major amyloid-generating enzymes PS1 and BACE1 detected by Western blotting. *n* = 3 mice per group, two-sided unpaired Student's t-test, p = 0.8168 (APP), p = 0.6406 (PS1), p = 0.6599 (BACE1) vs Vec. Data are presented as mean ± SEM. Source data are provided as a Source Data file.

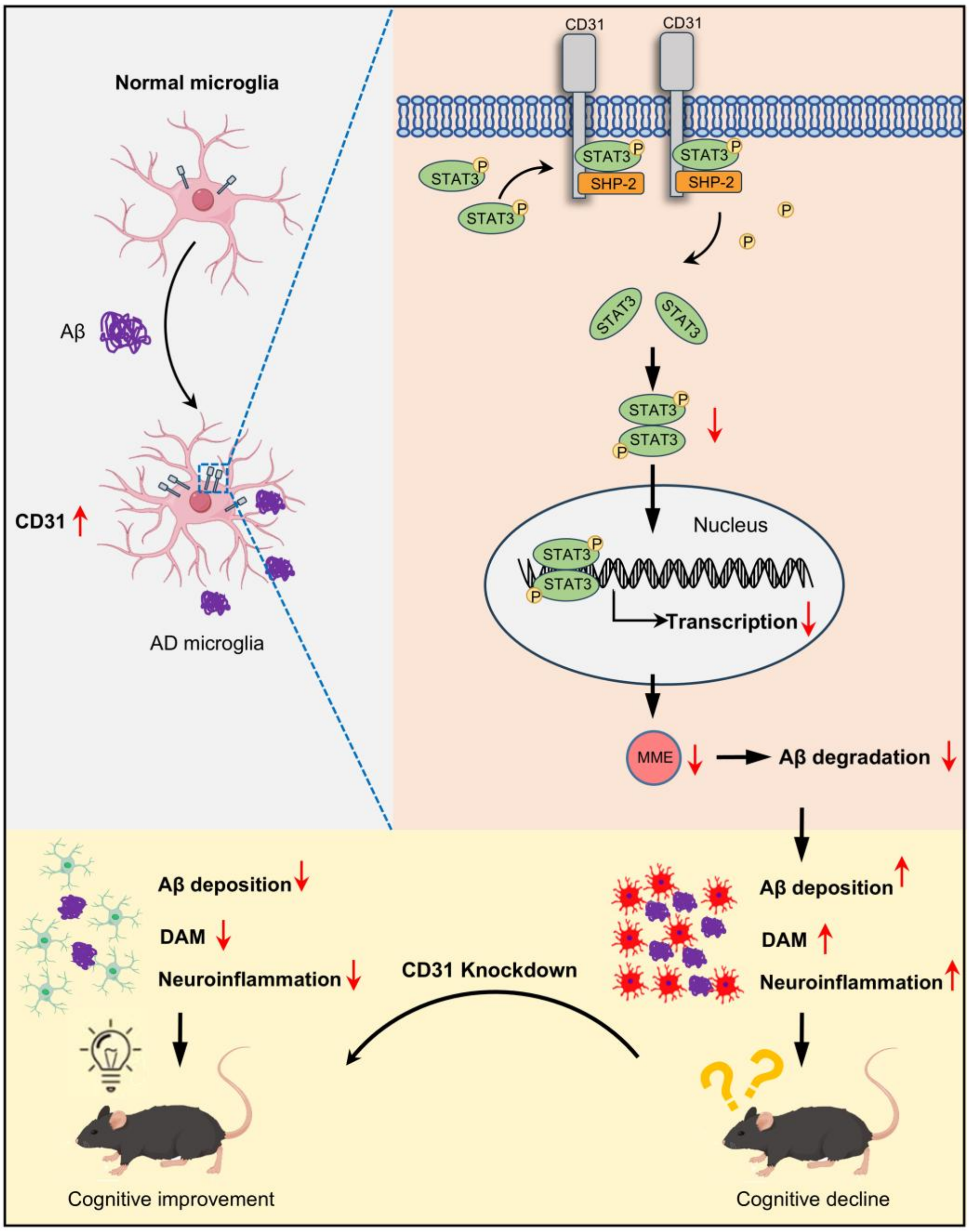

Supplementary Fig.11 Schematic diagram illustrating the mechanisms by which microglial CD31 regulates AD pathology

In Alzheimer's disease, elevated microglial CD31 promotes microglial hyperactivation and Aβ deposition via simultaneous orchestrating SHP2 and STAT3 to promote STAT3 dephosphorylation/inactivation and reduce MME transcription, leading to Aβ accumulation and ultimately cognitive impairment. Conversely, microglial CD31 knockdown reverses these pathological changes by modulating the CD31/SHP2/STAT3/MME pathway, leading to reduced Aβ accumulation, decreased disease-associated microglia (DAM), mitigated neuroinflammation, and ultimately

improves cognitive function. Certain illustrative components are created by Figdraw (ID: ISIWTc707c).
