# Supplementary material for: Microglial CD31 suppresses Aβ clearance and promotes Alzheimer pathology in 5×FAD mice
Source: Nat Commun. 2026 Jun 5;17:7217. doi: 10.1038/s41467-026-74037-5 (PMC13396677; doi:10.1038/s41467-026-74037-5)
Supplement: Supplementary file 2 — Description of Additional Supplementary Files [file 41467_2026_74037_MOESM2_ESM.pdf]

Description of Additional Supplementary Files

Supplementary Data 1. Antibodies used in this study.
A list of primary antibodies, including dilution, application, and source information, is provided. WB, Western blotting; IF, immunofluorescence; IHC, immunohistochemistry; ChIP, chromatin immunoprecipitation.

Supplementary Data 2. Statistical reporting summary for all quantitative analyses.
Statistical parameters for each figure panel are provided, including outcome measures, group comparisons, statistical tests, test statistics (t or F values), exact P values, post hoc analyses, and assumption checks where applicable.

Supplementary Data 3. Differentially expressed genes and associated statistical values from transcriptomic analyses.
Differentially expressed genes for the indicated analyses are listed with log2 fold changes and P values. Statistical significance was determined using the Wilcoxon rank-sum test (two-sided). Nominal P values are reported, and adjusted P values (Benjamini–Hochberg correction) are provided where applicable.

Supplementary Data 4. Marker genes used for microglial subcluster annotation.
Representative marker genes for microglial subclusters MG0–MG8 are listed. This table is descriptive and does not present additional statistical testing.
