# Supplementary material for: Microglial CD31 suppresses Aβ clearance and promotes Alzheimer pathology in 5×FAD mice
Source: Nat Commun. 2026 Jun 5;17:7217. doi: 10.1038/s41467-026-74037-5 (PMC13396677; doi:10.1038/s41467-026-74037-5)
Supplement: Supplementary file 3 — Supplementary Data 1 [file 41467_2026_74037_MOESM3_ESM.pdf]

Supplementary Data 1. Antibodies employed in this study

Antibody	Dilution	Application	Company (catalog number)
CD31	1:2000	WB	Cell Signaling (77699)
	1:100	IF	
p-CD31(Y713)	1:1000	WB	Abcam (ab180175)
Synaptophysin	1:1000	WB	Abcam (ab32127)
Synaptotagmin	1:1000	WB	Abcam (ab13259)
PSD95	1:1000	WB	Abcam (ab238135)
GluN2A	1:1000	WB	Cell Signaling (4205)
GluN2B	1:1000	WB	Cell Signaling (14544)
IL-1β	1:500	WB	ABclonal (A16288)
IL-6	1:1000	WB	ABclonal (A0286)
IL-10	1:500	WB	ABclonal (A2171)
CD80	1:500	WB	Proteintech (66406-1-Ig)
CD206	1:500	WB	Proteintech (18704-1-AP)
	1:100	IF	
CD10 (MME)	1:500	WB	Santa Cruz (sc-46656)
	1:100	IF	
JAK1	1:1000	WB	Abcam (ab133666)
p-JAK1 (Y1034+Y1035)	1:1000	WB	Abcam (ab138005)
JAK2	1:1000	WB	Abcam (ab108596)
p-JAK2 (Y1007+Y1008)	1:1000	WB	Cell Signaling (3771)
SHP2	1:500	WB	Santa Cruz (sc-7384)
P-SHP2	1： 1000	WB	Proteintech (81219-2-RR)
GFP	1:1000	WB	ABclonal (AE011)
Flag	1:1000	WB	ABclonal (AE005)
STAT3	1:1000	WB	Abcam (ab68153)
	1:50	CHIP	
p-STAT3 (Y705)	1:1000	WB	Cell Signaling (9145)
	1:100	IF	
APP	1:1000	WB	Zen-Bioscience (R22718)
PS1	1:1000	WB	ABclonal (A19103)
BACE1	1:1000	WB	ABclonal (A11533)
β-actin	1:2000	WB	ABclonal (AC026)
IBA1	1:500	IF	Wako (019-19741)
GFAP	1:500	IF	Abcam (ab279289)
Ki67	1:500	IF	Abcam (ab15580)
Alexa Fluor™ 488	1:500	IF	Thermo Fisher (A-21206)
Alexa Fluor™ 546	1:500	IF	Thermo Fisher (A-11003)

WB: Western blotting; IF: immunofluorescence; IHC: immunochemistry; CHIP: chromatin immunoprecipitation
