# Supplementary material for: Microglial CD31 suppresses Aβ clearance and promotes Alzheimer pathology in 5×FAD mice
Source: Nat Commun. 2026 Jun 5;17:7217. doi: 10.1038/s41467-026-74037-5 (PMC13396677; doi:10.1038/s41467-026-74037-5)
Supplement: Supplementary file 7 — Reporting Summary [file 41467_2026_74037_MOESM7_ESM.pdf]

nature portfolio

Corresponding author(s): Jian-Zhi Wang

Last updated by author(s): Mar 3, 2026

Reporting Summary

Nature Portfolio wishes to improve the reproducibility of the work that we publish. This form provides structure for consistency and transparency in reporting. For further information on Nature Portfolio policies, see our Editorial Policies and the Editorial Policy Checklist.

Statistics

For all statistical analyses, confirm that the following items are present in the figure legend, table legend, main text, or Methods section.

n/a	Confirmed	
☐	☒	The exact sample size (*n*) for each experimental group/condition, given as a discrete number and unit of measurement
☐	☒	A statement on whether measurements were taken from distinct samples or whether the same sample was measured repeatedly
☐	☒	The statistical test(s) used AND whether they are one- or two-sided<br>*Only common tests should be described solely by name; describe more complex techniques in the Methods section.*
☒	☐	A description of all covariates tested
☐	☒	A description of any assumptions or corrections, such as tests of normality and adjustment for multiple comparisons
☐	☒	A full description of the statistical parameters including central tendency (e.g. means) or other basic estimates (e.g. regression coefficient) AND variation (e.g. standard deviation) or associated estimates of uncertainty (e.g. confidence intervals)
☐	☒	For null hypothesis testing, the test statistic (e.g. *F*, *t*, *r*) with confidence intervals, effect sizes, degrees of freedom and *P* value noted<br>*Give P values as exact values whenever suitable.*
☒	☐	For Bayesian analysis, information on the choice of priors and Markov chain Monte Carlo settings
☐	☒	For hierarchical and complex designs, identification of the appropriate level for tests and full reporting of outcomes
☒	☐	Estimates of effect sizes (e.g. Cohen's *d*, Pearson's *r*), indicating how they were calculated

Our web collection on statistics for biologists contains articles on many of the points above.

Software and code

Policy information about availability of computer code

Data collection	Mouse behavioral data: video tracking system TM-Vision (LAB-0004-0006-CDTM) from Chengdu Taimeng Software Co., Ltd, China.<br>Immunofluorescence and confocal imaging: two-photon confocal microscope LSM800 (Zeiss) operated with ZEN software (2012, black edition 8.0.0.273).<br>Golgi-Cox staining whole-slide imaging: Pannoramic SCAN II (3DHISTECH).<br>Single-nucleus RNA-seq library preparation: Chromium Single Cell 3′ Reagent Kit v3 (10x Genomics, PN-1000075) on the Chromium Controller (10x Genomics).<br>Library QC: Qubit dsDNA HS assay (Thermo Fisher) and Agilent Bioanalyzer High Sensitivity chip (Agilent).<br>snRNA-seq and bulk RNA-seq: Illumina NovaSeq 6000 sequencing platform (Illumina).<br>Quantitative real-time PCR: StepOnePlus Real-Time PCR Detection System (272001262, AB Applied Biosystems) with StepOne Software (v2.3).<br>Aβ1-40 / Aβ1-42 detection: BioTek (Synergy H1) with Gen5 software (v2.09).<br>Western blot imaging: ECL chemiluminescence detection system ClinX (ChemiScope 6000).
Data analysis	GraphPad Prism (v10.1.2): Statistical analysis and graphical representations.<br>ImageJ (Fiji v1.52h): Image processing and analysis.<br>fastp (v0.20.0): snRNA-seq raw read quality trimming.<br>Cell Ranger (v7.1.0, 10x Genomics): snRNA-seq alignment and quantification against mm10-2020-A mouse reference genome.<br>R (v4.4.0) with Seurat (v5.3.0): snRNA-seq downstream analysis including preprocessing, normalization, integration (CCA), clustering, dimensionality reduction, cluster-level differential expression, and visualization.<br>Seurat (v5.3.0) FindTransferAnchors / TransferData: Reference-based cell-type annotation against the Allen Brain Atlas reference dataset.<br>DoubletFinder (v2.0.3): Doublet detection and removal.

Harmony (v1.2.3): Batch-effect correction.
DESeq2 (v1.44.0): Pseudobulk differential expression analysis.
limma (v3.60.6, arcsine-square-root transformed framework adapted from propeller (Phipson et al., 2022)): Differential abundance analysis of cell population proportions.
clusterProfiler (v4.12.6): Pathway enrichment analysis for GO biological processes and KEGG pathways, with Benjamini–Hochberg correction for multiple testing.
dplyr (v1.1.4), tidyr (v1.3.1): General data manipulation and tidying.
ggplot2 (v4.0.0), ggnewscale (v0.5.2), RColorBrewer (v1.1.3), scCustomize (v3.0.1), ggrepel (v0.9.6), scales (v1.4.0): Data visualization and figure generation.

For manuscripts utilizing custom algorithms or software that are central to the research but not yet described in published literature, software must be made available to editors and reviewers. We strongly encourage code deposition in a community repository (e.g. GitHub). See the Nature Portfolio guidelines for submitting code & software for further information.

Data

Policy information about availability of data

All manuscripts must include a data availability statement. This statement should provide the following information, where applicable:

- Accession codes, unique identifiers, or web links for publicly available datasets
- A description of any restrictions on data availability
- For clinical datasets or third party data, please ensure that the statement adheres to our policy

Raw sequencing data generated in this study have been deposited in the National Genomics Data Center (NGDC) Genome Sequence Archive (GSA) and are publicly available under accession codes CRA028924 (snRNA-seq; https://ngdc.cncb.ac.cn/gsa/browse/CRA028924) and CRA028867 (bulk RNA-seq; https://ngdc.cncb.ac.cn/gsa/browse/CRA028867). Publicly available datasets reanalyzed in this study include human AD cortex single-cell RNA-seq data accessed through AlzData (http://www.alzdata.org/), mouse brain single-cell RNA-seq data accessed through the Broad Institute Single Cell Portal (https://singlecell.broadinstitute.org/), and the Allen Brain Atlas mouse brain reference dataset used for cell-type label transfer in snRNA-seq annotation. STAT3 position weight matrices were retrieved from the JASPAR database (https://jaspar.genereg.net/), and STAT3 transcription factor target gene relationships were retrieved from the hTFtarget database (http://bioinfo.life.hust.edu.cn/hTFtarget/). The mm10-2020-A mouse reference genome was used for sequencing read alignment. Source data underlying all graphs and statistical comparisons in the figures are provided as a Source Data file accompanying this paper. Antibody information, single-cell DEG and cluster marker tables, statistical details, and other supporting tabular data are provided in Supplementary Tables 1 to 4.

Research involving human participants, their data, or biological material

Policy information about studies with human participants or human data. See also policy information about sex, gender (identity/presentation), and sexual orientation and race, ethnicity and racism.

Reporting on sex and gender	Did not involve in this study.
Reporting on race, ethnicity, or other socially relevant groupings	Did not involve in this study.
Population characteristics	Did not involve in this study.
Recruitment	Did not involve in this study.
Ethics oversight	Did not involve in this study.

Note that full information on the approval of the study protocol must also be provided in the manuscript.

Field-specific reporting

Please select the one below that is the best fit for your research. If you are not sure, read the appropriate sections before making your selection.

☒ Life sciences ☐ Behavioural & social sciences ☐ Ecological, evolutionary & environmental sciences

For a reference copy of the document with all sections, see nature.com/documents/nr-reporting-summary-flat.pdf

Life sciences study design

All studies must disclose on these points even when the disclosure is negative.

Sample size	No statistical method was used to predetermine sample size. Sample sizes for each experiment are reported in the corresponding figure legends. They were chosen based on sample sizes commonly used in the field of microglia and Alzheimer's disease research for analogous experimental paradigms, including studies that combine 5xFAD or related AD mouse models with behavioral assessment, single-nucleus RNA sequencing, primary microglial cultures, and biochemical or histological analyses (Zhang et al., Nat Commun 2017, PMID: 28345579; Ledo et al., Mol Psychiatry 2021, PMID: 32792660; Wang et al., Signal Transduct Target Ther 2024, PMID: 38679634), and on our empirical experience that takes into account the biological variability of each readout, with relatively larger group sizes used for behavioral tests given their higher inter-animal variability. The chosen sample sizes were sufficient to detect the reported effects with statistical significance, and key findings were independently reproduced across multiple biological replicates.
Data exclusions	No data were excluded from any of the experiments in this study, except for standard quality-control filtering applied to the bulk RNA

	sequencing and single-nucleus RNA sequencing data, as described in the methods.
Replication	Each experiment was independently repeated at least three times by different researchers to ensure the reproducibility of the findings. All experimental findings were successfully replicated.
Randomization	Animals and cells were randomly allocated to experimental groups whenever possible. For experiments involving genetically defined comparisons, mice were grouped according to genotype, with littermate controls matched for age, sex, and genetic background to minimize confounding factors.
Blinding	Investigators were blinded to group allocation during behavioral tests and quantitative imaging analyses. For all other experiments, blinding was not feasible because group identity was either intrinsic to sample processing or required for downstream comparisons; in these cases, data were quantified using objective, predefined criteria and instrument-based readouts to minimize observer bias.

Reporting for specific materials, systems and methods

We require information from authors about some types of materials, experimental systems and methods used in many studies. Here, indicate whether each material, system or method listed is relevant to your study. If you are not sure if a list item applies to your research, read the appropriate section before selecting a response.

Materials & experimental systems

n/a	Involved in the study	
	x	Antibodies
	x	Eukaryotic cell lines
x		Palaeontology and archaeology
	x	Animals and other organisms
x		Clinical data
x		Dual use research of concern
x		Plants

Methods

n/a	Involved in the study	
x		ChIP-seq
x		Flow cytometry
x		MRI-based neuroimaging

Antibodies

Antibodies used

1. CD31/PECAM-1 (D8V9E) Rabbit mAb, Cell Signaling, Cat# 77699, WB 1:2000; IF 1:100, https://www.cellsignal.com/products/primary-antibodies/cd31-pecam-1-d8v9e-rabbit-monoclonal-antibody/77699
2. CD31 phospho Y713 antibody [EPR8079(2)] - C-terminal, Abcam, Cat# ab180175, WB 1:1000, https://www.abcam.com/en-us/products/primary-antibodies/cd31-phospho-y713-antibody-epr80792-c-terminal-ab180175
3. Synaptophysin antibody [YE269] - Synaptic Marker, Abcam, Cat# ab32127, WB 1:1000, https://www.abcam.com/en-us/products/primary-antibodies/synaptophysin-antibody-ye269-synaptic-marker-ab32127
4. Synaptotagmin antibody [ASV30], Abcam, Cat# ab13259, WB 1:1000, https://www.abcam.com/en-us/products/primary-antibodies/synaptotagmin-antibody-asv30-ab13259
5. PSD95 antibody [EPR23124-118] - Synaptic Marker, Abcam, Cat# ab238135, WB 1:1000, https://www.abcam.com/en-us/products/primary-antibodies/psd95-antibody-epr23124-118-synaptic-marker-ab238135
6. NMDA Receptor 2A/GluN2A Antibody, Cell Signaling, Cat# 4205, WB 1:1000, https://www.cellsignal.com/products/primary-antibodies/nmda-receptor-2a-glun2a-antibody/4205
7. NMDA Receptor 2B/GluN2B (D8E10) Rabbit mAb, Cell Signaling, Cat# 14544, WB 1:1000, https://www.cellsignal.com/products/primary-antibodies/nmda-receptor-2b-glun2b-d8e10-rabbit-monoclonal-antibody/14544
8. IL-1β Rabbit pAb, ABclonal, Cat# A16288, WB 1:500, https://abclonal.com/catalog-antibodies/IL1RabbitpAb/A16288
9. IL-6 Rabbit pAb, ABclonal, Cat# A0286, WB 1:1000, https://abclonal.com/catalog-antibodies/IL6RabbitpAb/A0286
10. IL-10 Rabbit pAb, ABclonal, Cat# A2171, WB 1:500, https://abclonal.com/catalog-antibodies/IL10RabbitpAb/A2171
11. CD80/B7-1 Monoclonal antibody, Proteintech, Cat# 66406-1-Ig, WB 1:500, https://www.ptglab.com/products/B7-1-Antibody-66406-1-Ig.htm
12. CD206/MRC1 Polyclonal antibody, Proteintech, Cat# 18704-1-AP, WB 1:500; IF 1:100, https://www.ptglab.com/products/MRC1-Antibody-18704-1-AP.htm
13. CD10 Antibody (F-4), Santa Cruz Biotechnology, Cat# sc-46656, WB 1:500; IF 1:100, https://www.scbt.com/p/cd10-antibody-f-4
14. JAK1 antibody [EPR349(N)], Abcam, Cat# ab133666, WB 1:1000, https://www.abcam.com/en-us/products/primary-antibodies/jak1-antibody-epr349n-ab133666
15. JAK1 phospho Y1034 + Y1035 antibody [EPR1899(2)], Abcam, Cat# ab138005, WB 1:1000, https://www.abcam.com/en-us/products/primary-antibodies/jak1-phospho-y1034-y1035-antibody-epr18992-ab138005
16. JAK2 antibody [EPR108(2)], Abcam, Cat# ab108596, WB 1:1000, https://www.abcam.com/en-us/products/primary-antibodies/jak2-antibody-epr1082-ab108596
17. Phospho-Jak2 (Tyr1007/1008) Antibody, Cell Signaling, Cat# 3771, WB 1:1000, https://www.cellsignal.cn/products/primary-antibodies/phospho-jak2-tyr1007-1008-antibody/3771
18. SH-PTP2 Antibody (B-1), Santa Cruz Biotechnology, Cat# sc-7384, WB 1:500, https://www.scbt.com/p/sh-ptp2-antibody-b-1
19. Phospho-SHP2 (Tyr580) Antibody, Proteintech, Cat# 81219-2-RR, WB 1:1000, https://www.ptglab.com/products/Phospho-SHP2-Tyr580-Antibody-81219-2-RR.htm
20. GFP-Tag Rabbit pAb, ABclonal, Cat# AE011, WB 1:1000, https://abclonal.com/catalog-antibodies/RabbitantiGFPTagpAb/AE011
21. DDDDK-Tag Mouse mAb, ABclonal, Cat# AE005, WB 1:1000, https://abclonal.com/catalog-antibodies/MouseantiDDDDKTagmAb/AE005

22. STAT3 antibody [EPR787Y], Abcam, Cat# ab68153, WB 1:1000; ChIP 1:50, https://www.abcam.com/en-us/products/primary-antibodies/stat3-antibody-epr787y-ab68153
23. Phospho-STAT3 (Tyr705) (D3A7) Rabbit mAb, Cell Signaling, Cat# 9145, WB 1:1000; IF 1:100, https://www.cellsignal.com/products/primary-antibodies/phospho-stat3-tyr705-d3a7-rabbit-monoclonal-antibody/9145
24. APP antibody, Zen-Bioscience, Cat# R22718, WB 1:1000, http://www.zen-bioscience.com/goodsDetail?productNo=R22718
25. Presenilin 1 Rabbit mAb, ABclonal, Cat# A19103, WB 1:1000, https://abclonal.com/catalog-antibodies/Presenilin1RabbitmAb/A19103
26. BACE1 Rabbit mAb, ABclonal, Cat# A11533, WB 1:1000, https://abclonal.com/catalog-antibodies/BACE1RabbitmAb/A11533
27. β-Actin Rabbit mAb - High Dilution, ABclonal, Cat# AC026, WB 1:2000, https://abclonal.com/catalog-antibodies/ActinRabbitmAbHighDilution/AC026
28. Anti-Iba1, Rabbit, for Immunocytochemistry, FUJIFILM Wako, Cat# 019-19741, IF 1:500, https://labchem-wako.fujifilm.com/us/product/detail/W01W0101-1974.html
29. GFAP antibody [EPR1034Y] - Astrocyte Marker, mouse IgG1 chimeric, Abcam, Cat# ab279289, IF 1:500, https://www.abcam.com/en-us/products/primary-antibodies/gfap-antibody-epr1034y-astrocyte-marker-mouse-igg1-chimeric-ab279289
30. Ki67 antibody, Abcam, Cat# ab15580, IF 1:500, https://www.abcam.com/en-us/products/primary-antibodies/ki67-antibody-ab15580
31. Donkey anti-Rabbit IgG (H+L) Highly Cross-Adsorbed Secondary Antibody, Alexa Fluor 488, Thermo Fisher, Cat# A-21206, IF 1:500, https://www.thermofisher.com/antibody/product/Donkey-anti-Rabbit-IgG-H-L-Highly-Cross-Adsorbed-Secondary-Antibody-Polyclonal/A-21206
32. Goat anti-Mouse IgG (H+L) Cross-Adsorbed Secondary Antibody, Alexa Fluor 546, Thermo Fisher, Cat# A-11003, IF 1:500, https://www.thermofisher.com/antibody/product/Goat-anti-Mouse-IgG-H-L-Cross-Adsorbed-Secondary-Antibody-Polyclonal/A-11003

Validation	All antibodies are commercially available and have been validated by the manufacturers, as well as validated presently by different experimenters in our team or previously by different labs in our institute.

Eukaryotic cell lines

Policy information about cell lines and Sex and Gender in Research

Cell line source(s)	HEK293 and BV-2 cell lines were maintained as continuous lines in our laboratory. Primary microglia, astrocytes, and neurons were isolated from neonatal mouse pups or embryos (mixed sex).
Authentication	HEK293 cells were authenticated by STR profiling (20 STR loci plus Amelogenin), showing a 98.31% match to the HEK293 reference profile in the Cellosaurus database. BV-2 cells were not authenticated.
Mycoplasma contamination	All cell lines were routinely tested for mycoplasma contamination and were confirmed to be negative.
Commonly misidentified lines (See ICLAC register)	HEK293 is listed on the ICLAC Register of Misidentified Cell Lines. It was used solely as a heterologous expression system for transient transfection–based assays, where conclusions do not depend on its tissue origin.

Animals and other research organisms

Policy information about studies involving animals; ARRIVE guidelines recommended for reporting animal research, and Sex and Gender in Research

Laboratory animals	Wild-type C57BL/6J mice (RRID: IMSR_JAX:000664) were purchased from Beijing Vital River Laboratory Animal Technology Co., Ltd. 5×FAD transgenic mice [B6.Cg-Tg(APPSwFlLon,PSEN1*M146L*L286V)6799Vas/Mmjax; RRID: MMRRC_034848-JAX] were obtained from Shulaibao (Wuhan) Biotechnology Co., Ltd. and maintained on a C57BL/6J background. Cx3cr1-Cre mice [B6J.B6N(Cg)-Cx3cr1tm1.1(cre)Jung/J; RRID: IMSR_JAX:025524], a constitutive Cre line, were obtained from Cyagen Biosciences (project ID C001032). Pecam1 (CD31) conditional knockout mice [C57BL/6N-Pecam1em1Cyagen; serial number CKOCMP-18613-Pecam1; contract number CKOAI191104XW1-B] were custom-generated by Cyagen Biosciences on a C57BL/6N background using CRISPR/Cas9-mediated loxP insertion flanking exons 8 to 11 of the Pecam1 gene. To generate AD model mice with microglia-specific CD31 knockdown, Cx3cr1-Cre;CD31fl/fl mice were crossed with 5×FAD mice to obtain Cx3cr1Cre;CD31fl/+;5×FAD heterozygous offspring, which were used for all subsequent experiments. Adult male mice (6 months old at the time of experiments unless otherwise noted) were used in this study; both male and female mice (2 males and 1 female per group) were used for snRNA-seq. Mice were housed under specific pathogen-free conditions with a 12 h light/dark cycle, ambient temperature of 24 ± 2 °C, relative humidity of 40–70%, and ad libitum access to food and water. Because the floxed Pecam1 allele was generated on a C57BL/6N background while the other strains were maintained on a C57BL/6J background, experimental cohorts were on a mixed C57BL/6J × C57BL/6N background; to control for residual background variation and cage effects, all comparisons were performed using co-housed, age- and sex-matched littermate controls obtained from the same crosses.
Wild animals	This study did not involve the use of wild animals.
Reporting on sex	Single-nucleus RNA sequencing used a mix of male and female mice, and in vitro primary cell cultures used neonatal pups or embryos of both sexes. All other in vivo experiments exclusively used male mice.
Field-collected samples	This study did not involve samples collected from the field.
Ethics oversight	All animal experiments were approved by the Animal Care and Use Committee of Huazhong University of Science and Technology ([2022] IACUC 2751).

Note that full information on the approval of the study protocol must also be provided in the manuscript.

Plants

Seed stocks	Did not involve in this study.
Novel plant genotypes	Did not involve in this study.
Authentication	Did not involve in this study.
