# Supplementary material for: Microglial CD31 suppresses Aβ clearance and promotes Alzheimer pathology in 5×FAD mice
Source: Nat Commun. 2026 Jun 5;17:7217. doi: 10.1038/s41467-026-74037-5 (PMC13396677; doi:10.1038/s41467-026-74037-5)
Supplement: Supplementary file 8 — Transparent Peer Review File [file 41467_2026_74037_MOESM8_ESM.pdf]

nature portfolio

Peer Review File

Microglial CD31 Suppresses Aβ Clearance and Promotes Alzheimer Pathology in 5×FAD Mice

Corresponding Author: Professor Jian-Zhi Wang

This file contains all reviewer reports in order by version, followed by all author rebuttals in order by version.

Version 0:

Reviewer comments:

Reviewer #1

(Remarks to the Author)
The manuscript by Zhou et al investigated the role of microglia CD31 in AD and identified the SHP2–STA T3–MME axis For regulating AD pathogenesis. In this study, authors took the advantage of available single sequencing data and showed the elevated expression of CD31 in AD microglia. Authors then generated CD31f/fl mouse line and crossed with Cx3cr1-Cre driver mice to delete CD31 either in the WT or 5xFAD mouse background for comparative studies, which include experiments to examine the cognitive behaviors such open filed, fear conditioning, novel objective and Morris later maze, amyloid pathological changes, Abeta levels, single nuclear Seq profiling, and further mechanistic explorations. The entire manuscript has a very comprehensive set of experimental results that are presented in a step by step and logically developed manner. In my evaluation, results are supportive to their conclusion that targeted deletion of CD31, even partially, is sufficient to decrease amyloid pathology through mitigating microglia activation and restoring more homeostatic microglia. The experimental design is rigor. RNAseq data analysis is highly informative for helping with the mechanistic studies. The results for supporting the STAT3-MME pathway are striking and convincing. The most exciting finding is the identification of P1 Peptide for blocking CD31 to phosphorylate STAT3 and reduce Amyloid plaques in 5xFAD mice. The manuscript is mostly written in a logical style, and the conclusion is novel and important for the field, especially considering its potential translational value. IMO, this study should be published with no long delay. To strengthen this study further, I have a few suggestions to authors in this manuscript.

1. The authors demonstrated that CD31 is enriched in microglia compared with neurons and astrocytes. However, the data shown in Supplementary Fig. S2 suggested that microglia only contributed approximately one-fifth to one-quarter of hippocampal CD31 levels. The authors may clarify this point further by comparing microglial CD31 with endothelial CD31 in the brain.

2. The authors may discuss what is the impact of microglia-specific CD31 knockout on other neural cells.

3. Studies have shown that the phagocytic function of microglia depends on the activation of the TREM2/TYROBP/Syk pathway. TYROBP is a tyrosine kinase-binding protein that can bind to TREM2, leading to the phosphorylation of tyrosine residues in the ITAM domain of TYROBP's intracellular region, recruiting Syk kinase, and activating downstream signaling molecules, one of which is STAT3. TREM2 and CD31 may share overlapping signaling regulatory mechanisms. The authors may discuss the potential relationship between the two.

4. In Fig. 3, the authors concluded that CD31 knockdown accelerated Aβ degradation without affecting initial phagocytosis. While the underlying assays appear appropriate, the text should more clearly state that fluorescence at the initial time point reflects Aβ uptake, whereas the decline in intracellular fluorescence over time reflects degradative processing.

5. Given that intracerebroventricular delivery limits the translational applicability of the P1 peptide, the authors are encouraged to briefly discuss potential strategies for achieving more clinically feasible delivery, such as the use of blood–brain barrier–penetrant modifications or nanocarrier systems.

6. My another suggestion is to have an editorial editing support to correct a few typos . For examples, Line 113: suggest to change "in brain tissue" to "in the brain”. Line 222, such asMRC1, should be such as MRC1,

Reviewer #2

(Remarks to the Author)

The manuscript entitled ‘Microglial CD31 Orchestrates Alzheimer ’s Pathology via the SHP2– STAT3–MME Axis to Restrain Aβ Clearance’ by Zhou & Sun et al. describe the upregulation of CD31 on microglia in AD cases, AD mouse models and microglial models after amyloid exposure. Genetic knock-down of CD31 (C31KD) by heterozygous deletion in 5xFAD mice improved cognition, reduced amyloid beta plaque load and reduced the activation (interferon, DAM-like gene expression, etc) of microglia, while enabling their ability to cluster around plaques. Both their transcriptional data as well as in vitro phagocytosis assays suggested enhanced degradative capacity in CD31KD microglia through the upregulation of MME. The authors further demonstrated through IP-based experiments that CD31 could interact with both SHP2 and STAT3 to modulate pSTAT3(Y702) levels, ultimately modulating MME transcription and protein levels. They identified that pre-treatment of cells or 5xFAD mice with the P1 peptide, mimicking an n-terminal fragment of CD31 shown to interact with STAT3, would interfere with full-length CD31-STAT3 interactions, leading to increased pSTAT3 and MME levels and improvements in cognitive behavior and amyloid burden.
In general, the fact that CD31 can modulate amyloid beta pathology load by regulating its digestion is of interest, especially given the current efforts in unraveling the role of microglia in either clearing or seeding plaques. The effect sizes are large and the authors have identified a potentially druggable pathway that could alter pathology load and cognitive deficits. However, several major and minor points would need to be addressed:

1. The conceptual rationale for investigating CD31 expression on microglia and its role in AD is currently not convincing. Many microglial proteins change expression in AD and AD mouse models, why did you chose this one?
2. In general, the materials & methods section contains far too little details on how experiments were executed and is lacking description of many experiments as it stands. For example (and this list is not inclusive), I am missing methods on the generation of the primary cultures for astrocytes & neurons, the Abeta phagocytosis assay with primary microglia, the generation of oligomeric Abeta, the bulk transcriptomics, the experimental set-up of the treatment of primary microglia with the Stattic compound, the in vivo study for the treatment of mice with the P1 compound (surgery, compound formulation, sex of the mice, etc), the nuclei isolation protocol, many of the scRNAseq analyses (more on this later), details on image-based analyses (number of mice, ROIs/cells, analysis description), etc, etc.
3. Statistics:
a. For transparency, please add a supplemental table detailing all statistical outcomes, including t and F statistics, evidence of meeting the assumptions for parametric testing, etc. It is currently not clear which post-hoc corrections were performed for the pair-wise comparisons following the ANOVA analyses.
b. When using bar graphs, please also display the individual datapoints and not only mean +/- SEM.
c. Fig.2B: instead of performing 9 independent ANOVAs (which inflates your chance of type I errors), the authors should use differential abundance analysis. With DAA you also control for the interdependencies that exist across your different clusters, i.e. when one cluster increases another should go down, given the proportional nature of the data.
d. Fig.3F-H: the authors must account for the potential type I inflation by using so many t-tests and the fact that these different timepoints are not independent observations (fig.3G). I.e. use of a repeated measures ANOVA (3G) or 2x ANOVA (3H) are warranted. Additionally, what do the authors mean with ‘n=3’?
4. Single nuclei seq experiment:
a. Quite some details are missing concerning the single nuc RNAseq experiment. What strain is the ‘ctrl’ group in Fig.2.? Which ‘brain region’ was used for the single nuc extractions and how was this executed (M&M is missing entirely for this)? How many mice & which sex were used per genotype?
b. Which criteria were used for the DE analysis (FDR corrections, p-value stringency, potential log2(fold change) cut-offs)? How many genes were found significantly DE per comparison and could you provide supplemental tables with those genes? How was the pathway enrichment executed (software, background dataset, FDR & enrichment criteria etc)? For Fig.S3F: it would be helpful if terms that do not survive FDR correction would be blanked out. Fig.S4B: what does the color scale represent? Clearly this graph does not contain 362 genes as stated in the legend. How where these genes selected?
c. Trajectory analysis (methods is also missing) is anyway very amenable to the data that you put in: e.g. UMAP constellation and the amount of data that is given to perform the analysis can drastically shape your outcome and therefore interpretation. These analyses should anyway be interpreted with a grain of salt, but particularly when only selecting 3 groups of interest. If the authors wish to include this analysis, it must be re-run on the entire microglia dataset (including all microglial subclusters) and then re-assess whether these transitions still occur as the authors predict now based on this limited cluster set. The current visualization in fig.2D also makes it difficult for the reader to interpret where the transitional difference between 5xFAD and CD31KO*5xFAD lies: is it the transition from HM to DAM or from DAM to IFN?
d. Pages 10/11: there is a lot of emphasis on the intracluster DE analysis between genotypes. However, some of these results could be driven by the discrete nature of cluster-based approaches, pushing specific cells either in one cluster or the other, not considering that microglia are by nature dynamic. Thus instead of homeostatic microglia ‘loosing’ of ‘gaining’ specific characteristics (as evidenced by the DE analysis), it could be that the MG of one genotype simply lean more towards membership of a different cluster (but are still categorized as HM), thereby driving the DE analysis. Although I think overall many of these observations still hold true (i.e. KO of CD31 shapes MG to a less inflammatory state), this paragraph could be condensed.
5. The in vivo study with P1 treatment:
a. Several details are missing on the experimental set-up: a) what is the half-life of P1 and how was the dosing determined; b) how did the mice tolerate treatment (e.g. weight loss, wellbeing scores etc); c) was dosing continued during behavioral testing and how was this timed?
b. Additional demonstration of target engagement in vivo on the level of pSTAT3, CD31/SHP2 interaction and MME levels would warranted as well as demonstration that the reduction in plaque level are truly due to alterations in the microglia-

plaque interaction. Can you demonstrate that in vivo that the reduced levels of amyloid-beta are truly due to enhanced clearance of plaques by microglia and not due to alterations in amyloid production? Are there additional targets that P1 could bind to that could explain any of these results?
c. Conceptually, if downregulating CD31 (or preventing CD3/STAT3 interaction) can reduce plaque load by faster amyloid degradation and improve cognition, could earlier treatment also prevent plaques from forming?
6. The discussion currently feels immature: it does not position the proposed work within the existing literature and it is mainly a summary of the findings. It lacks critical review of the data in the broader context. Some discussion on what could drive upregulation of CD31 during the course of AD pathology, would also be appreciated.

Minor:
1. The references in the introduction are off. I cannot figure out which reference belongs to which part in the text.
2. The authors should provide supplementary tables with the microglia cluster annotations as well as the DE analyses of the different mouse comparison that underlie Fig.S3F and S4.
3. Somehow TREM2 is always spelled TREMG2, also in references.
4. Fig.1: please add details on the axis of most figures or define in Fig. legend. E.g. Fig.1a: are these normalized counts, expression values, RKPM? Fig.1F: what is the surface measure?
5. The color legend is missing for Fig.S5C-E, thus I cannot interpret which violin belongs to which genotype.
6. Fig. 3D/E: given the difficulty with resolving individual cell bodies in plaque surrounding microglia: how did the authors manage to count those?
7. Fig.4B-C: what is meant with ‘gene ratio’ on the x-axis?
8. P.14, lines 60-61: ‘MME was significantly elevated (Fig.4D)’ unless you provide p-values the reader cannot judge whether the elevation is indeed significant.
9. Fig.4F: can you indicate which of these gene changes survive FDR correction by e.g. using asterisks?
10. Can you disclose where the anti-CD31 antibody binds (which amino acids)? Just to understand whether pre-incubation with the P1 fragment, does not lead to detection of P1, instead of full length CD31 when assessing the CD31 load in Fig. S6A.
11. P19, line 522: STAT1 or STAT3?
12. Please detail the exact mouse strain (catalog number) that was used from Cyagen Biosciences.

Reviewer #3

(Remarks to the Author)
This study by Zhou et al. investigates the role of microglial CD31 in Alzheimer's disease pathogenesis. CD31 has long been recognized as an endothelial marker, whereas its expression and function in microglia are largely unknown. By analyzing published human and mouse single cell RNAseq datasets, the authors uncovered CD31 expression in microglia but not in neurons and astrocytes. Intriguingly, its expression was upregulated in the microglia of AD brains. To investigate its function in microglia in the setting of amyloid deposition, the author generated microglia-specific CD31 haploinsufficiency 5xFD mice. Through single-nucleus RNA sequencing, immunohistological analysis, and behavioral studies, the author demonstrated that deleting one allele of CD31 in microglial improves cognitive function, reduced Abeta load, confer microglia into homeostatic state. Mechanistically, the author demonstrated CD31 functions through STAT3/MME pathway. Overall, the paper is novel, and the results support the conclusion that microglial CD31 promotes AD pathology by suppressing STAT3 activation and reducing Aβ clearance.

Major Concerns:
1. The paper only included Cx3cr1Cre;CD31fl/+ heterozygous knockdown. The author should at least discuss the reason why they did not include homozygous knockout in addition to single allele deletion.
2. CD31 Knockdown reduces microglial numbers in 5xFAD mice. The authors interpreted this finding as reduced microglial activation. This is inaccurate. Microglial activation refers the cell state not cell number. On the other hand, it would be good to evaluate whether the reduced cell number is due to decreased proliferation or increased apoptosis.
3. It would be important to clarify whether P1 influences endothelial CD31 function, given that 81% of brain CD31 is in non-microglial cells, largely endothelial cells.
4. The rationale for using 1 mM P1 in the in vivo experiments is unclear. Did the authors perform any dose–response analysis?
5. Although the authors demonstrate physical interactions among CD31, STAT3, and SHP2 using co-IP and PLA, the functional role of SHP2 in this pathway remains untested. The study does not assess SHP2 phosphatase activity, test whether SHP2 inhibition or knockdown recapitulates the consequences of CD31 deficiency or determine whether the P1 peptide disrupts SHP2 recruitment in addition to CD31–STAT3 binding. These experiments will make the involvement of SHP2 in mediating CD31-dependent suppression of STAT3 phosphorylation largely correlative, so additional functional validation is advised to substantiate the proposed CD31–SHP2–STAT3 signaling mechanism.

Minor comments:
Page 9 line 213: homeostatic cluster should be MG0, not MG1, since MG1 is DAM cluster.
Fig S5 C-E: It is not clear what the red, green, and blue colored violin plots are representing.

Version 1:

Reviewer comments:

Reviewer #1

(Remarks to the Author)
Authors of this study have been quite responsive to previous critics and have addressed each point in a rigorous manner. I have no additional concern to this well revised manuscript. With these extensive revised text and additional results to support their conclusion, this manuscript has improved significantly. IMO, this study is timely and highly important to the field. There is no doubt that authors have identified a novel SHP2–STAT3–MME Axis, regulated by CD31, in the control of clearance of Ab aggregates. This exciting finding paves a new path for future therapeutic intervention for AD treatment.

Reviewer #2

(Remarks to the Author)
The authors have addressed all the comments I had in a satisfactory manner. If possible I would advice to reduce the discussion section somewhat to keep it more succinct & reader-friendly.

Reviewer #3

(Remarks to the Author)
The authors have done an excellent job addressing my concerns. The revised manuscript is compelling and will be of strong interest to both readers and the field.

Open Access This Peer Review File is licensed under a Creative Commons Attribution 4.0 International License, which permits use, sharing, adaptation, distribution and reproduction in any medium or format, as long as you give appropriate credit to the original author(s) and the source, provide a link to the Creative Commons license, and indicate if changes were made.
In cases where reviewers are anonymous, credit should be given to 'Anonymous Referee' and the source.
The images or other third party material in this Peer Review File are included in the article's Creative Commons license, unless indicated otherwise in a credit line to the material. If material is not included in the article's Creative Commons license and your intended use is not permitted by statutory regulation or exceeds the permitted use, you will need to obtain permission directly from the copyright holder.
To view a copy of this license, visit https://creativecommons.org/licenses/by/4.0/

Reviewer #1:

The manuscript by Zhou et al investigated the role of microglia CD31 in AD and identified the SHP2–STA T3–MME axis

For regulating AD pathogenesis. In this study, authors took the advantage of available single sequencing data and showed the elevated expression of CD31 in AD microglia. Authors then generated CD31f/fl mouse line and crossed with Cx3cr1-Cre driver mice to delete CD31 either in the WT or 5xFAD mouse background for comparative studies, which include experiments to examine the cognitive behaviors such open filed, fear conditioning, novel objective and Morris later maze, amyloid pathological changes, Abeta levels, single nuclear Seq profiling, and further mechanistic explorations. The entire manuscript has a very comprehensive set of experimental results that are presented in a step by step and logically developed manner. In my evaluation, results are supportive to their conclusion that targeted deletion of CD31, even partially, is sufficient to decrease amyloid pathology through mitigating microglia activation and restoring more homeostatic microglia. The experimental design is rigor. RNAseq data analysis is highly informative for helping with the mechanistic studies. The results for supporting the STAT3-MME pathway are striking and convincing. The most exciting finding is the identification of P1 Peptide for blocking CD31 to phosphorylate STAT3 and reduce Amyloid plaques in 5xFAD mice. The manuscript is mostly written in a logical style, and the conclusion is novel and important for the field, especially considering its potential translational value. IMO, this study should be published with no long delay. To strengthen this study further, I have a few suggestions to authors in this manuscript.

1. The authors demonstrated that CD31 is enriched in microglia compared with neurons and astrocytes. However, the data shown in Supplementary Fig. S2 suggested that microglia only contributed approximately one-fifth to one-quarter of hippocampal CD31 levels. The authors may clarify this point further by comparing microglial CD31 with endothelial CD31 in the brain.

Response: We thank the reviewer for this suggestion to further clarify the relative contributions of microglia and endothelial cells to CD31 expression in the brain.

As shown in Supplementary Fig.2, microglia-specific deletion of CD31 leads to an approximately 20–25% reduction of total CD31 protein levels in the hippocampus, indicating that endothelial cells remain the major quantitative source of CD31 in brain tissue. We agree that this point should be more explicitly stated to avoid potential ambiguity.

Importantly, our study focuses on the cell-type–specific and disease-responsive role of CD31 in AD rather than its bulk abundance. While endothelial CD31 expression is relatively stable, microglial CD31 is selectively enriched among non-endothelial neural cells and is markedly upregulated in AD. Despite contributing a smaller fraction of total CD31 protein, microglial CD31 exerts a disproportionate functional impact on AD pathology, as partial and microglia-

specific CD31 reduction is sufficient to ameliorate microglial activation, enhance Aβ degradation, and improve cognitive function.

We have clarified this distinction between quantitative contribution and functional relevance in the revised manuscript (please see page 22, lines 578–582 and lines 596-603 in the revised discussion).

2. The authors may discuss what is the impact of microglia-specific CD31 knockout on other neural cells.

Response: We thank the reviewer for this suggestion. To address this point, we performed additional experiments to examine the impact of microglia-specific CD31 knockdown on neurons. Specifically, we examined synaptic integrity in hippocampal tissues by assessing the expression of synapse-related proteins using Western blot analysis and by performing Golgi staining to evaluate dendritic spine density. We found that microglial CD31 knockdown significantly increased the levels of postsynaptic proteins (PSD95, GluN2A, and GluN2B, revised Fig1T, U) and markedly elevated dendritic spine density in 5xFAD mice (revised Fig1V), indicating improved synaptic structure. These results have been incorporated into the revised manuscript in the Results section (please see page 7-8, lines 167-172). In addition, we have added a brief discussion to clarify the potential impact of microglial CD31 deficiency on neurons (please see page 25, lines 672-680 in revised manuscript).

3. Studies have shown that the phagocytic function of microglia depends on the activation of the TREM2/TYROBP/Syk pathway. TYROBP is a tyrosine kinase-binding protein that can bind to TREM2, leading to the phosphorylation of tyrosine residues in the ITAM domain of TYROBP's intracellular region, recruiting Syk kinase, and activating downstream signaling molecules, one of which is STAT3. TREM2 and CD31 may share overlapping signaling regulatory mechanisms. The authors may discuss the potential relationship between the two.

Response: We thank the reviewer for this comment. In the revised manuscript, we have incorporated an expanded discussion clarifying the relationship between TREM2/TYROBP/Syk signaling and the CD31–STAT3–MME axis. Specifically, we discuss how CD31 may function within the broader TREM2-driven signaling network at the level of downstream transcriptional regulation. Please see the revised Discussion section (page 23, lines 617-620; page 26-27, lines 716-734) for details.

4. In Fig. 3, the authors concluded that CD31 knockdown accelerated Aβ degradation without affecting initial phagocytosis. While the underlying assays appear appropriate, the text should more clearly state that fluorescence at the initial time point reflects Aβ uptake, whereas the decline in intracellular fluorescence over time reflects degradative processing.

Response: We thank the reviewer for this suggestion. We have revised the text to more clearly

state that the fluorescence signal at the initial time point reflects Aβ uptake, whereas the subsequent decline in intracellular fluorescence over time reflects degradative processing of internalized Aβ. Please see page 13-14, lines 342–346 and lines 349-353 in the revised manuscript.

5. Given that intracerebroventricular delivery limits the translational applicability of the P1 peptide, the authors are encouraged to briefly discuss potential strategies for achieving more clinically feasible delivery, such as the use of blood–brain barrier–penetrant modifications or nanocarrier systems.

Response: We thank the reviewer for this suggestion regarding the translational applicability of the P1 peptide. We agree that intracerebroventricular delivery, while useful for proof-of-concept studies, is not ideal for clinical translation.

In the revised manuscript, we have added a brief discussion addressing potential strategies to improve the clinical feasibility of P1 delivery. These include chemical modifications to enhance blood–brain barrier penetration, such as conjugation with cell-penetrating peptides or lipid-based moieties, as well as the use of nanocarrier-based delivery systems (e.g., liposomes, polymeric nanoparticles, or exosome-based vehicles) to facilitate systemic administration and brain targeting. We emphasize that these approaches represent important future directions for optimizing P1 delivery and translation, rather than components of the current study. Please see page 28, lines 755–763 in the revised discussion.

6. My another suggestion is to have an editorial editing support to correct a few typos . For examples, Line 113: suggest to change "in brain tissue" to "in the brain”. Line 222, such asMRC1, should be such as MRC1,

Response: We thank the reviewer for this suggestion. We have carefully revised the manuscript to correct the typographical and formatting errors highlighted by the reviewer, including the changes suggested at lines 113 and 222. Please see the revised manuscript at lines 133 and 252-253. In addition, the entire manuscript has been thoroughly checked to ensure consistency and clarity in language. The relevant corrections have been incorporated into the revised version.

Reviewer #2 (Remarks to the Author):

The manuscript entitled ‘Microglial CD31 Orchestrates Alzheimer ’s Pathology via the SHP2–STAT3–MME Axis to Restrain Aβ Clearance’ by Zhou & Sun et al. describe the upregulation of CD31 on microglia in AD cases, AD mouse models and microglial models after amyloid exposure. Genetic knock-down of CD31 (C31KD) by heterozygous deletion in 5xFAD mice improved cognition, reduced amyloid beta plaque load and reduced the activation (interferon, DAM-like gene expression, etc) of microglia, while enabling their ability to cluster around plaques. Both their transcriptional data as well as in vitro phagocytosis assays suggested

enhanced degradative capacity in CD31KD microglia through the upregulation of MME. The authors further demonstrated through IP-based experiments that CD31 could interact with both SHP2 and STAT3 to modulate pSTAT3(Y702) levels, ultimately modulating MME transcription and protein levels. They identified that pre-treatment of cells or 5xFAD mice with the P1 peptide, mimicking an n-terminal fragment of CD31 shown to interact with STAT3, would interfere with full-length CD31-STAT3 interactions, leading to increased pSTAT3 and MME levels and improvements in cognitive behavior and amyloid burden.

In general, the fact that CD31 can modulate amyloid beta pathology load by regulating its digestion is of interest, especially given the current efforts in unraveling the role of microglia in either clearing or seeding plaques. The effect sizes are large and the authors have identified a potentially druggable pathway that could alter pathology load and cognitive deficits. However, several major and minor points would need to be addressed:

1. The conceptual rationale for investigating CD31 expression on microglia and its role in AD is currently not convincing. Many microglial proteins change expression in AD and AD mouse models, why did you chose this one?

Response: We thank the reviewer for raising this question regarding the conceptual rationale for focusing on CD31. In response to this concern, we have substantially revised and expanded the Introduction to more clearly articulate the relationship between CD31 and AD, thereby strengthening the scientific motivation of the present study.

Specifically, in the revised Introduction, we summarize multiple independent clinical studies showing that CD31 protein levels are significantly elevated in the plasma and cerebellar tissues of AD patients compared with age-matched controls. We further incorporate evidence from several transcriptomic analyses demonstrating that CD31 mRNA expression is markedly increased across multiple brain regions in AD patients. In addition, we include experimental evidence from animal models showing that injection of Aβ_{1-42} into the hippocampal CA1 region of mice leads to a significant increase in CD31 expression in brain tissue, supporting a direct link between Aβ-related pathological stimulation and CD31 upregulation. These revisions and the corresponding references are now presented in the revised Introduction (Please see page 4, lines 80-89).

Building on this background, we further emphasize that although CD31 has long been regarded as a vascular-associated molecule, emerging single-cell and transcriptomic studies consistently indicate substantial CD31 expression in microglia, with disease-associated upregulation in microglia from AD patients. Consistently, transcriptomic analyses of human iPSC-derived microglia treated with oligomeric Aβ also reveal a significant increase in CD31 transcripts. Importantly, we explicitly acknowledge in the revised Introduction that these observations are

largely derived from large-scale transcriptomic and single-cell analyses, and that experimental validation of CD31 expression and function in microglia has been lacking. (Please see page 4, lines 90-101 in the revised Introduction).

Through these revisions, we aim to clarify that CD31 was selected based on converging evidence from human clinical samples, multiple independent transcriptomic datasets, and AD-relevant experimental models, rather than as one of many nonspecifically altered microglial markers. At the same time, we highlight a critical knowledge gap regarding the functional and mechanistic role of microglial CD31 in AD. This gap directly motivated the present study, in which we experimentally validate CD31 expression in microglia and systematically investigate its functional significance and molecular mechanisms in AD pathology.

2. In general, the materials & methods section contains far too little details on how experiments were executed and is lacking description of many experiments as it stands. For example (and this list is not inclusive), I am missing methods on the generation of the primary cultures for astrocytes & neurons, the Abeta phagocytosis assay with primary microglia, the generation of oligomeric Abeta, the bulk transcriptomics, the experimental set-up of the treatment of primary microglia with the Stattic compound, the in vivo study for the treatment of mice with the P1 compound (surgery, compound formulation, sex of the mice, etc), the nuclei isolation protocol, many of the scRNAseq analyses (more on this later), details on image-based analyses (number of mice, ROIs/cells, analysis description), etc, etc.

Response: We thank the reviewer for this comment. We agree that the initial version of the Methods section lacked sufficient detail.

In response, we have thoroughly revised and substantially expanded the Methods section to provide comprehensive descriptions of all experimental procedures, including primary cell culture generation, Aβ-related assays, transcriptomic analyses, pharmacological treatments, in vivo P1 administration, nuclei isolation, single-cell/nucleus RNA-seq analyses, and image-based quantifications. Relevant experimental parameters, sample sizes, and analysis procedures are now clearly described in the Methods section of the revised manuscript.

3. Statistics:

a. For transparency, please add a supplemental table detailing all statistical outcomes, including t and F statistics, evidence of meeting the assumptions for parametric testing, etc. It is currently not clear which post-hoc corrections were performed for the pair-wise comparisons following the ANOVA analyses.

Response: We thank the reviewer for this suggestion. To improve transparency and reproducibility, we have now added a new supplementary table (please see revised Supplementary Table 2) that summarizes all statistical analyses performed in the study. This

table includes the type of statistical test used, exact t and F statistics, degrees of freedom, and corresponding P values for each analysis. In addition, we have clarified in the revised Statistical Analysis section that the assumptions for parametric testing (normality and homogeneity of variance) were evaluated prior to analysis, and the corresponding results are provided in the supplementary statistics table (please see page 38, lines 1072-1080 and revised Supplementary Table 2). We have also clearly indicated the post-hoc procedures used following ANOVA analyses, which are now specified in the relevant figure legends to ensure clarity regarding the statistical tests applied in each experiment.

b. When using bar graphs, please also display the individual datapoints and not only mean +/- SEM.

Response: We thank the reviewer for this suggestion. In the revised manuscript, all bar graphs have been updated to display the individual data points in addition to the mean ± SEM. Please see the revised main figures and supplementary figures for these modifications.

c. Fig.2B: instead of performing 9 independent ANOVAs (which inflates your chance of type I errors), the authors should use differential abundance analysis. With DAA you also control for the interdependencies that exist across your different clusters, i.e. when one cluster increases another should go down, given the proportional nature of the data.

Response: We thank the reviewer for this statistical suggestion. We agree that performing multiple independent ANOVAs may inflate the risk of type I errors and does not adequately account for the compositional nature and interdependencies among cell clusters. In the revised manuscript, we have therefore replaced the multiple ANOVA-based comparisons in Fig. 2B with a differential abundance analysis (DAA). The updated results and the revised figure are now presented in the revised manuscript (please see revised Fig. 2B), and the corresponding statistical methodology has been updated accordingly in the figure legend.

d. Fig.3F-H: the authors must account for the potential type I inflation by using so many t-tests and the fact that these different timepoints are not independent observations (fig.3G). I.e. use of a repeated measures ANOVA (3G) or 2x ANOVA (3H) are warranted. Additionally, what do the authors mean with 'n=3'?

Response: We thank the reviewer for raising these statistical concerns. We agree that performing multiple t-tests across time points may increase the risk of type I errors and that repeated measurements from the same experimental units would require appropriate repeated-measures analysis.

In the revised manuscript, we have therefore reanalyzed the data using two-way ANOVA. Importantly, the measurements at different time points in Fig. 3G were not obtained from the same cells over time. In this experiment, primary microglia from WT and CD31 knockdown

mice were first incubated with fluorophore-labeled Aβ$_{1-42}$ oligomers for 2 hours to allow uptake, after which extracellular Aβ was removed by PBS washing (defined as time 0). The degradation of internalized Aβ was then monitored over time. For each time point (0 h, 3 h, 6 h, and 9 h), separate wells of cells were fixed and analyzed, rather than repeatedly imaging the same cells over time. Therefore, the observations at different time points represent independent measurements derived from different wells, and are not repeated measurements of the same experimental units. Based on this experimental design, repeated-measures ANOVA is not appropriate, and a two-way ANOVA was applied to assess the effects of genotype and time and their interaction.

Similarly, the ELISA-based quantification of intracellular Aβ levels shown in Fig. 3H was also analyzed using two-way ANOVA to evaluate the effects of genotype and time, followed by appropriate post-hoc multiple comparison tests as described in the revised Statistical Analysis section and legends.

Regarding the notation "n = 3," we have clarified in the revised figure legends that n refers to three independent experiments. This clarification has been added to avoid ambiguity.

4. Single nuclei seq experiment:

a. Quite some details are missing concerning the single nuc RNAseq experiment. What strain is the 'ctrl' group in Fig.2.? Which 'brain region' was used for the single nuc extractions and how was this executed (M&M is missing entirely for this)? How many mice & which sex were used per genotype?

Response: We thank the reviewer for pointing out these missing details. We have now clarified the experimental design and added the relevant information to the revised manuscript. Specifically, the "ctrl" group shown in Fig. 2 refers to littermate control wild type mice. For the single-nucleus RNA-seq experiments, nuclei were isolated from whole brain tissue excluding the cerebellum. Each genotype included three mice, and each group consisted of two male and one female mice for snRNA-seq analysis. Importantly, the detailed nuclei isolation procedure has now been added to the Methods section (please see page 36-37, lines 1007-1027).

b. Which criteria were used for the DE analysis (FDR corrections, p-value stringency, potential log2(fold change) cut-offs)? How many genes were found significantly DE per comparison and could you provide supplemental tables with those genes? How was the pathway enrichment executed (software, background dataset, FDR & enrichment criteria etc)? For Fig.S3F: it would be helpful if terms that do not survive FDR correction would be blanked out. Fig.S4B: what does the color scale represent? Clearly this graph does not contain 362 genes as stated in the legend. How where these genes selected?

Response: We thank the reviewer for these detailed and constructive comments. We apologize

for the lack of clarity in the original manuscript and appreciate the opportunity to provide a more explicit and transparent description of the differential expression (DE) analyses, pathway enrichment strategies, and figure-specific implementations. We address each point raised by the reviewer below.

1) Which criteria were used for the DE analysis (FDR corrections, p-value stringency, potential log2(fold change) cut-offs)?

Different DE criteria were applied depending on the analytical purpose of each figure, and these are now clarified explicitly.

For Fig. 2C and Fig. 2D (previously Fig.2E, F), which focus on pathway enrichment analyses of MG0, MG1, and MG5 clusters, DE genes were defined using nominal statistical significance. Genes with a raw P value < 0.05 and abs(average log2(fold change))> 0.25 were considered differentially expressed and used as input for pathway enrichment. For cluster MG5, no genes reached FDR significance (adjusted p-value < 0.05), likely due to limited statistical power caused by small cell numbers and weak effect sizes. Therefore, we reported genes with nominal significance (unadjusted p-value < 0.05) as candidate markers for exploratory purposes, and interpreted them cautiously

For Supplementary Fig. 3F and Supplementary Fig. 5A (previously Fig.S3F and Fig.S4C), DE genes were defined as genes with an adjusted P value < 0.05 after FDR correction, together with abs(average log2(fold change))> 0.25. These criteria were used to ensure robust identification of DE genes in downstream comparative and enrichment analyses.

For Supplementary Fig.5B (previously Fig.S4B), DE genes were identified using a pseudo-bulk differential expression strategy in Micro-PVM cells. Genes were first tested for differential expression in each comparison at the pseudo-bulk level, and those with a nominal P value < 0.05 were considered significant. “Reversed genes” were then defined as genes that were significantly differentially expressed (p < 0.05, pseudo-bulk) in the comparison between 5xFAD-WT and Ctrl mice and also significantly differentially expressed (p < 0.05, pseudo-bulk) with an opposite direction of change in the comparison between Cx3cr1^{Cre}CD31$^{fl/+}$5xFAD and 5xFAD-WT mice.

2) How many genes were found significantly DE per comparison and could you provide supplemental tables with those genes?

The numbers of significantly DE genes identified in each comparison are summarized below.

For Fig. 2C–D (previously Fig.2E, F), comparison of Cx3cr1^{cre}CD31$^{fl/+}$5xFAD vs 5xFAD-WT identified 1,113 DE genes in MG0 (908 downregulated, 205 upregulated), 1,080 DE genes in MG1 (902 downregulated, 178 upregulated), and 372 DE genes in MG5 (277 downregulated, 95 upregulated).

For Supplementary Fig. 3F (previously Fig.S3F), comparison1 (5xFAD-WT vs Ctrl) identified 1159 DE genes (569 downregulated, 590 upregulated), comparison2 (Cx3cr1^{cre}CD31$^{fl/+}$5xFAD vs 5xFAD-WT) identified 2293 DE genes (1183 downregulated, 1110 upregulated), and comparison3 (Cx3cr1^{cre}CD31$^{fl/+}$5xFAD vs Ctrl) identified 1330 DE genes (538 downregulated, 792 upregulated).

For Supplementary Fig. 5A (previously Fig.S4A), comparison1 (5xFAD-WT vs Ctrl) identified 599 DE genes (147 downregulated, 452 upregulated) and comparison2 (Cx3cr1^{cre}CD31$^{fl/+}$5xFAD vs 5xFAD-WT) identified 904 DE genes (794 downregulated, 110 upregulated).

For Supplementary Fig. 5B (previously Fig.S4B), a total of 59 significantly differentially expressed "reversed genes" were identified based on pseudo-bulk analysis ($p < 0.05$ in both comparisons with opposite directions of change).

All DE gene lists are provided in the newly added supplementary tables, please see revised Supplementary Table 3.

3） How was the pathway enrichment executed (software, background dataset, FDR & enrichment criteria etc)?

Pathway enrichment analyses were performed using the R package clusterProfiler. Differentially expressed genes identified from the single-nucleus RNA-seq data were used as input gene sets, and all genes detected in the corresponding dataset were used as the background gene set. GO biological process terms and KEGG-based pathway annotations were used for functional interpretation.

For Fig. 2C and Fig. 2D (previously Fig.2E, F), pathway enrichment was performed using DE genes defined by nominal significance thresholds, and enrichment significance was assessed based on adjusted P values.

For pathway enrichment analyses shown in Supplementary Fig. 3F and Supplementary Fig. 5B (previously Fig.S3F and Fig.S4B), enrichment significance was assessed using Benjamini–Hochberg false discovery rate (FDR) correction, and pathways with an adjusted P value < 0.05 were considered significantly enriched.

These criteria were explicitly specified for each figure in the revised Methods section and corresponding figure legends to ensure transparency and clarity.

4）For Fig.S3F: it would be helpful if terms that do not survive FDR correction would be blanked out.

For Supplementary Fig.3F (previously Fig.S3F), we have remade the heatmap following reviewer's kind suggestions which blanked out terms that do not survive FDR correction.

5) Fig.S4B: what does the color scale represent? Clearly this graph does not contain 362 genes as stated in the legend. How where these genes selected?

For Supplementary Fig.5B (previously Fig.S4B), the heatmap displays $\log_2$ fold changes derived from pseudo-bulk differential expression analysis for reversed genes that were significantly differentially expressed ($p < 0.05$, pseudo-bulk) in Micro-PVM cells. The color scale indicates the magnitude and direction of log2 fold change across comparisons. Reversed genes were defined as genes that were significantly differentially expressed ($p < 0.05$) in the comparison between 5xFAD-WT and control mice and showed significant differential expression with an opposite direction of change ($p < 0.05$) in the comparison between $Cx3cr1^{Cre}CD31^{fl/+}$5xFAD and 5xFAD-WT mice, based on pseudo-bulk analysis. A total of 59 reversed genes met these pseudo-bulk criteria and are shown in Supplementary Fig. 5B. The previously mentioned number (362 genes) corresponds to the DE gene set identified at the single-cell level in Supplementary Fig. 5A, whereas Supplementary Fig. 5B is based on pseudo-bulk analysis, which applies a more stringent sample-level comparison and therefore yields a smaller set of significant genes. This clarification has been incorporated into the revised figure legend.

c. Trajectory analysis (methods is also missing) is anyway very amenable to the data that you put in: e.g. UMAP constellation and the amount of data that is given to perform the analysis can drastically shape your outcome and therefore interpretation. These analyses should anyway be interpreted with a grain of salt, but particularly when only selecting 3 groups of interest. If the authors wish to include this analysis, it must be re-run on the entire microglia dataset (including all microglial subclusters) and then re-assess whether these transitions still occur as the authors predict now based on this limited cluster set. The current visualization in fig.2D also makes it difficult for the reader to interpret where the transitional difference between 5xFAD and CD31KO*5xFAD lies: is it the transition from HM to DAM or from DAM to IFN?

Response: We thank the reviewer for this comment. We fully agree that trajectory inference and pseudotime analyses are highly sensitive to data selection, embedding structure (e.g., UMAP configuration), and the subset of cells included, and that results derived from a restricted set of microglial clusters should therefore be interpreted with caution.

In light of the reviewer's concerns, we have decided to remove the trajectory (pseudotime) analysis from the revised manuscript. Specifically, we have deleted both the trajectory visualization shown in Fig. 2C and the associated pseudotime quantification in Fig. 2D, as well as all corresponding descriptions in the Results and Methods sections. By doing so, we avoid potential over-interpretation of microglial state transitions inferred from analyses based on a limited subset of clusters.

As a result, the revised manuscript no longer relies on trajectory-based inferences (e.g., HM–

DAM–IFN transitions) to support our conclusions. Instead, we focus on more robust and directly supported analyses of microglial states and transcriptional differences across genotypes. We believe that this revision substantially improves the rigor and clarity of the study.

d. Pages 10/11: there is a lot of emphasis on the intracluster DE analysis between genotypes. However, some of these results could be driven by the discrete nature of cluster-based approaches, pushing specific cells either in one cluster or the other, not considering that microglia are by nature dynamic. Thus instead of homeostatic microglia 'loosing' of 'gaining' specific characteristics (as evidenced by the DE analysis), it could be that the MG of one genotype simply lean more towards membership of a different cluster (but are still categorized as HM), thereby driving the DE analysis. Although I think overall many of these observations still hold true (i.e. KO of CD31 shapes MG to a less inflammatory state), this paragraph could be condensed.

Response: We thank the reviewer for this comment and agree that intracluster differential expression analyses can be influenced by the discrete nature of clustering approaches, particularly given the dynamic and continuous states of microglia. As the reviewer correctly points out, some observed intracluster differences may reflect a shift in cell state toward neighboring clusters rather than a strict gain or loss of specific homeostatic features within a fixed cluster identity.

In response to this concern, we have revised and condensed the corresponding section on Pages 10–11 to adopt a more cautious interpretation. Specifically, we have reduced emphasis on categorical "loss" or "gain" of microglial characteristics and clarified that these transcriptional changes likely reflect a continuum of microglial states shaped by genotype. Importantly, while condensing this paragraph, we retained the central observation—supported by multiple analyses—that CD31 knockout biases microglia toward a less inflammatory transcriptional profile.We believe that this revision improves conceptual clarity and avoids over-interpretation while preserving the main biological conclusions of the study. (Please see page11-12, lines 271-311)

5. The in vivo study with P1 treatment:

a. Several details are missing on the experimental set-up: a) what is the half-life of P1 and how was the dosing determined; b) how did the mice tolerate treatment (e.g. weight loss, wellbeing scores etc); c) was dosing continued during behavioral testing and how was this timed?

Response: We thank the reviewer for these comments and for highlighting the need to further clarify the experimental design, dosing rationale, and animal welfare assessments.

a) what is the half-life of P1 and how was the dosing determined

We appreciate the reviewer's interest in the pharmacokinetic properties of P1. The current study

was primarily designed to evaluate the biological efficacy and mechanistic effects of P1 rather than to perform a comprehensive pharmacokinetic characterization; therefore, in vivo half-life measurements were not included. We fully agree that detailed pharmacokinetic profiling is essential for the further translational development of P1, and we plan to conduct systematic in vivo pharmacokinetic analyses in future studies, including assessment of half-life and brain distribution.

The in vivo dosing regimen was estimated based on the effective concentration identified in vitro (100 μM) and the reported cerebrospinal fluid (CSF) volume in adult mice, which is typically estimated to be approximately 35–45 μL. Accordingly, administration of 5 μL of a 1 mM P1 solution was expected to yield an initial CSF concentration on the order of ~100 μM after dilution. This strategy was chosen to achieve a biologically relevant exposure comparable to the effective concentration observed in cell-based assays, while considering CSF dilution, turnover, and potential peptide degradation in vivo.

b) how did the mice tolerate treatment (e.g. weight loss, wellbeing scores etc)

We agree and have now clarified animal tolerability and welfare monitoring. Mice were maintained in an SPF facility under standard husbandry conditions with ad libitum access to food and water and were monitored throughout the treatment period. Body weight was recorded weekly, and general wellbeing was assessed by routine clinical observations (including grooming/coat condition, posture, spontaneous activity, respiration, and food/water intake). No abnormal clinical signs or treatment-related mortality were observed, and body weight remained stable without apparent weight loss in P1-treated mice. In addition, histopathological examination (H&E staining) of major organs collected at sacrifice did not reveal overt tissue abnormalities. The corresponding body-weight and H&E data are provided in the revised manuscript (please see revised Supplementary Fig.9, page 19, lines 500-503 and page 20, lines 517-522).

c) was dosing continued during behavioral testing and how was this timed?

P1 administration was not continued during the behavioral testing period. Drug treatment was completed prior to the initiation of behavioral assays, and all behavioral tests were conducted after a defined interval. The overall experimental timeline, including the relationship between drug administration and behavioral testing, is schematically illustrated in Fig. 7A and has now been clearly described in the revised manuscript (please see page 19, lines 497-500).

b. Additional demonstration of target engagement in vivo on the level of pSTAT3, CD31/SHP2 interaction and MME levels would warranted as well as demonstration that the reduction in plaque level are truly due to alterations in the microglia-plaque interaction. Can you demonstrate that in vivo that the reduced levels of amyloid-beta are truly due to enhanced clearance of plaques by microglia and not due to alterations in amyloid production? Are there

additional targets that P1 could bind to that could explain any of these results?

Response: We appreciate the reviewer's detailed comments regarding in vivo target engagement, the mechanism underlying amyloid reduction, and the potential specificity of P1.

To address in vivo target engagement, we performed additional biochemical and histological analyses in brain tissue following P1 treatment. We found that P1 administration significantly increased STAT3 phosphorylation in vivo (please see revised Fig.7L, M), while concomitantly reducing the interaction between STAT3 and CD31(please see revised Fig.7N, O). In contrast, the interaction between CD31 and SHP2 was not altered by P1 treatment (please see revised Fig.7N, O). These results are consistent with our proposed mechanism that P1 selectively disrupts the CD31–STAT3 signaling axis downstream of CD31 without interfering with CD31–SHP2 association. Importantly, P1 treatment also led to a marked increase in MME protein levels, further supporting functional engagement of the CD31–STAT3–MME pathway in vivo (please see page 20, lines 529-538 in revised manuscript).

At the cellular level, immunofluorescence analyses revealed that microglia in P1-treated mice exhibited a morphology more consistent with a homeostatic or resting state (please see revised Fig.7P, Q). Moreover, we observed increased microglial association with amyloid plaques following P1 treatment, indicating enhanced microglia–plaque interaction (please see revised Fig.7R, S). These findings support the conclusion that the reduction in amyloid burden is linked to altered microglial behavior and enhanced plaque clearance (please see page 20, lines 538-542 in revised manuscript).

To determine whether reduced amyloid-beta levels could be attributed to changes in amyloid production, we examined key components of the amyloidogenic pathway. Western blot analyses showed that P1 treatment did not significantly alter the expression levels of APP or the major amyloid-generating enzymes PS1 and BACE1 (please see revised Supplementary Fig. 10, page 20, lines 533-535 in revised manuscript). These results argue against an effect of P1 on amyloid production and instead support a mechanism in which P1 promotes amyloid-beta degradation, at least in part through upregulation of the degrading enzyme MME.

Finally, regarding potential off-target effects, P1 was designed to modulate CD31-associated signaling, and the molecular and cellular changes observed upon P1 treatment are highly consistent with perturbation of the CD31–STAT3–MME axis. While we cannot formally exclude the possibility that P1 may interact with additional targets in vivo, our data do not provide evidence for major off-target effects.

c. Conceptually, if downregulating CD31 (or preventing CD3/STAT3 interaction) can reduce plaque load by faster amyloid degradation and improve cognition, could earlier treatment also prevent plaques from forming?

Response: We agree with the reviewer that this is an important question. Whether earlier intervention could prevent or delay plaque formation is of significant interest. The present study was designed to evaluate therapeutic effects after pathology had already developed and did not address preventive treatment paradigms. We will prioritize investigating the impact of early intervention on plaque formation and disease progression in future studies.

6. The discussion currently feels immature: it does not position the proposed work within the existing literature and it is mainly a summary of the findings. It lacks critical review of the data in the broader context. Some discussion on what could drive upregulation of CD31 during the course of AD pathology, would also be appreciated.

Response: We appreciate the reviewer's comments regarding the Discussion section. We agree that the original version placed too much emphasis on summarizing the results and did not sufficiently engage with the broader literature. In the revised manuscript, we have substantially revised and expanded the Discussion to better position our findings within the context of existing studies and to provide a more critical interpretation of the data. In addition, we have added a dedicated discussion addressing potential mechanisms that may contribute to the upregulation of CD31 during the course of AD pathology. These revisions collectively improve the maturity, balance, and contextualization of the Discussion (please see revised Discussion, page 22-23, lines 596-627; page 26-27, 716-734 and page 27, lines 742-754).

Minor:

1. The references in the introduction are off. I cannot figure out which reference belongs to which part in the text.

Response: We appreciate the reviewer for pointing out this issue. We have carefully reviewed and corrected the references in the Introduction to ensure that each citation now clearly corresponds to the relevant statement in the text. Specifically, we have adjusted the placement and ordering of citations, removed ambiguous or misplaced references, and verified that all cited references are accurately linked to the appropriate sections. The Introduction has been thoroughly revised accordingly to improve clarity and readability.

2. The authors should provide supplementary tables with the microglia cluster annotations as well as the DE analyses of the different mouse comparison that underlie Fig.S3F and S4.

Response: We thank the reviewer for this suggestion. We have now added supplementary tables providing detailed microglia cluster annotations as well as the DE analyses underlying Fig. S3F (revised Supplementary Fig.3F) and Fig. S4 (revised Supplementary Fig.5) (please see revised Supplementary Table 3 and Supplementary Table 4).

3. Somehow TREM2 is always spelled TREMG2, also in references.

Response: We thank the reviewer for pointing out this error. The spelling of TREM2 has now been corrected throughout the manuscript, including the main text, figure legends, and the reference list. We have carefully checked the revised version to ensure consistent and accurate usage of the correct gene name.

4. Fig.1: please add details on the axis of most figures or define in Fig. legend. E.g. Fig.1a: are these normalized counts, expression values, RKPM? Fig.1F: what is the surface measure?

Response: We appreciate the reviewer for pointing out the lack of clarity in the axis definitions and figure descriptions. We have revised Fig. 1 and the corresponding figure legends to clearly define all axes and quantitative measures. For Fig. 1A, we have clarified that the expression levels are derived from the AlzData portal based on the published single-cell RNA-seq dataset (GSE67835) and are presented as log-transformed TPM expression values. This information has now been explicitly indicated on the y-axis. For Fig. 1F, we have clarified that the surface measure refers to the quantified CD31-positive signal within the defined region of interest, reflecting the relative density of CD31 immunofluorescence. This definition is now explicitly indicated on the y-axis. In addition, we have ensured that all remaining panels in Fig. 1 include clear definitions of the plotted metrics, either directly on the axes or in the corresponding figure legends. These revisions were made to improve clarity, transparency, and ease of interpretation.

5. The color legend is missing for Fig.S5C-E, thus I cannot interpret which violin belongs to which genotype.

Response: We thank the reviewer for pointing out this omission. We have now added a color legend to the revised Supplementary Fig. 6C–E (previously Fig. S5C–E) to clearly indicate which violin plots correspond to each genotype. The figures have been revised accordingly to allow unambiguous interpretation.

6. Fig. 3D/E: given the difficulty with resolving individual cell bodies in plaque surrounding microglia: how did the authors manage to count those?

Response: We appreciate the reviewer's question regarding the quantification of microglia surrounding amyloid plaques. We agree that resolving individual microglial cell bodies in plaque-associated regions can be technically challenging due to dense processes and signal overlap. To address this, microglial quantification was performed using a combination of imaging and analysis strategies. First, imaging parameters, including exposure and gain, were carefully optimized to allow clear visualization of microglial somata in addition to their processes. Second, microglia were co-stained with DAPI, and the presence of a clearly identifiable nucleus was used as an additional criterion to define individual microglial cells. Only IBA1-positive cells with an associated DAPI-positive nucleus were counted as individual microglia. This combined approach enabled reliable identification and counting of microglial cell bodies in plaque-adjacent regions despite the dense microglial network. The same

acquisition and analysis criteria were applied consistently across all experimental groups.

7. Fig.4B-C: what is meant with ‘gene ratio’ on the x-axis?

Response: We appreciate the reviewer's comment. “Gene ratio” on the x-axis denotes the proportion of input genes that are annotated to a given enriched term, calculated as the number of DEGs overlapping with the term divided by the total number of DEGs used for the enrichment analysis (i.e., GeneRatio = Count / N_input).

8. P.14, lines 60-61: ‘MME was significantly elevated (Fig.4D)’ unless you provide p-values the reader cannot judge whether the elevation is indeed significant.

Response: We thank the reviewer for this comment. The statistical results for this comparison have now been included in Supplementary Table (please see revised Supplementary Table 3), where the p value is reported ($p = 3.73 \times 10^{-5}$), confirming that the elevation of MME is statistically significant.

9. Fig.4F: can you indicate which of these gene changes survive FDR correction by e.g. using asterisks?

Response: We thank the reviewer for this suggestion. Fig. 4F is intended to illustrate the overall expression patterns of Aβ-degradation–related proteins across groups rather than to serve as a statistical comparison panel. Statistical testing was performed separately for the relevant group comparisons, and the results after FDR correction have now been compiled into a supplementary table listing adjusted P values for each gene. To preserve the visual clarity of the heatmap, we did not add asterisks directly onto the figure. Instead, the full statistical results have been provided in the Supplementary Table (please see Supplementary Table 3).

10. Can you disclose where the anti-CD31 antibody binds (which amino acids)? Just to understand whether pre-incubation with the P1 fragment, does not lead to detection of P1, instead of full length CD31 when assessing the CD31 load in Fig. S6A.

Response: We thank the reviewer for this question. The anti-CD31 antibody used in this study is a monoclonal antibody generated against a synthetic peptide corresponding to residues surrounding Ala451 of the mouse CD31 protein. In contrast, P1 corresponds to the CD31 (722–738) fragment, which is located in a distinct and non-overlapping region of the protein. Therefore, this anti-CD31 antibody does not recognize or bind the P1 fragment.

11. P19, line 522: STAT1 or STAT3?

Response: Thank you for pointing this out. This was an inadvertent typographical error. The correct term is STAT3 (not STAT1). We have corrected this in the revised manuscript (please see page 21, line 569).

12. Please detail the exact mouse strain (catalog number) that was used from Cyagen Biosciences.

Response: The mouse strain used in this study was obtained from Cyagen Biosciences with the catalog number KOAI171018JW1. This information has been added to the revised manuscript (please see page 30, line 823).

Reviewer #3 (Remarks to the Author):

This study by Zhou et al. investigates the role of microglial CD31 in Alzheimer's disease pathogenesis. CD31 has long been recognized as an endothelial marker, whereas its expression and function in microglia are largely unknown. By analyzing published human and mouse single cell RNAseq datasets, the authors uncovered CD31 expression in microglia but not in neurons and astrocytes. Intriguingly, its expression was upregulated in the microglia of AD brains. To investigate its function in microglia in the setting of amyloid deposition, the author generated microglia-specific CD31 haploinsufficiency 5xFD mice. Through single-nucleus RNA sequencing, immunohistological analysis, and behavioral studies, the author demonstrated that deleting one allele of CD31 in microglial improves cognitive function, reduced Abeta load, confer microglia into homeostatic state. Mechanistically, the author demonstrated CD31 functions through STAT3/MME pathway. Overall, the paper is novel, and the results support the conclusion that microglial CD31 promotes AD pathology by suppressing STAT3 activation and reducing Aβ clearance.

Major Concerns:

1. The paper only included Cx3cr1Cre;CD31fl/+ heterozygous knockdown. The author should at least discuss the reason why they did not include homozygous knockout in addition to single allele deletion.

Response: We appreciate the reviewer's suggestion. The primary reason for not including a homozygous knockout model is the substantial time required to obtain Cx3cr1CreCD31fl/fl mice, particularly after multiple rounds of breeding and backcrossing, which would have significantly prolonged the experimental timeline. In addition, our data indicate that heterozygous CD31 deletion is already sufficient to produce robust and consistent phenotypic and pathological improvements. Therefore, to ensure experimental feasibility while still addressing the key biological question, we focused on the heterozygous knockdown model in the present study. We have now clarified this rationale in the revised manuscript (please see page 22, lines 581-585).

2. CD31 Knockdown reduces microglial numbers in 5xFAD mice. The authors interpreted this finding as reduced microglial activation. This is inaccurate. Microglial activation refers the cell

state not cell number. On the other hand, it would be good to evaluate whether the reduced cell number is due to decreased proliferation or increased apoptosis.

Response: We thank the reviewer for the clarification and fully agree that a reduction in microglial number should not be equated with reduced microglial activation, as activation primarily refers to changes in cellular state rather than cell abundance. Accordingly, we have removed the inaccurate description linking decreased microglial number to reduced activation from the revised manuscript (please see page 8-9, line 197-198).

In our single-nucleus transcriptomic analysis, we observed that the proportion of the MG8 microglial subcluster—characterized as a proliferative population—was lower in the CD31 knockdown group compared with 5xFAD controls (please see revised Fig. 2B). This finding suggests that CD31 modulation may affect microglial proliferation. To directly address the reviewer's suggestion regarding proliferation versus apoptosis, we performed additional immunofluorescence co-staining analyses. The number of TUNEL-positive microglia was not altered, indicating that microglial apoptosis or cell death was not significantly affected (please see revised Supplementary Fig. 4A). In contrast, the number of Ki67-positive microglia was significantly reduced following CD31 knockdown (please see revised Supplementary Fig. 4B), supporting the conclusion that CD31 knockdown primarily suppresses microglial proliferation. These new results have been incorporated into the revised Results section and are presented in the Supplementary Figures (please see page 9, 198-202).

Together, these findings indicate that the reduced microglial number observed upon CD31 knockdown is mainly attributable to decreased proliferation secondary to alleviated Aβ pathology, rather than enhanced microglial apoptosis.

3. It would be important to clarify whether P1 influences endothelial CD31 function, given that 81% of brain CD31 is in non-microglial cells, largely endothelial cells.

Response: We agree with the reviewer that it is important to clarify whether P1 affects endothelial CD31 function, given that the majority of brain CD31 is expressed by endothelial cells. CD31 is a well-established endothelial adhesion molecule, whose primary functions in endothelial cells include maintaining cell–cell adhesion, vascular integrity, and barrier stability.

To directly address whether P1 interferes with these endothelial CD31–related functions, we performed additional experiments to assess cerebrovascular structure and permeability following P1 treatment. Cerebral vessels were labeled using FITC perfusion, and both the number of FITC-positive vessels and microvascular leakage were quantified. Our results show no significant differences in cerebral vessel density or vascular permeability between the P1-treated group and the Vec-treated group (please see revised Supplementary Fig.9D-F). These findings indicate that P1 does not measurably disrupt endothelial CD31–mediated vascular adhesion or barrier function under our experimental conditions.

4. The rationale for using 1 mM P1 in the in vivo experiments is unclear. Did the authors perform any dose–response analysis?

Response: We thank the reviewer for this question. We did not perform a formal dose–response analysis in the present study. The in vivo dose of P1 was estimated based on the effective concentration identified in vitro (100 μM) and the CSF volume in adult mice (approximately 35–45 μL). On this basis, administration of 5 μL of a 1 mM P1 solution was expected to yield an initial CSF concentration on the order of ~100 μM after dilution, thereby achieving a biologically relevant exposure comparable to that observed in cell-based assays while accounting for CSF dilution and turnover.

5. Although the authors demonstrate physical interactions among CD31, STAT3, and SHP2 using co-IP and PLA, the functional role of SHP2 in this pathway remains untested. The study does not assess SHP2 phosphatase activity, test whether SHP2 inhibition or knockdown recapitulates the consequences of CD31 deficiency or determine whether the P1 peptide disrupts SHP2 recruitment in addition to CD31–STAT3 binding. These experiments will make the involvement of SHP2 in mediating CD31-dependent suppression of STAT3 phosphorylation largely correlative, so additional functional validation is advised to substantiate the proposed CD31–SHP2–STAT3 signaling mechanism.

Response: We thank the reviewer for this comment. In the revised manuscript, we performed additional mechanistic and functional experiments to directly test the role of SHP2 in CD31-dependent suppression of STAT3 phosphorylation and to clarify whether P1 disrupts SHP2 recruitment.

First, to determine whether CD31 deficiency alters SHP2 activation status, we examined phosphorylation at the SHP2 activation site in primary microglia under CD31 knockdown conditions. CD31 knockdown did not affect phosphorylation at the SHP2 activation site or total SHP2 protein levels, indicating that CD31 deficiency does not modulate STAT3 phosphorylation by altering SHP2 intrinsic activation status. (See revised Supplementary Fig. 7A–B.)

Second, to investigate the role of SHP2 in CD31-dependent suppression of STAT3 phosphorylation, we treated BV2 microglial cells with the SHP2 inhibitor SHP099. Pharmacological inhibition of SHP2 led to a marked increase in STAT3 phosphorylation without affecting total STAT3 levels. Importantly, SHP2 inhibition phenocopied CD31 knockdown by enhancing Aβ degradation capacity in microglia. These results demonstrate that SHP2 is functionally required for CD31-mediated suppression of STAT3 phosphorylation and downstream Aβ processing. (See revised Supplementary Fig.7C–E.)

Third, to address whether the P1 peptide disrupts SHP2 recruitment in addition to blocking CD31–STAT3 interaction, we performed co-immunoprecipitation analyses to directly assess

complex composition. P1 treatment significantly reduced CD31–STAT3 interaction, while CD31–SHP2 interaction remained unchanged. These findings indicate that P1 specifically interferes with the CD31–STAT3 interface without disrupting SHP2 recruitment to CD31. Consistently, P1 treatment increased STAT3 phosphorylation levels. Together, these data clarify that P1 acts by uncoupling STAT3 from CD31 rather than by displacing SHP2 from the CD31 complex. (See revised Fig.7L–O.)

Collectively, these newly added experiments provide direct functional evidence that SHP2 mediates CD31-dependent suppression of STAT3 phosphorylation and clarify the mechanistic action of P1 within the CD31–SHP2–STAT3 signaling axis.

Minor comments:

Page 9 line 213: homeostatic cluster should be MG0, not MG1, since MG1 is DAM cluster.

Response: Thanks for pointing this out. The homeostatic microglial cluster should be labeled as MG0, whereas MG1 corresponds to the DAM cluster. This labeling error has been corrected on Page 10, line 243 in the revised manuscript.

Fig S5 C-E: It is not clear what the red, green, and blue colored violin plots are representing.

Response: We apologize for the lack of clarity. In Fig. S5C–E, the red violin plots represent the Ctrl group, the green violin plots represent the 5xFAD group, and the blue violin plots represent the CD31 knockdown group. A corresponding color legend has now been added to revised Supplementary Fig. 6C–E and clarified in the figure legend.

Response to Reviewers

Manuscript ID: NCOMMS-25-87008A

Title: Microglial CD31 Orchestrates Alzheimer's Pathology via the SHP2–STAT3–MME Axis to Restrain Aβ Clearance

We sincerely thank the editor and reviewers for their positive evaluation of our revised manuscript. We are pleased that the reviewers found the manuscript significantly improved and compelling. Below, we provide our responses.

Reviewer #1

Comment: Authors of this study have been quite responsive to previous critics and have addressed each point in a rigorous manner. I have no additional concern to this well revised manuscript. With these extensive revised text and additional results to support their conclusion, this manuscript has improved significantly. IMO, this study is timely and highly important to the field. There is no doubt that authors have identified a novel SHP2–STAT3–MME Axis, regulated by CD31, in the control of clearance of Ab aggregates. This exciting finding paves a new path for future therapeutic intervention for AD treatment.

Response: We sincerely thank the reviewer for the positive and encouraging evaluation of our work. We are grateful for the recognition of the significance and rigor of our study.

Reviewer #2

Comment: The authors have addressed all the comments I had in a satisfactory manner. If possible I would advise to reduce the discussion section somewhat to keep it more succinct and reader-friendly.

Response: We thank the reviewer for this suggestion. In the revised manuscript, we have further refined the Discussion section to improve clarity and conciseness. Specifically, we reduced repetitive descriptions of experimental results and streamlined the mechanistic interpretation, while preserving the key conceptual advances of the study. These revisions have improved the overall readability of the manuscript.

Reviewer #3

Comment: The authors have done an excellent job addressing my concerns. The revised manuscript is compelling and will be of strong interest to both readers and the field.

Response: We sincerely thank the reviewer for the positive and supportive comments. We are grateful for the recognition of the quality and impact of our work.

We appreciate the reviewers' constructive feedback and believe that the manuscript is now suitable for publication in Nature Communications.
